# The complete mitochondrial genome of the woodwasp *Euxiphydria potanini* (Hymenoptera, Xiphydrioidea) and phylogenetic implications for symphytans

**DOI:** 10.1038/s41598-022-21457-0

**Published:** 2022-10-21

**Authors:** Bia Park, Ui Wook Hwang

**Affiliations:** 1 Department of Biology, Teachers College and Institute for Phylogenomics and Evolution, Kyungpook National University, Daegu, 41566 Republic of Korea; 2 School of Industrial Technology Advances, Kyungpook National University, Daegu, 41566 Republic of Korea; 3 Institute for Korean Herb-Bio Convergence Promotion, Kyungpook National University, Daegu, 41566 Republic of Korea; 4 Phylomics Inc., Daegu, 41910 Republic of Korea

**Keywords:** Genetics, Molecular biology

## Abstract

The long-necked woodwasp superfamily Xiphydrioidea belongs to the suborder Symphyta (Hymenoptera). Here we newly characterize the complete mitochondrial genome of the South Korean *Euxiphydria potanini* (Xiphydriidae) using next-generation sequencing: 16,500 bp long with 84.27% A + T content and 37 typical mitochondrial genes including those encoding 13 PCGs, 2 rRNAs, 22 tRNAs, and one A + T rich region. We compare the patterns of symphytan mitochondrial gene arrangement with those of an ancestral insect form and found some synapomorphic rearrangements in phylogenetic context. We use a variety of nucleotide and amino acid sequence alignments (thirteen mtPCGs and/or eight nDNAs) alongside step-by-step exclusions of long-branched taxa to elucidate the phylogenetic position of Xiphydrioidea and phylogenetic relationships among the seven symphytan superfamilies, except for Anaxyeloidea of which no mtgenome was available. The monophyly of symphytan superfamilies (with weak support for Pamphilioidea), sister-group relationship of Xiphydrioidea and Cephoidea, and Symphyta being paraphyletic to Apocrita, etc. are consistently supported by maximum likelihood and Bayesian inference trees. We also discuss the problematic phylogenetic positions of Orussoidea and Siricoidea and propose a hypothetical scenario of morphological character transition during hymenopteran evolution based on morphological key characteristics, such as the cenchrus and the wasp-waist.

## Introduction

The order Hymenoptera, which includes sawflies, wasps, bees, and ants, is one of the most species-rich groups of insects, with over 146,000 extant species described to date^1,2^. Hymenopteran insects are traditionally classified into two suborders: Symphyta (sawflies and woodwasps with a broad waist) and Apocrita (parasitoid and predatory species with a constricted wasp-waist). They live in a variety of ways ranging from phytophagous to parasitoid to predatory^3^. Except for the family Orussidae, which is parasitoids of wood-boring insects, the members of the suborder Symphyta are herbivores and some feed on plants that are economically important. They comprise eight superfamilies, 15 families, 817 genera, and 8855 species^4^. Various systematic studies have been conducted on sawflies using morphological and molecular approaches^2,5–18^, whereas phylogenetic approaches based on the complete mitochondrial genome (mtgenome) of all eight sawfly superfamilies^4^ have rarely been used. For example, phylogenetic studies using mtgenomes have recently been performed on a few subgroups of sawflies, i.e., Cephinae^19^, Tenthredinidae^20^, or roughly examined with those of seven or fewer sawfly superfamilies in a few papers but not focused on the phylogeny^21–23^. Mtgenomes are becoming increasingly important for insect molecular phylogenetics and evolution, phylogeography, population genetics, taxonomy, etc.^19–30^, owing to characteristics, such as the abundance of mitochondria per cell, their simple structure, maternal inheritance, relatively high evolutionary rate, low level of recombination, and absence of introns.

**Figure 1:**
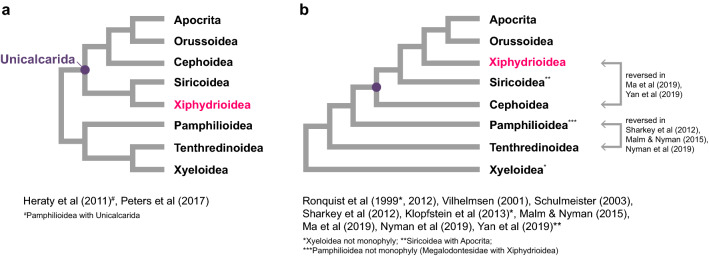
Conflicting hypotheses on phylogenetic relationships among the seven symphytan superfamilies except for Anaxyeloidea and the suborder Apocrita (see Fig. S1 for additional information).

The long-necked woodwasp superfamily Xiphydrioidea comprises 146 valid species in the world^4,31^. This superfamily includes only the family Xiphydriidae, the members of which are rarely collected in Malaise traps or with insect nets. Xiphydriids occur in most biogeographic regions such as the Palaearctic, Nearctic, Oriental, Neotropical, and Australasian regions except for the Afrotropic region, and the eastern Palaearctic and Oriental regions show the highest diversity. Xiphydriid larvae live in wood, with preference for weakened or dying small limbs of trees and shrubs^32^. The phylogenetic position of Xiphydriidae within Symphyta has been debated for decades: it is closely related to Cephidae in mtgenome-based phylogenies^22,23^, but cluster with Siricidae in phylogenies based on morphology^5–7^, four nuclear and one mitochondrial genes (*18S rRNA*, *28S rRNA*, *EF-1α*, and *COI*)^14^, and transcriptomes^17^. Other morphology and/or molecular phylogenetic studies^2,8–13,15,16,18^ showed that Xiphydriidae may be a sister to the clade of Orussidae and Apocrita (Figs. 1, S1).

Despite their widespread, almost cosmopolitan distribution and contentious phylogenetic position, few phylogenetic studies on Xiphydriidae have been conducted using mtgenomes. Here, the complete mtgenome of *Euxiphydria potanini* from South Korea is presented, which is the second published mtgenome of this family, after that of *Xiphydria* sp.^22^. Phylogenetic analyses on mtgenomes across Symphyta and with representatives of Apocrita as well were performed including the xiphydriid mtgenomes to examine the phylogenetic relationships between Xiphydriidae (Xiphydrioidea) and other symphytan superfamilies and families, with detailed characterization of the *E*. *potanini* mtgenome.

## Results and discussion

### Mtgenome organization and composition

**Figure 2:**
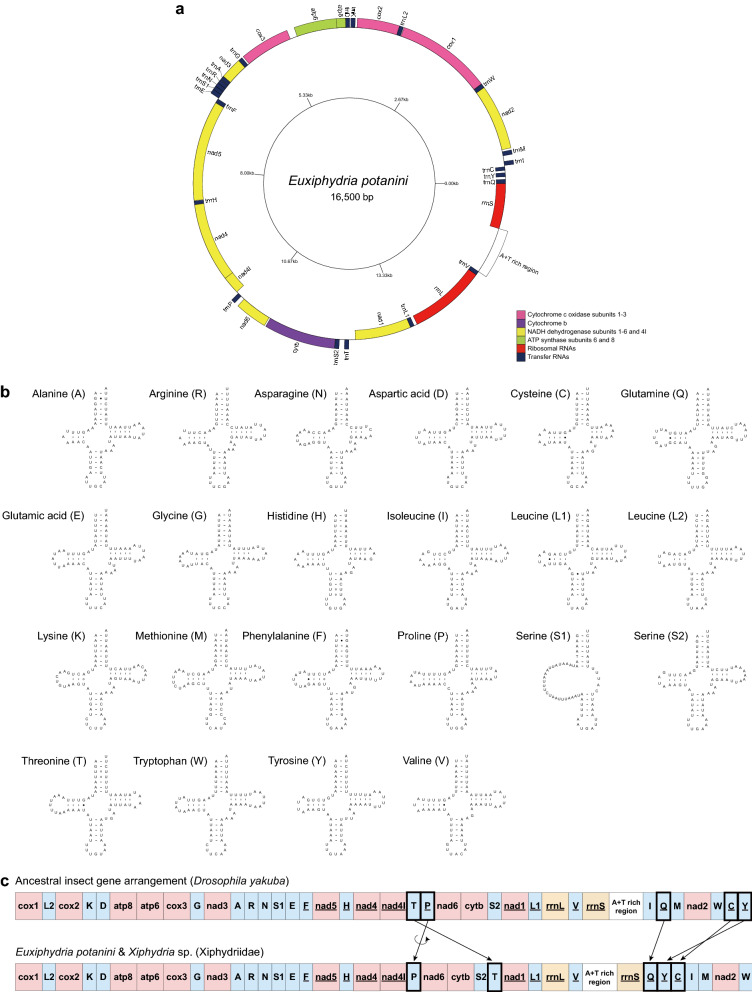
A newly sequenced mtgenome map and tRNA structures of *Euxiphydria potanini* (Xiphydriidae) and xiphydriid mitochondrial gene rearrangements from the ancestral form: (**a**) An *E*. *potanini* circular mtgenome map. PCGs and rRNA genes are shown with standard abbreviations, whereas tRNA genes are denoted by single-letter amino acid codes. Genes outside the map are coded on the H-strand and genes inside the map are coded on the L-strand; (**b**) Secondary structures of twenty-two *E*. *potanini* mitochondrial tRNAs predicted to be cloverleaf-shaped. Lines indicate A–U and G–C pairs, filled circles are G–U wobble binding, and empty circles represent no binding; (**c**) Comparison of mitochondrial gene arrangements of the two xiphydriid species with the ancestral insect form. The underlines represent genes encoded on the L-strand, arrows represent mitochondrial gene translocations, and the circle arrow indicates gene inversion. PCGs are indicated by red, rRNA genes by yellow, tRNA genes by blue, and an A + T rich region by white boxes.

The mtgenome of *E*. *potanini* (Xiphydriidae) comprises 16,500 bp (GenBank accession number OL639016), which is an intermediate length compared to other symphytan mtgenomes (average 16,410 bp; Table S1). This newly-sequenced mtgenome has the same standard gene component set as previously sequenced mtgenomes, including 13 PCGs, two rRNA genes—the large subunit rRNA gene (*rrnL*) and small subunit rRNA gene (*rrnS*), 22 tRNA genes, and an A + T rich region (Table 1, Fig. 2a). The heavy strand (H-strand) contains nine PCGs and 15 tRNA genes and the remaining four PCGs, two rRNA genes, and seven tRNA genes are on the light strand (L-strand).

**Table 1:** Summary of the mtgenome characteristics of *Euxiphydria potanini*.

Gene	Strand	Position		Length (bp)	Codon		Anticodon	IGN^a^
		From	To		Start	Stop		
*trnQ*	L	1	71	71			TTG	
*trnY*	L	116	182	67			GTA	44
*trnC*	L	222	283	62			GCA	39
*trnI*	H	307	374	68			GAT	23
*trnM*	H	470	539	70			CAT	95
*nad2*	H	573	1614	1042	ATG	T^b^		33
*trnW*	H	1615	1683	69			TCA	0
*cox1*	H	1692	3227	1536	ATA	TAA		8
*trnL2*	H	3239	3304	66			TAA	11
*cox2*	H	3305	3985	681	ATG	TAA		0
*trnK*	H	4020	4090	71			CTT	34
*trnD*	H	4108	4175	68			GTC	17
*atp8*	H	4176	4328	153	ATT	TAA		0
*atp6*	H	4322	5008	687	ATG	TAA		− 7
*cox3*	H	5132	5944	813	ATA	TAA		123
*trnG*	H	5972	6039	68			TCC	27
*nad3*	H	6040	6390	351	ATC	TAG		0
*trnA*	H	6389	6452	64			TGC	− 2
*trnR*	H	6459	6525	67			TCG	6
*trnN*	H	6524	6587	64			GTT	− 2
*trnS1*	H	6588	6649	62			TGA	0
*trnE*	H	6650	6720	71			TTC	0
*trnF*	L	6739	6807	69			GAA	18
*nad5*	L	6814	8529	1716	ATT	TAA		6
*trnH*	L	8539	8601	63			GTG	9
*nad4*	L	8601	9961	1361	ATG	TA^b^		− 1
*nad4l*	L	9955	10,257	303	ATG	TAA		− 7
*trnP*	H	10,293	10,359	67			TGG	35
*nad6*	H	10,429	10,953	525	ATA	TAA		69
*cytb*	H	10,979	12,133	1155	ATG	TAA		25
*trnS2*	H	12,137	12,204	68			TGA	3
*trnT*	H	12,292	12,361	70			TGT	87
*nad1*	L	12,473	13,429	957	ATA	TAA		111
*trnL1*	L	13,430	13,496	67			TAG	0
*rrnL*	L	13,552	14,861	1310				55
*trnV*	L	14,861	14,927	67			TAC	− 1
A + T rich region		14,928	15,731	804				0
*rrnS*	L	15,732	16,500	769				0

^a^IGN refers to the number of intergenic nucleotides.

^b^Incomplete termination codon that is probably extended by posttranscriptional adenylation.

The total length of 13 PCGs is 11,265 bp, accounting for 68.3% of the entire length of the *E*. *potanini* mtgenome. Except for *atp8* and *nad5* with ATT and *nad3* with ATC, the most common start codon is ATG or ATA. With the exception of *nad3* with TAG, *nad2* with T (incomplete), and *nad4* with TA (incomplete), TAA is the most frequent stop codon (Table 1). In addition, six gene-overlapping regions were discovered among the PCGs: *atp8*-*atp6* (seven overlapped nucleotides), *nad4*-*nad4l* (seven), *nad3*-*trnA* (two), *trnR*-*trnN* (two), *trnH*-*nad4* (one), and *rrnL*-*trnV* (one). Mitochondrial *tRNAs* are 62–71 bp long. All tRNAs have a typical clover-leaf secondary structure, except for tRNA-S1 lacking stable dihydrouridine (DHU) and pseudouridine (TΨC) arms, which are generally found in metazoan mitochondrial tRNAs^33^ (Fig. 2b). The acceptor stem, anticodon arm, and anticodon loop are highly conserved structures, based on predicted tRNA secondary structures, as opposed to the variable DHU and TΨC arms and loops. The lengths of *rrnL* (between *trnL1* and *trnV*) and *rrnS* (between an A + T rich region and *trnQ*) are 1310 bp and 769 bp, respectively. The nucleotide composition of the *E*. *potanini* mtgenome is 42.23% for A, 6.21% for G, 9.52% for C, and 42.04% for T, indicating AT bias (84.27% A + T content) (Table 2). As in the entire mtgenome sequences, all PCGs, rRNA genes, and tRNA genes are also AT-biased, as is commonly observed in hymenopteran insects^34^. In addition, the rRNA genes (88.07%) and tRNA genes (88.57%) showed slightly higher A + T content than PCGs (81.62%). The entire mtgenome, PCGs, and tRNA genes exhibited positive AT-skews (0.002–0.040), whereas those of rRNA genes were negative. All show negative GC-skew values (− 0.150 to − 0.315).

**Table 2:** The length, nucleotide composition, and AT/GC skewness of the *Euxiphydria potanini* mtgenome.

Region	Length (bp)	Nucleotide composition (%)						AT skew	GC skew
		A	G	C	T	A + T	G + C		
Entire mtgenome	16,500	42.23	6.21	9.52	42.04	84.27	15.73	0.002	− 0.211
PCGs	11,265	40.94	7.35	11.03	40.67	81.62	18.38	0.003	− 0.200
rRNA genes	2,079	42.57	4.09	7.84	45.50	88.07	11.93	− 0.033	− 0.315
tRNA genes	1,478	46.08	4.80	6.50	42.49	88.57	11.30	0.040	− 0.150

### Mitochondrial gene rearrangement

The *E*. *potanini* mitochondrial gene order is identical to that of *Xiphydria* sp. (MH422969), which has undergone five tRNA gene translocations and one inversion from the typical ground pattern of insect gene arrangement represented by *Drosophila yakuba*^35,36^. Five tRNA genes (*trnT*, *trnP*, *trnQ*, *trnC*, and *trnY*) were translocated and *trnP* was inverted (Fig. 2c). The mitochondrial gene order of *E*. *potanini* was rearranged into *nad4l*-*trnP*-*nad6*-*cytb*-*trnS2*-*trnT*-*nad1* and *trnQ*-*trnY*-*trnC*-*trnI*-*trnM*-*nad2*-*trnW*, which appear to be synapomorphic characteristics for the subfamily Xiphydriinae or possibly for the family Xiphydriidae because of the lack of such gene rearrangement events in other symphytan groups.

### Sequence alignment matrices and symphytan phylogeny with long-branch attraction

Based on the nucleotide and amino acid sequences of 13 mtPCGs obtained from a total of 123 taxa including 59 sawflies, 60 apocritan species, and four nonhymenopteran outgroups, we performed phylogenetic analyses among hymenopteran species (Table S1, Fig. S2). Maximum likelihood (ML) trees were reconstructed using the IQ-TREE web server^37^ based on nucleotide and amino acid sequence alignment sets (4909 nucleotide sites and 2000 amino acid sites in length), showing that the suborder Symphyta is paraphyletic to the suborder Apocrita, as reported in most previous studies^2,10–18,21–23^, and Xiphydrioidea is monophyletic and placed as a sister to the clade comprising Cephoidea, Orussoidea, Siricoidea, and Apocrita. Although the ML tree inferred from amino acid sequences did not support monophyly of Pamphilioidea (Fig. S2b), all other symphytan superfamilies formed monophyletic groups. Xyeloidea is placed as a sister to all other Hymenoptera. Orussidae (Orussoidea) and Siricidae (Siricoidea), two phylogenetically debated symphytan families, were mostly placed within or close to the Apocrita.

We examined the heterogeneous nucleotide sequence divergence and alignment ambiguity of the 123-taxa dataset using AliGROOVE^38^ to identify long-branched or ambiguously aligned taxa and minimize the effects of long-branch attraction artifacts (Fig. S3a). Some taxa with heterogeneous sequence divergence and alignment ambiguity were identified and removed from the initial dataset, i.e., one orussid (*Orussus occidentalis*), two siricids (*Tremex columba* and *T*. *fuscicornis*), and 60 apocritans (all examined here). Finally, we created more reliable nucleotide and amino acid sequence alignment sets (Matrices Mnt and Maa, respectively) with only 56 sawflies and four outgroups (Table 3). To examine the phylogenetic positions of the orussid and siricid, we made additional alignment sets with Orussoidea and Siricoidea, although they may suffer from long-branch attraction artifacts (Fig. S3a): Mnt + O and Maa + O with Orussoidea, Mnt + OS and Maa + OS with Orussoidea and Siricoidea. To reconfirm the phylogenetic relationships of symphytan superfamilies and families, we constructed additional alignment sets containing 13 mtPCGs and eight nuclear genes (CAD, GLN, GS, IDH, NAK, PGD, POL, and TPI^16^) from 25 sawflies (MNnt and MNaa), and subsequently produced MNnt + O, MNaa + O, MNnt + OS, and MNaa + OS (Table 3). With the exception of Orussoidea and Siricoidea, we confirmed that the constructed nucleotide sequence alignments are unambiguously aligned using additional AliGROOVE tests (Fig. S4). Model-based saturation plots were from all alignment sets listed in Table 3 to examine the respective degree of saturation, which showed that all alignment sets contained sufficient phylogenetic information with high R^2^ values (over 0.9247), with the exception of Mnt + OS (R^2^ = 0.8715) and Maa + OS (R^2^ = 0.9073), which are relatively lower than the others (Figs. S3b, S5). This demonstrated that the constructed alignment sets are not saturated and could be used to produce reliable phylogenetic trees for symphytans.

**Table 3:** Characteristics of fourteen nucleotide sequence and amino acid sequence matrices used in the current phylogenetic analyses, consisting of mtgenomes only or mtgenomes plus eight nDNAs.

Matrices	No. of ingroup taxa	Genes	Original lengths	Gblocks		Final lengths
				Discarded nucleotides or amino acids	Fully excluded genes	
Mnt	56 symphytans	13 mtPCGs	7964	1414	–	6550
Maa			3969	998	–	2971
MNnt	25 symphytans	13 mtPCGs + 8 nDNA^d^	11,095	1301	GS	9794
MNaa			4993	1014	GS	3979
Mnt + O^a^	57 symphytans	13 mtPCGs	7952	1437	–	6515
Maa + O			3963	1017	–	2946
MNnt + O	26 symphytans	13 mtPCGs + 8 nDNA	11,113	1393	GS	9720
MNaa + O			5002	1106	GS	3896
Mnt + OS^b^	59 symphytans	13 mtPCGs	7974	1795	–	6179
Maa + OS			3974	1116	–	2858
MNnt + OS	28 symphytans	13 mtPCGs + 8 nDNA	11,141	1423	–	9718
MNaa + OS			5016	1148	–	3868
Mnt + A^c^	56 symphytans + 54 apocritans	13 mtPCGs	8502	3333	–	5169
Maa + A			4238	2045	atp8	2193

^a^Inclusion of Orussoidea.

^b^Inclusion of Siricoidea.

^c^Inclusion of Apocrita.

^d^CAD, GLN, GS, IDH, NAK, PGD, POL, and TPI (see Malm and Nyman^16^ for the detailed information).

### Symphytan phylogeny removing long-branched taxa

**Figure 3:**
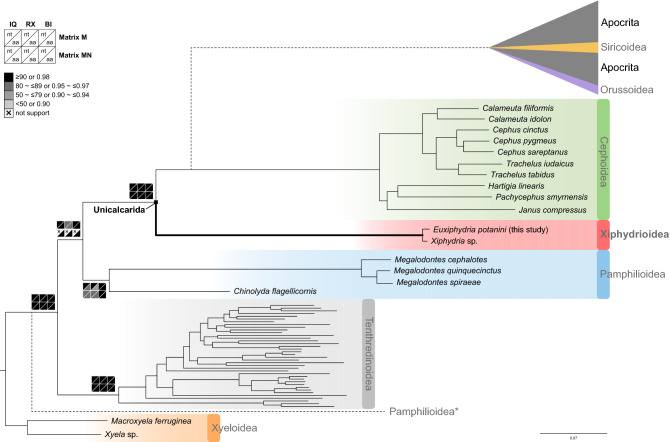
ML tree showing the phylogenetic position of Xiphydrioidea within Symphyta based on the mitochondrial nucleotide sequence alignment set (Matrix Mnt). The tree was constructed using IQ-TREE web server^37^, and it is shown as a representative for twelve phylogenetic trees, including four IQ-TREE (IQ)-based ML, four RAxML (RX)-based ML, and four MrBayes-based BI trees inferred from Matrices Mnt, MNnt, Maa, and MNaa (Figs. S6–17). Node confidence values resulting from the twelve trees are plotted on the respective branches by black, dark gray, gray, and light gray boxes along with ranges of bootstrapping values or posterior probability as specified in the upper left corner. The IQ-based ML, RX-based ML, and BI trees produced based on MNnt showed that the monophyletic Pamphilioidea (asterisk) is placed as sister to all other hymenoterans except for Xyeloidea, whereas all other analyses supported that Pamphilioidea is a sister group of Unicalcarida in common. The group comprising Apocrita, Orussoidea, and Siricoidea is shown as a sister to Cephoidea, according to the topology shown in Fig. S2.

Along with the stepwise removal of possible long-branched taxa based on the AliGROOVE results (Figs. S3a, S4), we investigated the phylogenetic relationships among symphytan superfamilies and families using concatenated nucleotide and amino acid sequence alignment sets of mtPCGs only (Mnt and Maa) and of mtPCGs plus eight nuclear genes (MNnt and MNaa) (Table 3). ML trees were produced using the IQ-TREE web server^37^ and RAxML v.8.2.12^39^, and Bayesian inference (BI) trees using MrBayes 3.2.7a^40^ (Figs. 3, S6–17). The following best-fit substitution models were used for analyses: GTR + F + I + G4 (Mnt and MNnt) and mtART + F + I + G4 (Maa and MNaa) in IQ-TREE, GTR + F + G (Mnt and MNnt) and mtART + F + G (Maa and MNaa) in RAxML, and GTR + I + G (Mnt and MNnt) and mtREV + I + G (Maa and MNaa) in MrBayes.

Despite relatively weak support for Pamphilioidea monophyly (78–96 BP and 0.99–1.00 BPP), the resulting phylogenetic trees strongly supported the monophyly of symphytan superfamilies and families with high bootstrapping values (BP; above 96%) or Bayesian posterior probability (BPP; 1.00 in all trees) (Figs. 3, S6–17). The superfamily Xiphydrioidea formed a close relationship with the superfamily Cephoidea, without exception (100 BP and 1.00 BPP). The results also demonstrated that the family Xyelidae, which belongs to the superfamily Xyeloidea, occurred as the most basal branch, i.e., as a sister group to all other Hymenoptera. Except for the unstable phylogenetic position of Pamphilioidea and Tenthredinoidea, which were switched, the overall tree topology was highly conserved: (Xyeloidea, (Tenthredinoidea, (Pamphilioidea, (Cephoidea, Xiphydrioidea)))) or (Xyeloidea, (Pamphilioidea, (Tenthredinoidea, (Cephoidea, Xiphydrioidea)))). The former was supported by six ML and three BI trees (Figs. S6–11, S15–17) inferred from Mnt, Maa, and MNaa among eight ML and four BI trees, and the latter by the remaining two ML and one BI trees (Figs. S12–14), resulting in extremely low node confidence values between Pamphilioidea and (Tenthredinoidea, (Cephoidea, Xiphydrioidea)) (47–50 BP and 0.7 BPP). This suggests that the former may be a much more reliable relationship than the latter, with respect to the position of Pamphilioidea (Fig. 3).

### Phylogenetic relationships among the unicalcarid members

#### Phylogenetic position of Orussoidea

Although *Orussus occidentalis* was a long-branched taxon (Figs. S3a, S4c,d), to clarify the phylogenetic position of Orussoidea, we produced four additional alignment sets with *O*. *occidentalis* based on nucleotide sequences (Mnt + O and MNnt + O) and amino acid sequences (Maa + O and MNaa + O); the included taxa are listed in Table 3. Without Apocrita and Siricoidea among the members of Unicalcarida (Xiphydrioidea, Cephoidea, Orussoidea, Siricoidea, and Apocrita), the reconstructed phylogenetic trees showed that Orussoidea was closely related with Cephoidea (92–100 BP and 1.00 BPP) in the analyses (Figs. S18a, 20–31), and the overall tree topology was identical to that of the trees reconstructed without Orussoidea (Figs. 3, S6–17).

#### Problematic phylogenetic position of Siricoidea

**Figure 4:**
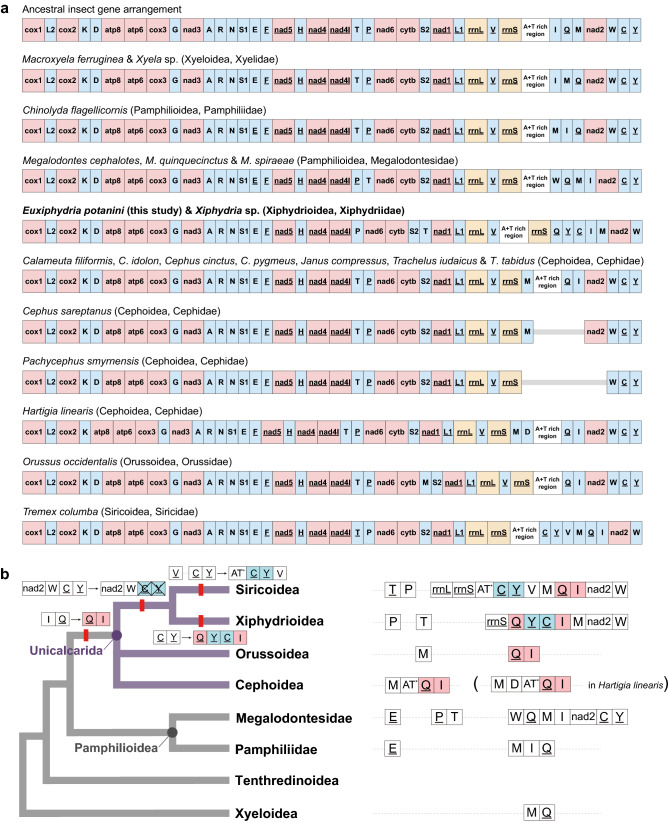
A comparison of mitochondrial gene arrangement patterns in six superfamilies as well as mitochondrial gene rearrangement during unicalcarid evolution: (**a**) Comparison of mitochondrial gene arrangement patterns of twenty sawflies in six superfamilies with an ancestral insect form; (**b**) Mitochondrial gene rearrangement during the unicalcarid evolution. Some mitochondrial gene rearrangements are shown on the symphytan phylogenetic tree, which may indicate possible synapomorphic characters supporting the monophyly of Unicalcarida (QI), the close relationship of Xiphydrioidea and Siricoidea (CY translocations), and the monophyly of Xiphydrioidea (YC between Q and I). The translocation and inversion of V occurred only in the lineage of Siricoidea. Asterisks indicate the A + T rich region.

In addition to *O*. *occidentalis*, two long-branched siricoids (*Tremex columba* and *T*. *fuscicornis*; Figs. S3a, S4e,f) were added to the data matrices to create new sequence alignment sets based on nucleotide sequences (Mnt + OS and MNnt + OS) and amino acid sequences (Maa + OS and MNaa + OS); the included taxa are listed in Table 3. Without Apocrita among the members of Unicalcarida, the reconstructed phylogenetic trees (Figs. S18b, 19, 32–43) showed that Siricoidea mostly clustered with Orussoidea (56–84 BP and 0.57–1.00 BPP; Figs. S32–40, 43) or less frequently with Xiphydrioidea (58–62 BP; Figs. S41, 42). The sister group relationship between Orussoidea and Siricoidea may be due to the long-branch attraction artifact between the two, although the majority of the trees supported this relationship from the matrices of Mnt + OS, Maa + OS, MNnt + OS, and MNaa + OS (only in BI). By contrast, despite the support of only a few trees from MNaa + OS (only in ML), the relationship between Xiphydrioidea and Siricoidea has been advocated and suggested by a few reports based exclusively on molecular data, such as nuclear and mitochondrial genes or transcriptomes^14,17^ (Figs. 1a, S1b). Non-sequence data, such as mitochondrial gene rearrangement patterns, may help support the close relationship between Xiphydrioidea and Siricoidea (Fig. 4). We compared the mitochondrial gene arrangement patterns among 20 sawflies from six superfamilies with the ancestral insect form (Fig. 4a). Based on gene rearrangements, in unicalcarids, I–Q was rearranged into Q–I compared to common ancestral insects, possibly supporting the monophyly of Unicalcarida (exceptionally found in Megalodontesidae). Furthermore, C–Y translocation from W–C–Y occurred commonly in Xiphydrioidea and Siricoidea, which might be interpreted as a possible synapomorphic characteristic of the two groups. In addition, we found some lineage-specific characteristics: C–Y was rearranged into Q–Y–C–I and AT–C–Y–V in Xiphydrioidea and Siricoidea, respectively. V was inverted and translocated between Y and M in Siricoidea (Fig. 4b).

### Possible evolutionary scenario of morphological character transitions regarding the loss of cenchrus and the acquisition of the wasp-waist in Hymenoptera

**Figure 5:**
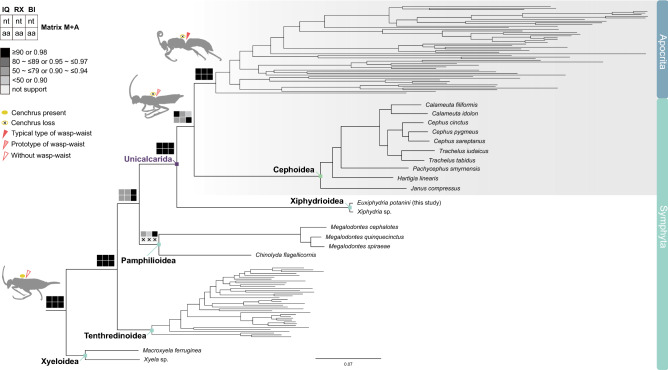
ML tree showing phylogenetic relationships of hymenopteran superfamilies without Orussoidea and Siricoidea based on the mitochondrial nucleotide sequence alignment set (Mnt + A) with the depiction of morphological character transitions of the cenchrus and wasp-waist during the hymenopteran evolution. The tree was constructed using IQ-TREE web server^37^ and it is shown as a representative for six phylogenetic trees including IQ-TREE (IQ)-based ML, RAxML (RX)-based ML, and MrBayes-based BI trees from Matrices Mnt + A and Maa + A (Figs. S44–49). Node confidence values resulting from the six trees are plotted on the respective branches of the ML tree by black, dark gray, gray, and light gray boxes along with ranges of bootstrapping values or posterior probability as specified in the upper left corner.

After the removal of nine possible long-branched species (one orussid, two siricids, and six apocritans) from a total of 123 taxa (Table S1, Fig. S3a), phylogenetic analyses were conducted based on nucleotide and amino acid sequences of 13 mtPCGs obtained from a total of 110 hymenopteran ingroup species, including 56 symphytans and 54 apocritans, and four nonhymenopteran outgroups (Figs. 5, S44–49). The six resultant phylogenetic trees based on Matrices Mnt + A and Maa + A (Table 3, Table S1, Figs. S44–49) consistently supported that the suborder Symphyta is paraphyletic to the suborder Apocrita, with the superfamily Xyeloidea as a sister group of all other Hymenoptera, which is in line with traditional views and most previous studies^2,8–13,15,16,18,22,23^ (Figs. 1b, S1c). They robustly supported the monophyly of four symphytan superfamilies with high node confidence values (97–100 BP and 1.00 BPP), except for the relatively lower nodal values (44–53 BP and 1.00 BPP), or in rare cases, they did not support monophyly of Pamphilioidea from amino acid sequence data (Figs. 5, S47–49). The overall tree topology was highly conserved except for the unstable monophyly of Pamphilioidea: (Xyeloidea, (Tenthredinoidea, (Pamphilioidea, (Xiphydrioidea, (Cephoidea, Apocrita))))) in trees based on nucleotide sequences or (Xyeloidea, (Tenthredinoidea, (Pamphiliidae, (Megalodontesidae, (Xiphydrioidea, (Cephoidea, Apocrita)))))) in trees based on amino acid sequences. Within the Unicalcarida without Orussoidea and Siricoidea, Xiphydrioidea as a sister group of the clade of Cephoidea and Apocrita were supported by all the resultant trees with high node confidence (98–100 BP and 1.00 BPP), and Cephoidea were clustered with Apocrita with relatively lower nodal supports (53–92 BP and 0.87–0.99 BPP) (Figs. 5, S44–49). Regarding the superfamilies Orussoidea and Siricoidea, which have a parasitoid lifestyle and a unique molecular evolutionary pattern^22,23,41,42^ respectively, clarifying their stable phylogenetic positions is likely difficult using the mtgenome marker. Some evidence has been reported indicating the sister group relationships between Orussoidea and Apocrita^2,8–13,15,17,21,22^ and between Siricoidea and Apocrita^23^. Instead, Orussoidea and Siricoidea were placed within the monophyletic apocritan clade (Fig. S2), which can be interpreted as long-branch attraction artifacts (Figs. S3a, S4c–f). It implies that their phylogenetic positions within Apocrita may not be reliable. With the exclusion of the two problematic taxa is excluded, Cephoidea consistently appeared as a sister group of Apocrita. There are some morphological similarities between Cephoidea and Apocrita: the lack of the cenchrus, which is observed in all other symphytans, and the relatively constricted thorax and abdomen, referred to as wasp-waist, which is not observed in any of other symphytan. Based on these morphological similarities, we propose a morphological character transition scenario during hymenopteran evolution (Fig. 5). In this hypothetical scenario, apocritan-like characteristics, such as the lack of a cenchrus and the wasp-waist occurring in the Cephoidea belonging to the Symphyta, may be interpreted as intermediate characteristics while shifting from symphytans to apocritans. It seems to be consistent with the morphology-based views of classical reports such as those of Ross^6^ and Königsmann^7^, although they may be old fashioned.

The present study produced an extensive tree based on mtgenomes of the order Hymenoptera, which may help elucidate not only the phylogenetic position of Xiphydrioidea but also the controversial phylogenetic relationships between hymenopteran suborders, superfamilies, and even families. The broad framework and new perspectives on symphytan taxonomy and relationships based on mtgenomes provide valuable information for future research in the field of hymenopteran systematics.

## Materials and methods

### Sample collection and DNA extraction

An adult female *E*. *potanini* was collected using an insect net in Seosam-myeon, Jangseong-gun, Jeollanam-do, South Korea (May 30, 2020). The fresh sample was immediately preserved in 95% ethanol and was then identified using external morphological characteristics. Total genomic DNA was extracted from muscle tissue of the thorax using a DNeasy Blood & Tissue Kit (Qiagen, Hilden, Germany) according to the manufacturer’s instructions.

### Mtgenome sequencing, assembly, and annotation

The entire mtgenome was sequenced using an Illumina Hiseq 4000 or HiSeqX platform in 150 bp paired-end 5G mode (GnCBIO, Daejeon, South Korea). In total 47,281,982 raw paired-end reads were produced. The complete mtgenome of *E*. *potanini* was generated via de novo assembly using the next-generation sequencing (NGS) data with xiphydriid mtgenome deposited in NCBI (accession number MH422969). The raw paired-end data were produced from *E*. *potanini* (3.7 Gb). The high quality 61,787 reads (150 bp/read) were obtained after quality trimming. The cleaned reads were subjected to de novo assembly and scaffolding using CLC Genomics Workbench. After the assembly, 31,599 contigs (including 11,246,335 bp and N50 343 bp) were obtained. Finally, the scaffolded contig sequences were 16,492 bp. Also, we used the hybrid scaffolds for gapfilling using Gap close program in CLC Genomics Workbench. Finally, the completeness of the mtgenome was 99.8% (0.2% gap), which is estimated with the reference mtgenome.

All 13 PCGs, two rRNA genes, and 22 tRNA genes were determined by comparison with the respective homologous sequences of other xiphydriid species downloaded from GenBank (MH422969). The 13 PCGs were predicted by comparing them to the reference mtgenome and the translated nucleic acid sequences to their corresponding peptide sequences based on the invertebrate mitochondrial DNA genetic code using the EMBOSS transeq server^43^. ARWEN v1.2^44^ and tRNAscan-SE 2.0^45^ were used to identify the locations of 22 tRNAs. Their secondary structures were manually plotted using Adobe Illustrator 2021 according to ARWEN predictions. The two rRNA genes (*rrnS* and *rrnL*) and A + T rich region were determined based on the locations of adjacent genes (*trnL1* and *trnV*) and alignment with the reference mtgenome. Mitogenomic circular maps were visualized using GenomeVx^46^. The complete mtgenome sequence was deposited in GenBank under accession number OL639016.

### Sequence analyses and model-based saturation plot

The length, base composition, A + T and G + C contents, and skewness of the entire mtgenome, PCGs, rRNA genes, and tRNA genes were calculated using BioEdit v7.0.5.3^47^. Strand asymmetry was calculated using the formulas AT skew = [A − T]/[A + T] and GC skew = [G − C]/[G + C]^48^. The heterogeneity of sequence divergence within the four different datasets was analyzed using AliGROOVE^38^ with default sliding window size. The overall best-fitting model, GTR or mtART, was selected as the reference model for the saturation plots. Patristic distances were generated using PATRISTIC v.1.0^49^, which were derived from trees obtained under the observed distances (uncorrected p-distance) and plotted against mtART or GTR distances. The slope of the regression line in the plot was used as a measure of saturation.

### Sequence alignments and phylogenetic analyses

A total of 122 available mtgenomes were retrieved from GenBank to analyze their phylogenetic relationships (Table S1). A newly sequenced xiphydriid species (*E*. *potanini*), 58 symphytans, and 60 apocritans were selected as ingroups. Four species, *Paroster microsturtensis* (Watts & Humphreys) (MG912997) of the Coleoptera, *Anopheles gambiae* Giles (L20934) of the Diptera, *Neopanorpa pulchra* Carpenter (FJ169955) of the Mecoptera, and *Neochauliodes parasparsus* Liu & Yang (KX821680) of the Megaloptera, were used as outgroup taxa. Nucleotide sequence and amino acid sequence alignment sets of 13 mtPCGs and eight nuclear genes (CAD, GLN, GS, IDH, NAK, PGD, POL, and TPI^16^) were used to elucidate the phylogenetic relationships of superfamilies and families of the suborder Symphyta. Regarding nucleotide sequences of all mtPCGs, the first and second codon positions were used for phylogenetic analyses.

Seven different datasets were used to generate phylogenetic relationships (see Table 3 for additional information): (1) Matrix M based on the 13 mtPCGs of 60 species, including 56 symphytans without the members of Orussoidea and Siricoidea, and four nonhymenopteran outgroups; (2) Matrix MN based on the 13 mtPCGs plus eight nDNAs of 25 symphytans without the members of Orussoidea and Siricoidea; (3) Matrix M + O based on the 13 mtPCGs of 61 species, including 57 symphytans only with the Orussoidea and four nonhymenopteran outgroups; (4) Matrix MN + O based on the 13 mtPCGs plus eight nDNAs of 26 symphytans only with the member of Orussoidea; (5) Matrix M + OS based on the 13 mtPCGs of 63 species, including 59 symphytans with the members of Orussoidea and Siricoidea and four nonhymenopteran outgroups; (6) Matrix MN + OS based on the 13 mtPCGs plus eight nDNAs of 28 symphytans with the members of Orussoidea and Siricoidea; (7) Matrix M + A based on the 13 mtPCGs of 114 species, including 56 symphytans without the members of Orussoidea and Siricoidea, 54 apocritans, and four nonhymenopteran outgroups. When we built matrices that included mtPCGs and nDNAs, we merged sequence information from different species within the same family or genus in some taxa (see Table S2 for detailed information). Even though the merged sequences for a representative species were derived from different genera within the same family, there is no problem in elucidating phylogenetic relationships among higher taxonomic levels of symphytans (mainly between superfamilies or between families).

The nucleotide or amino acid sequences of only 13 mtPCGs or 13 mtPCGs plus eight nuclear genes were aligned independently using ClustalX 2.1^50^. From each of all alignment matrices of the nucleotide or amino acid sequences, highly conserved sequence alignment blocks were extracted using Gblocks 0.91b^51^ with default options. Then they were concatenated into an independent sequence alignment matrix. To reconstruct ML trees using the IQ-TREE web server^37^ and RAxML v.8.2.12^39^, we identified the best-fitting models selected by ModelFinder for all matrices: mtART + F + I + G4, GTR + F + I + G4 (IQ-TREE), and mtART + F + G, GTR + F + G (RAxML). To infer nodal support of the resulting trees, bootstrap support values were evaluated with 1000 replicates. The BI phylogenetic analysis was carried out using MrBayes 3.2.7a^40^ with the following settings: two independent runs of 10 million generations, including four Markov chains (three cold, one heated), sampling every 1000 generations, and a burn-in of 25% of the trees. The best-fit models for producing BI trees, mtREV + I + G and GTR + I + G, were selected. FigTree v.1.4.4 (http://tree.bio.ed.ac.uk/) was used to visualize the consensus phylogenetic trees.

## Supplementary Information

Supplementary Information.

## Data Availability

The mitochondrial genome of *Euxiphydria potanini* was deposited on NCBI GenBank under accession number OL639016.
