# Supplementary material for: The complete mitochondrial genome of the woodwasp Euxiphydria potanini (Hymenoptera, Xiphydrioidea) and phylogenetic implications for symphytans
Source: Sci Rep. 2022 Oct 21;12:17677. doi: 10.1038/s41598-022-21457-0 (PMC9587024; doi:10.1038/s41598-022-21457-0)
Supplement: Supplementary file 1 — Supplementary Information. [file 41598_2022_21457_MOESM1_ESM.pdf]

Supplementary Materials

The complete mitochondrial genome of the woodwasp *Euxiphydria potanini* (Hymenoptera, Xiphydrioidea) and phylogenetic implications for symphytans

Bia Park and Ui Wook Hwang

Figure S1 The conflicting hypotheses on phylogenetic relationships among the seven symphytan superfamilies and suborder Apocrita.

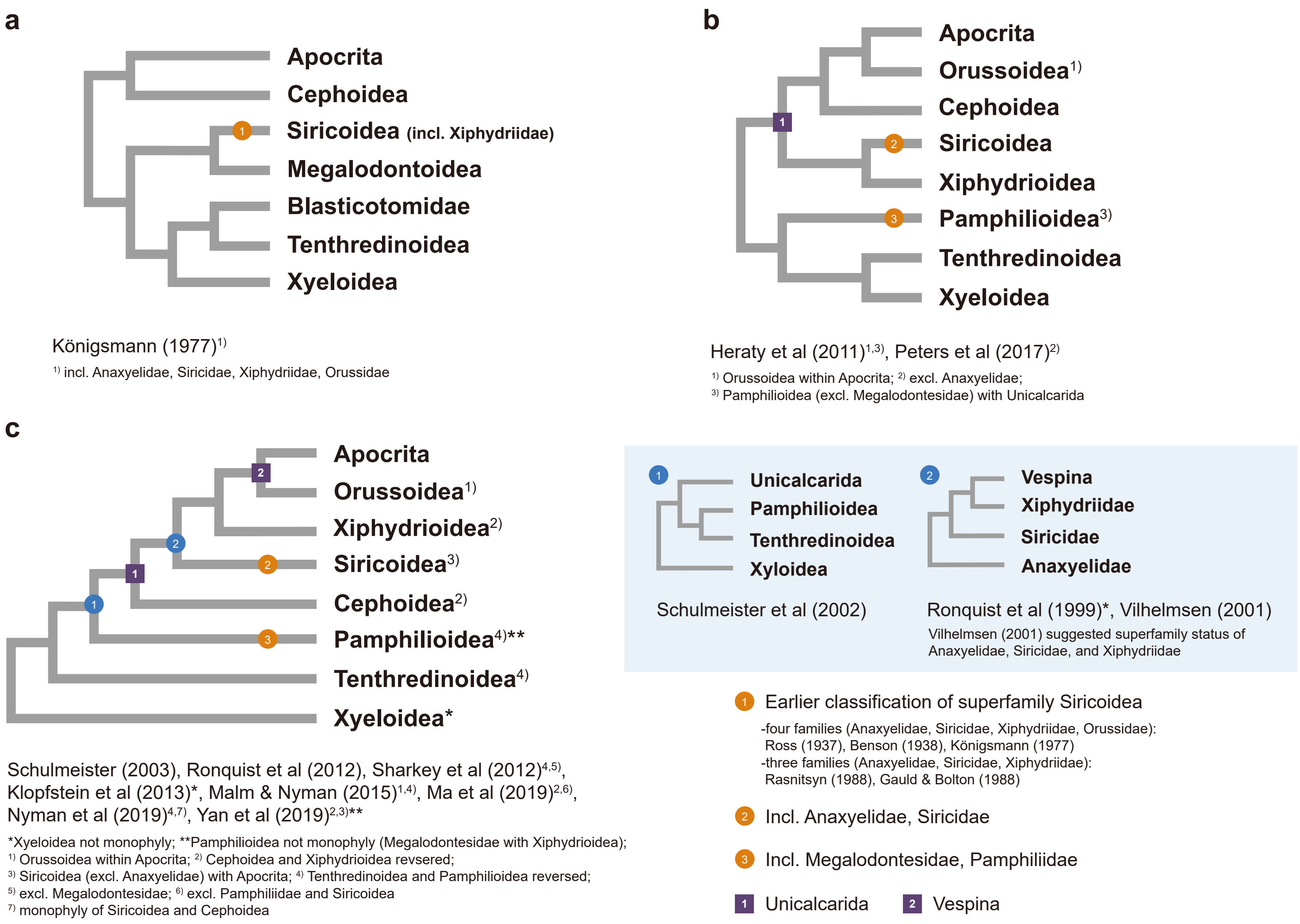

Figure S2 ML trees (IQ-TREE) based on the nucleotide (a) and amino acid (b) sequence alignment sets of 13 mtPCGs of the initial dataset including 123 taxa.

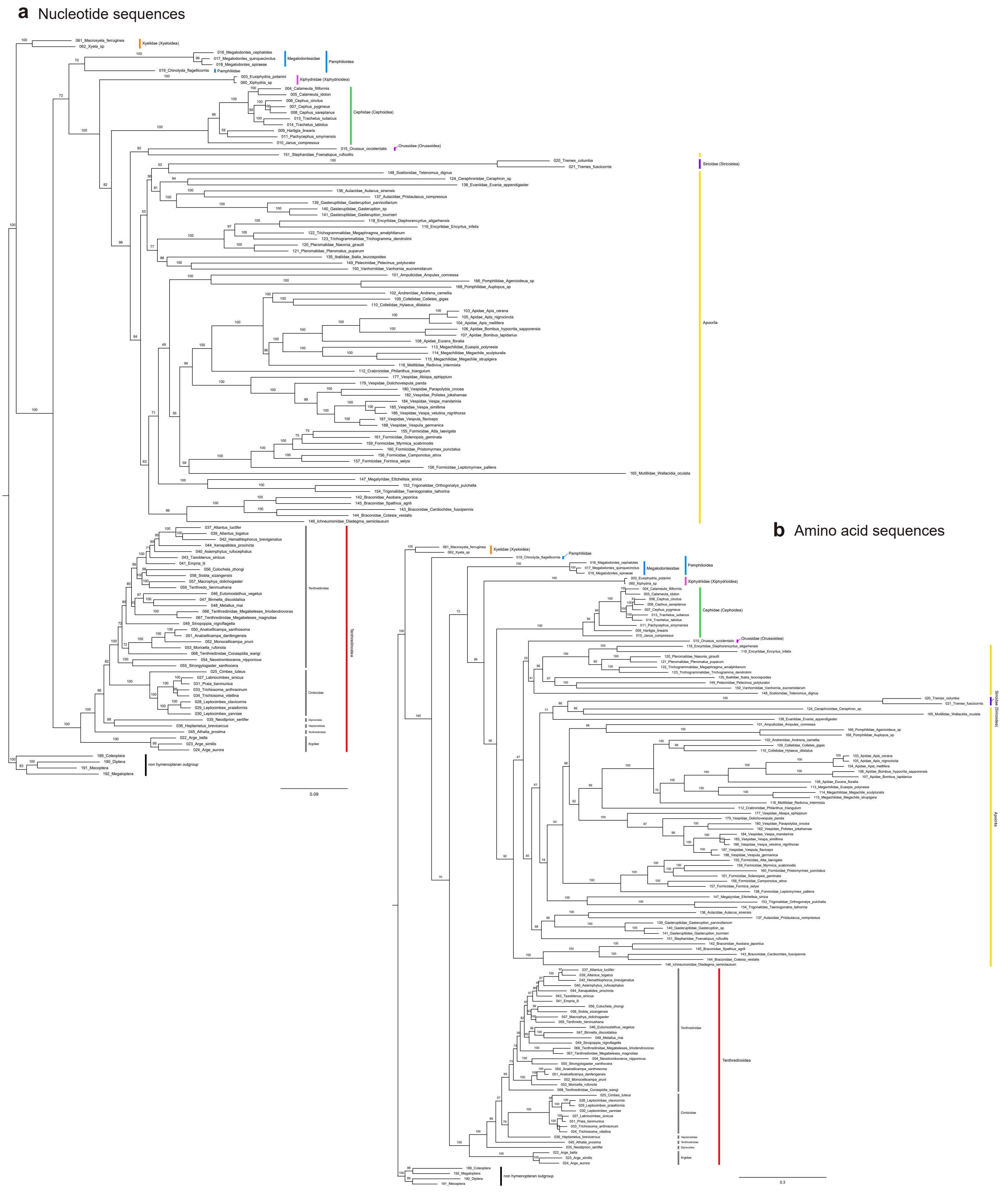

Figure S3 Examination of the unambiguously aligned taxa in the concatenated nucleotide sequences of 13 mtPCGs for the initial dataset using AliGROOVE (a) and model-based saturation plots based on the nucleotide and amino acid sequence alignment sets for the initial dataset (b).

a AliGROOVE

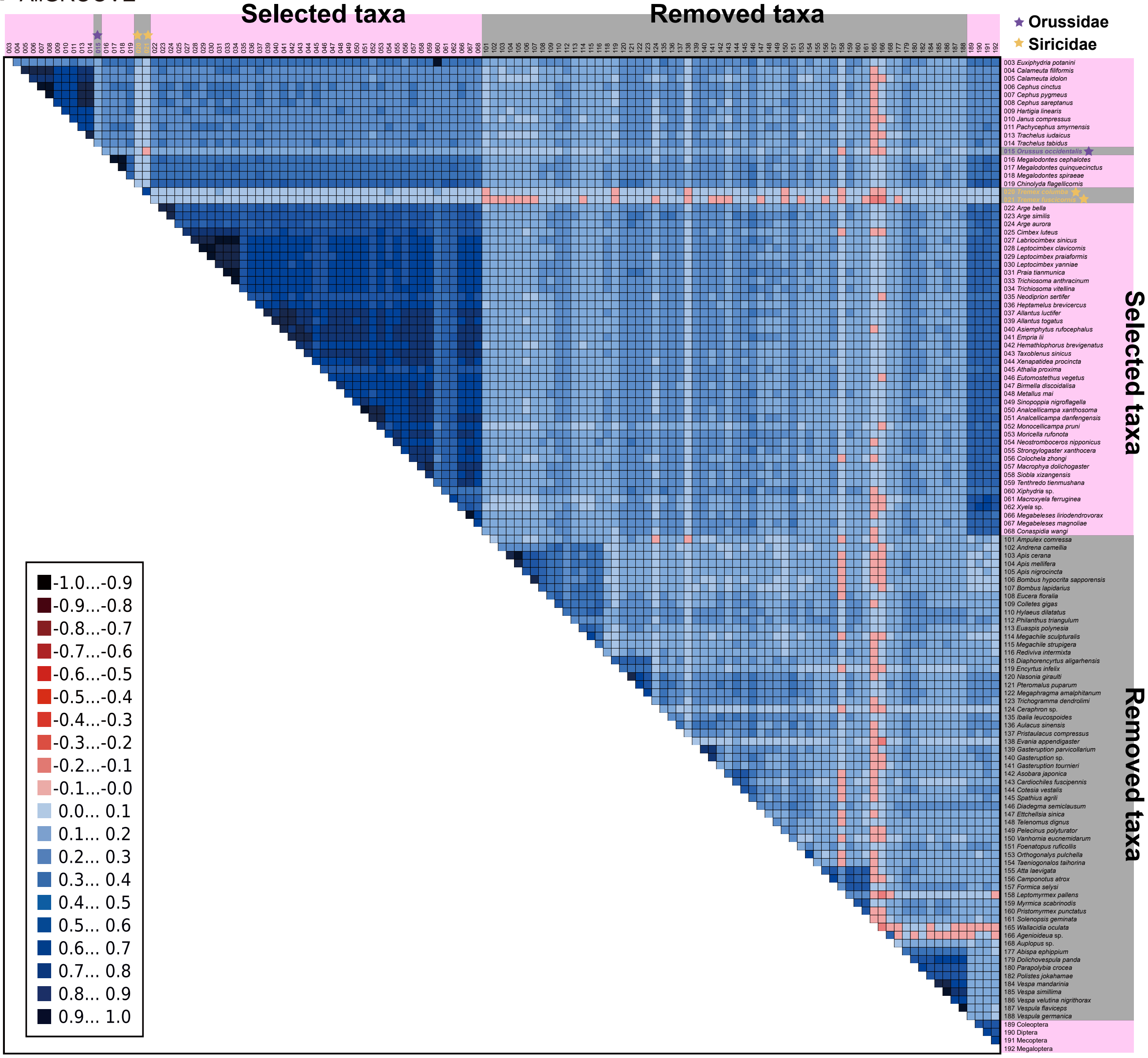

b Model-based saturation plots

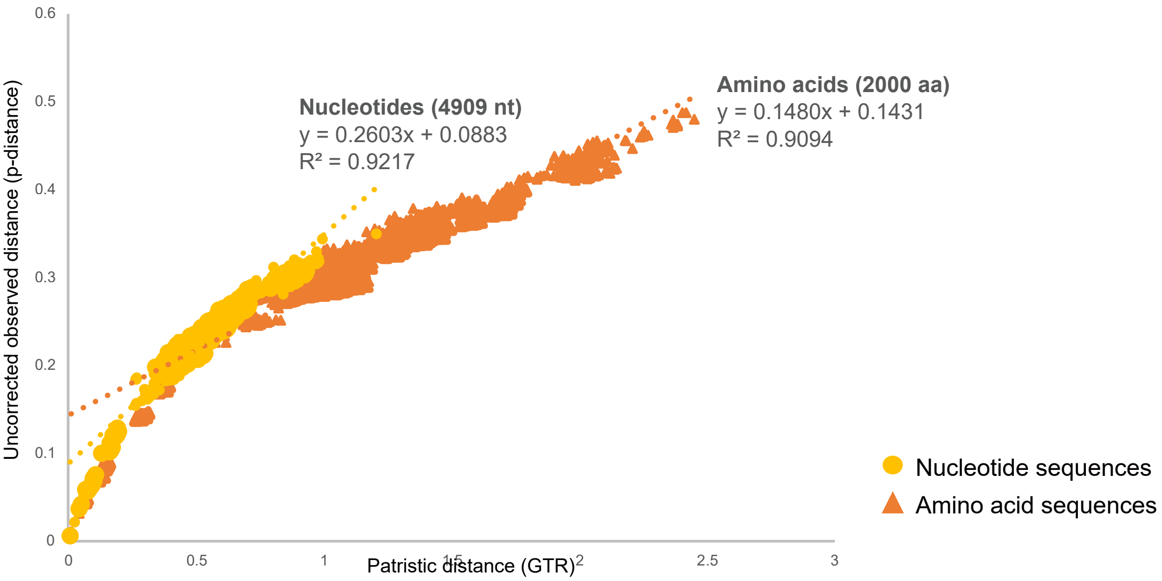

Figure S4 Examination of the unambiguously aligned taxa in the concatenated nucleotide sequences using AliGROOVE. The nucleotide sequence alignment sets were analyzed based on 13 mtPCGs for Matrices M, M+O, and M+OS, and 13 mtPCGs plus 8 nDNA for Matrices MN, MN+O, and MN+OS. Red blocks indicate minus values which represent ambiguous alignment blocks or long-branched blocks, whereas blue blocks show positive values which represent homogeneous sequence blocks or unambiguous alignment blocks.

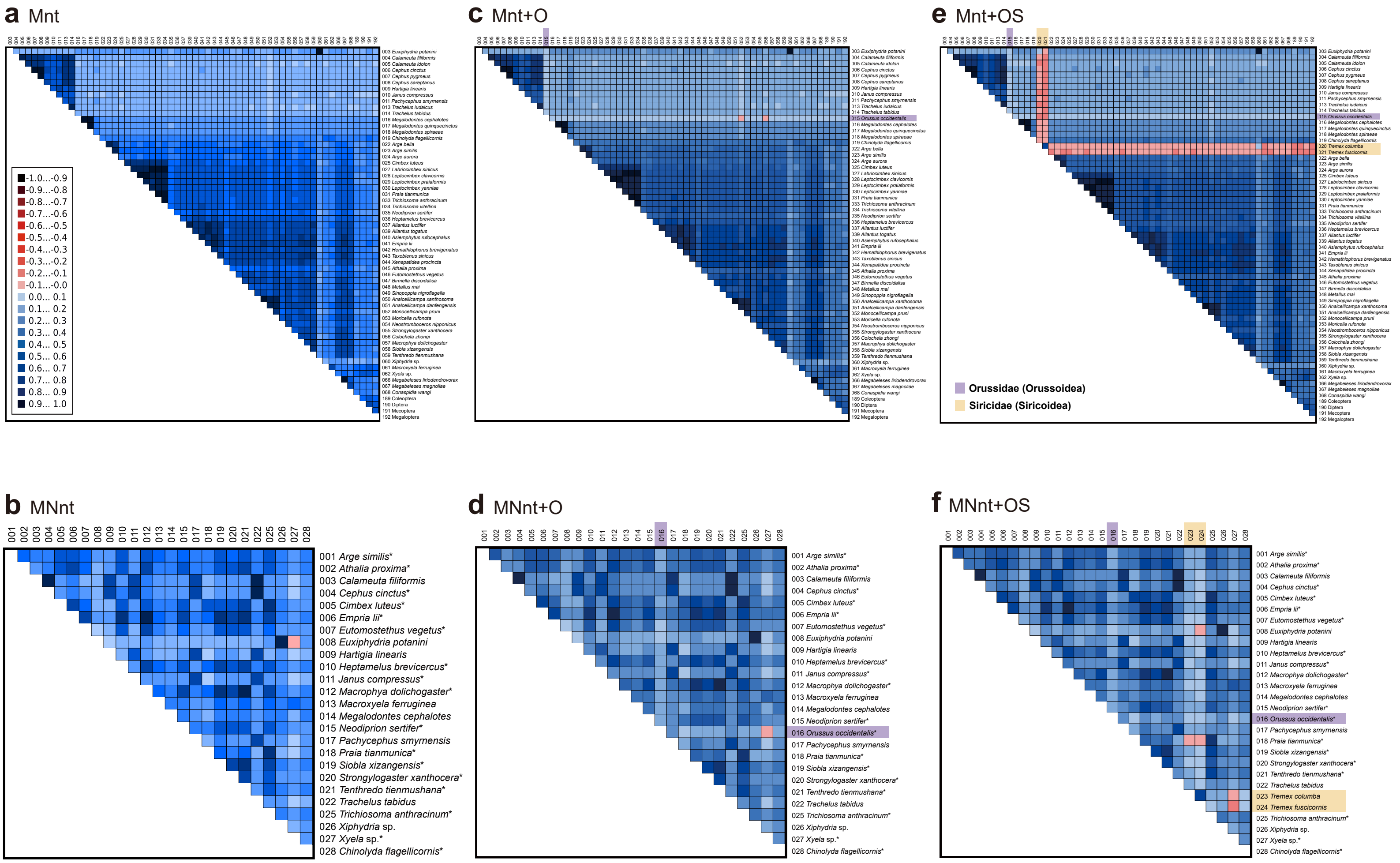

Figure S5 Model-based saturation plots for twelve matrices constructed in this study based on nucleotide and amino acid sequence alignment sets. Higher R2 values show that data are less saturated.

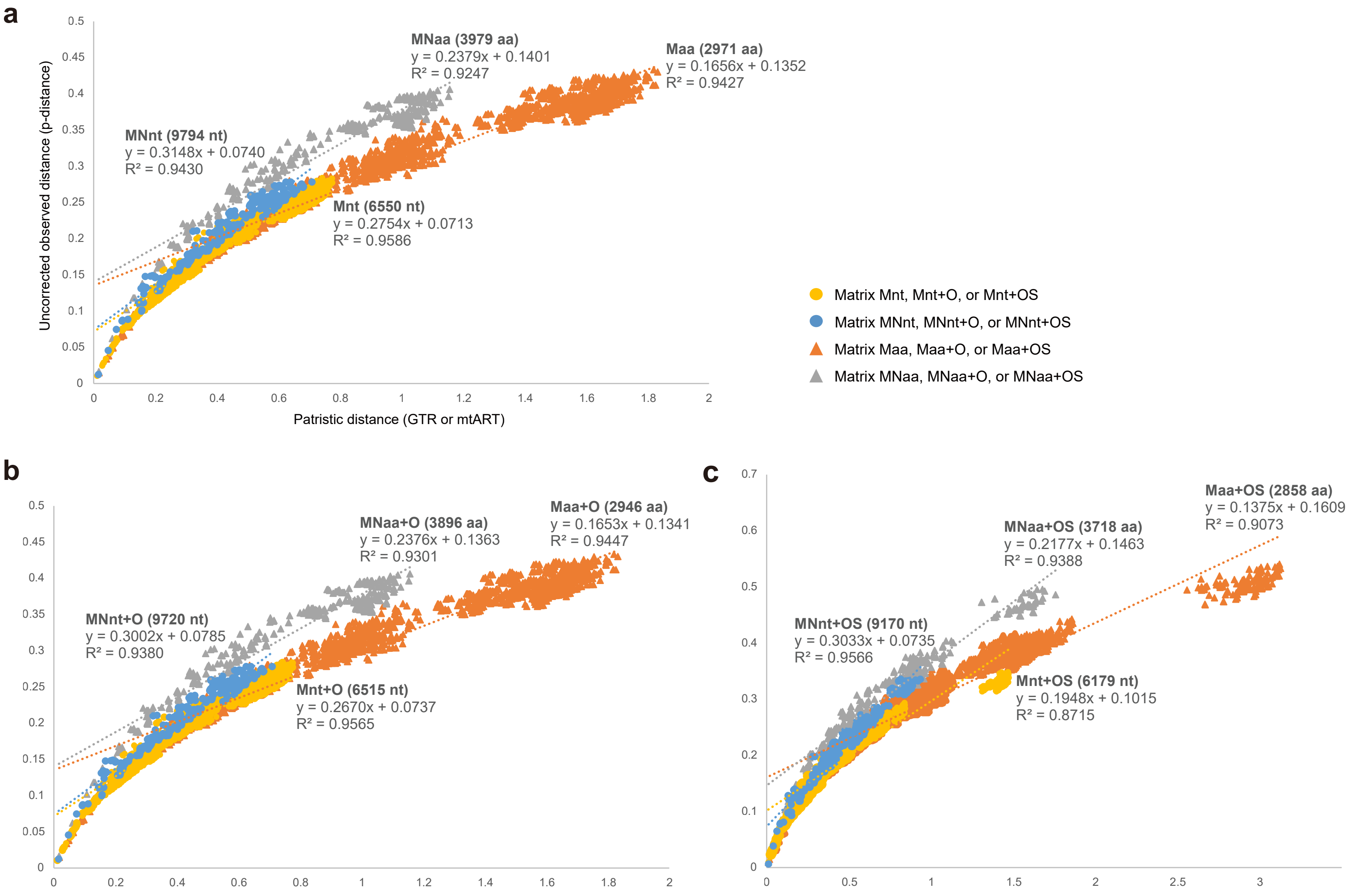

Figure S6 ML tree (IQ-TREE) based on the nucleotide sequence alignment set of 13 mtPCGs of Matrix Mnt.

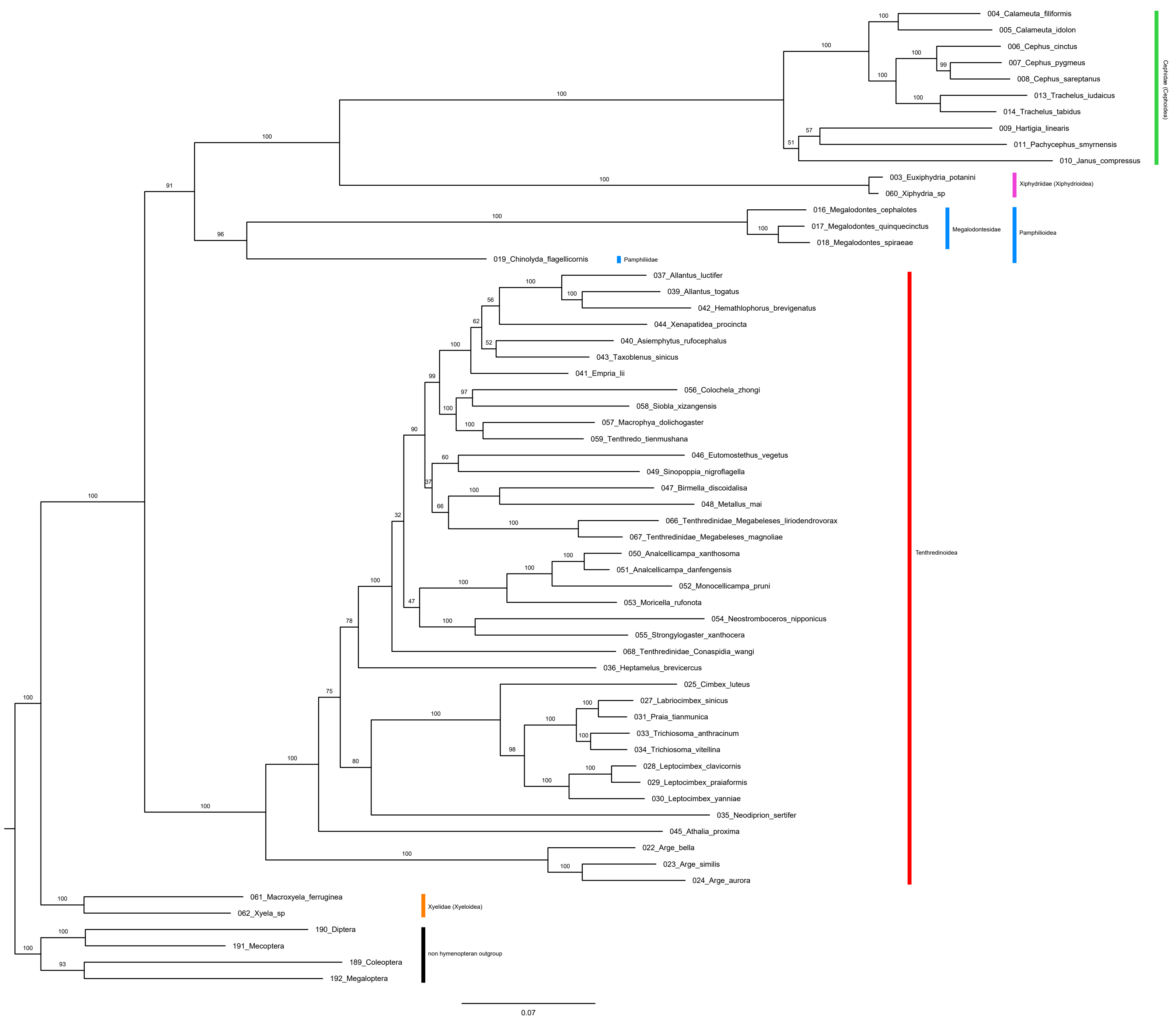

Figure S7 ML tree (RAxML) based on the nucleotide sequence alignment set of 13 mtPCGs of Matrix Mnt.

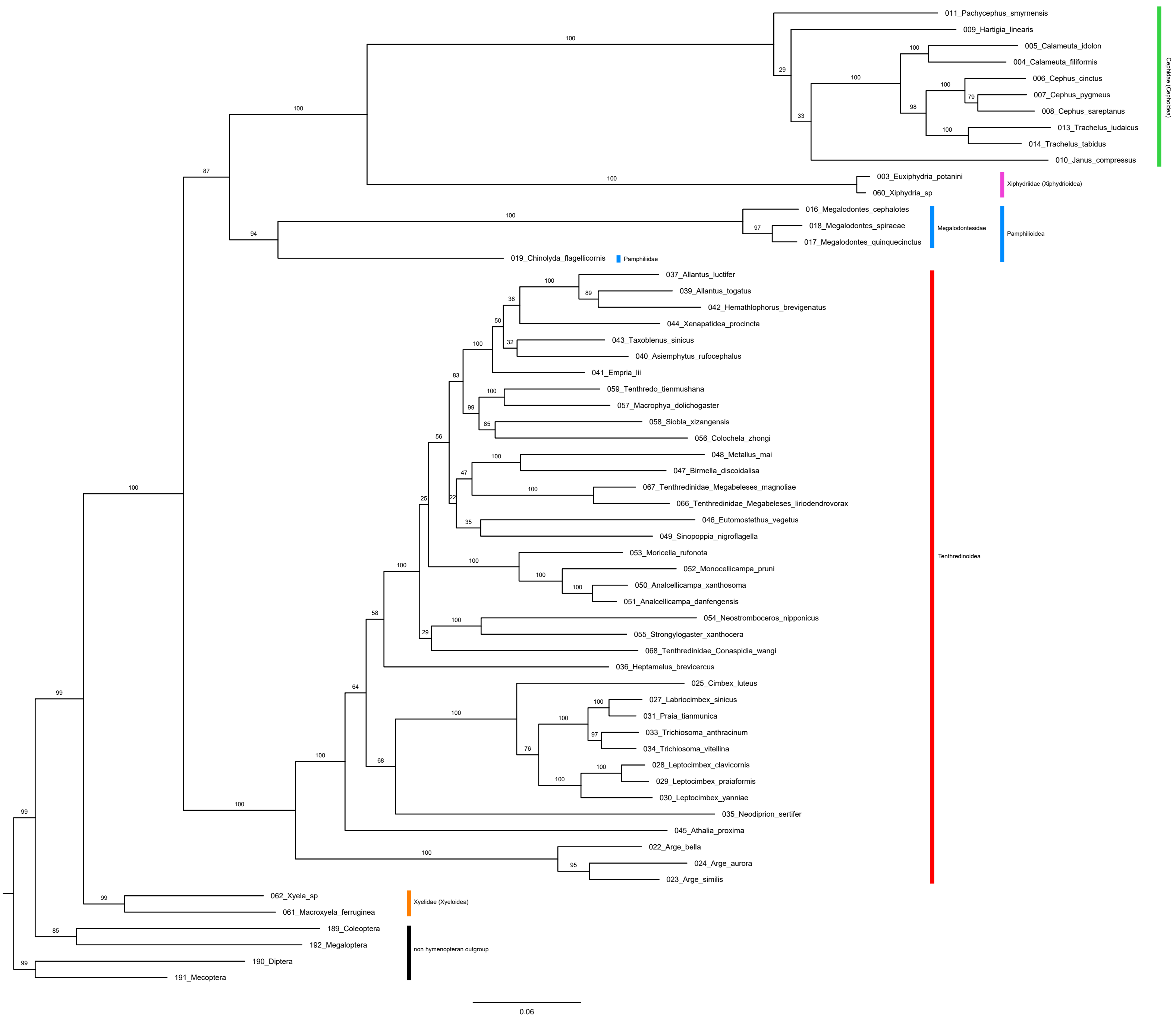

Figure S8 BI tree (MrBayes) based on the nucleotide sequence alignment set of 13 mtPCGs of Matrix Mnt.

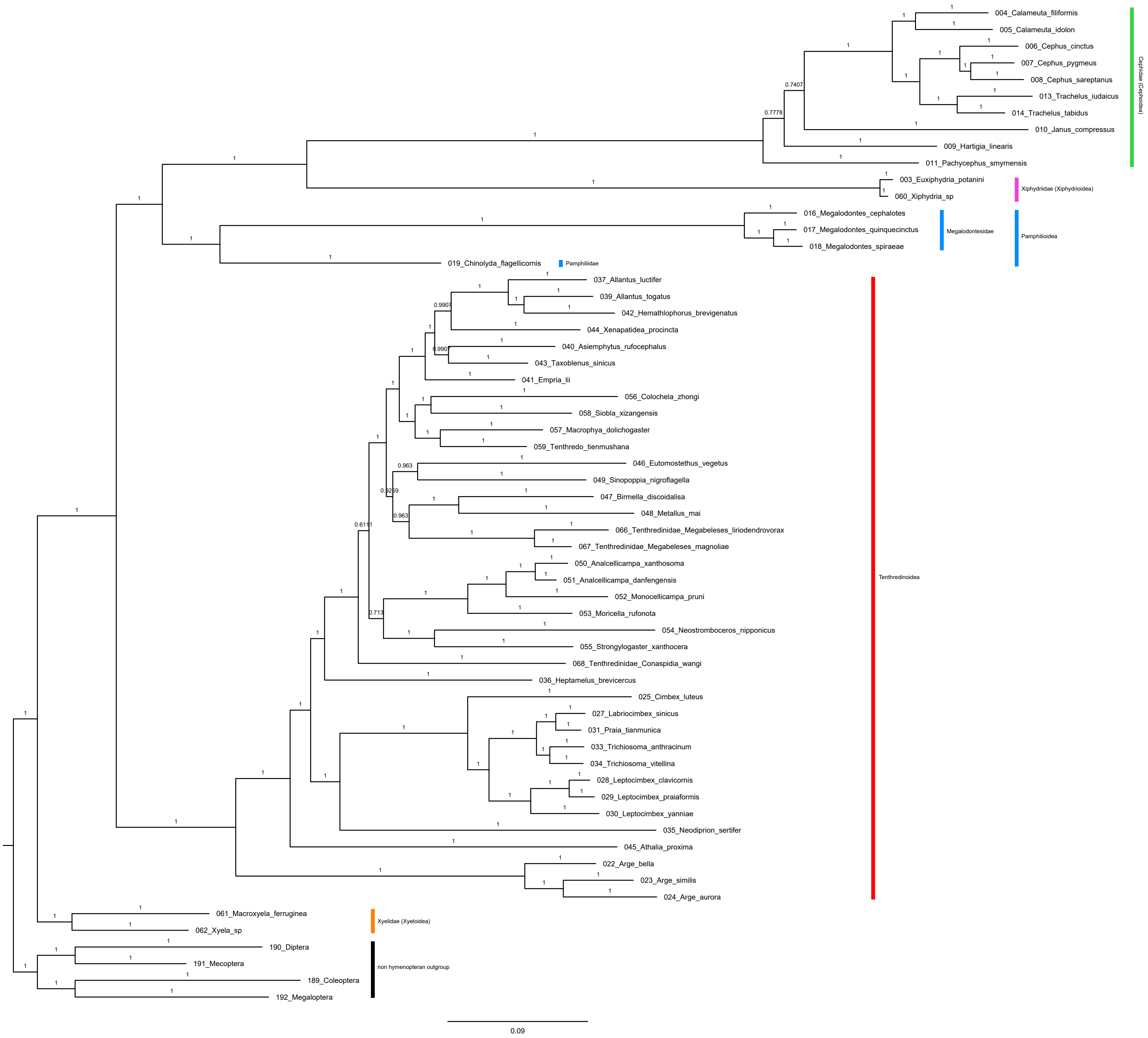

Figure S9 ML tree (IQ-TREE) based on the amino acid sequence alignment set of 13 mtPCGs of Matrix Maa.

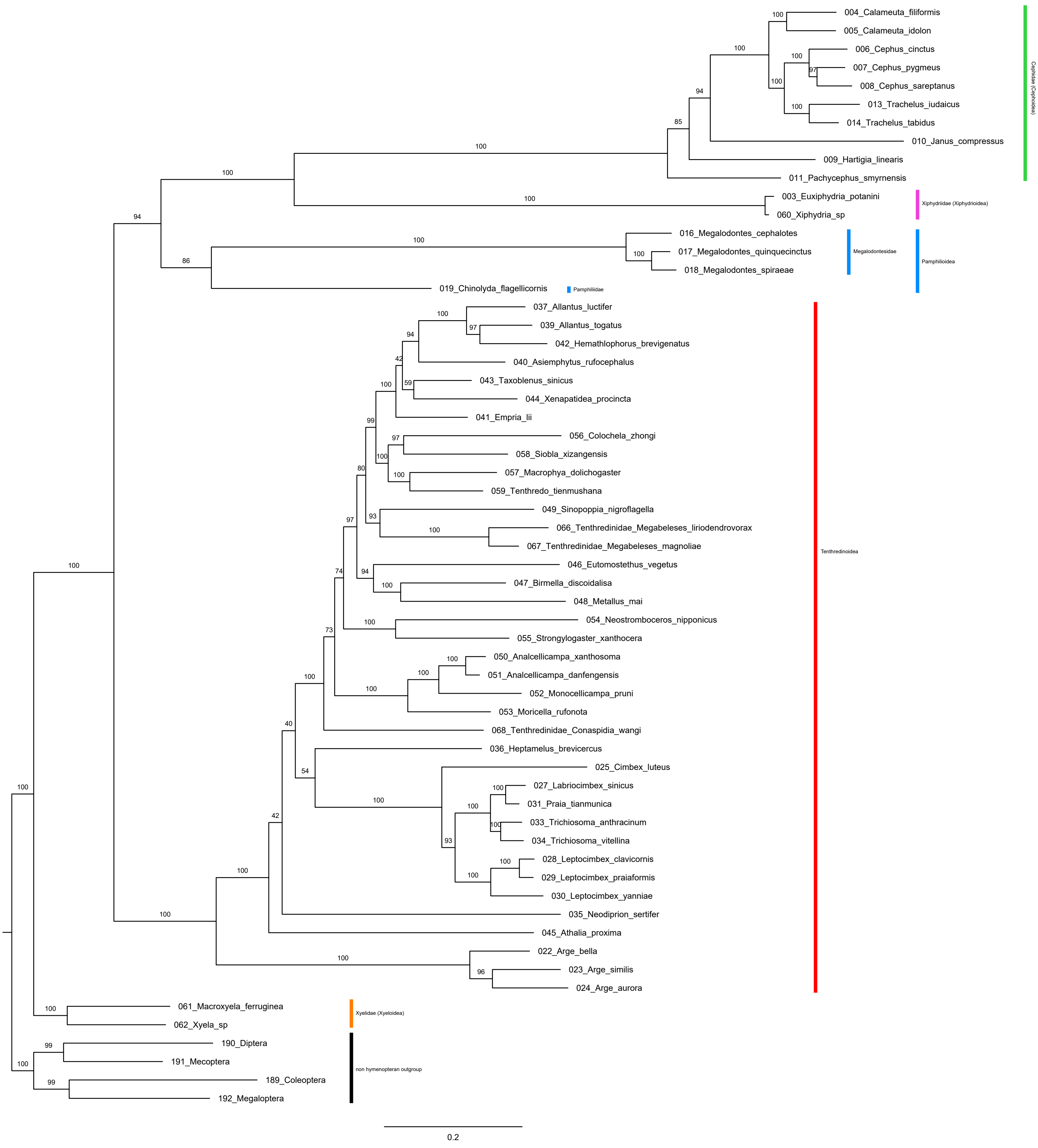

Figure S10 ML tree (RAxML) based on the amino acid sequence alignment set of 13 mtPCGs of Matrix Maa.

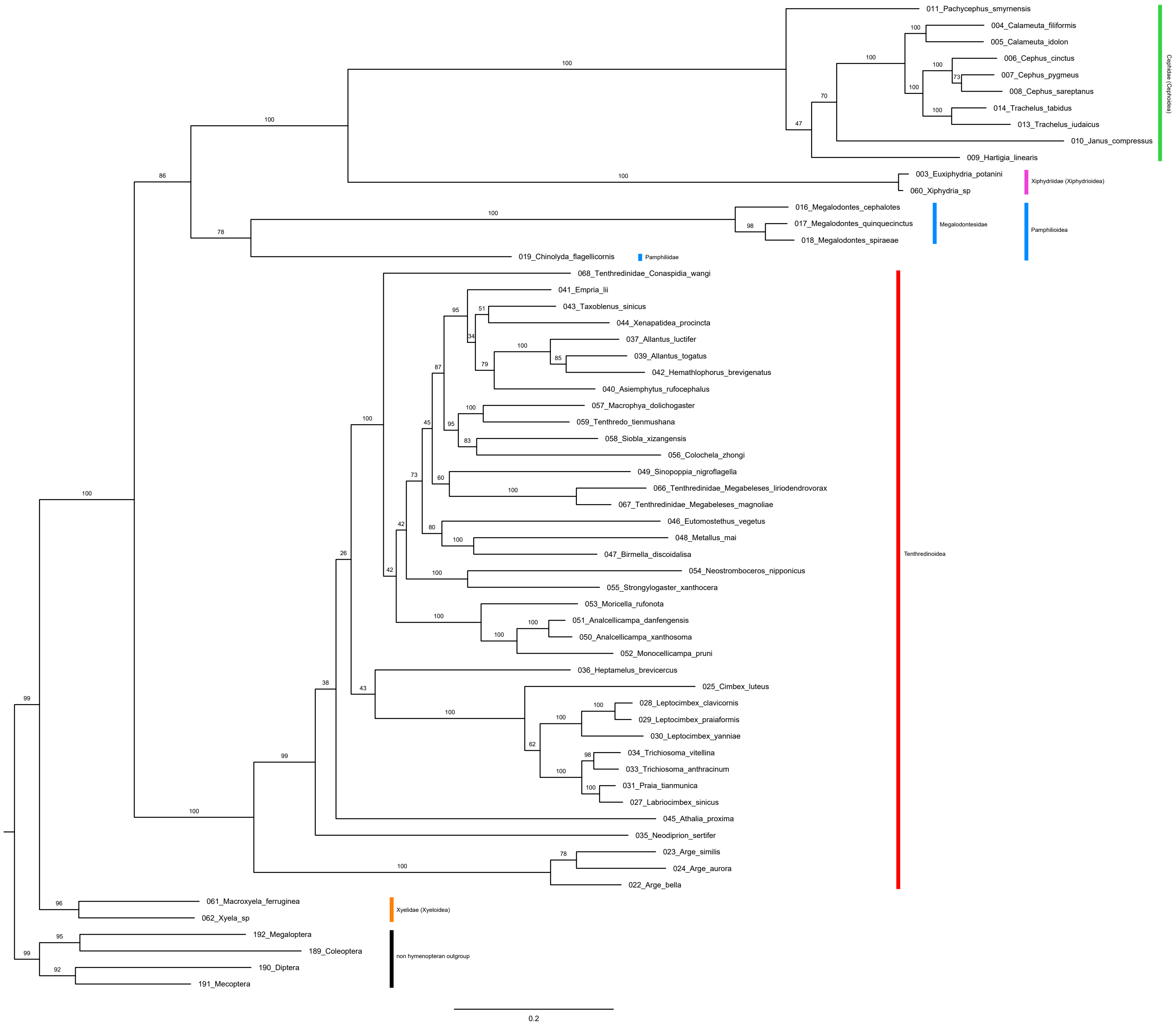

Figure S11 BI tree (MrBayes) based on the amino acid sequence alignment set of 13 mtPCGs of Matrix Maa.

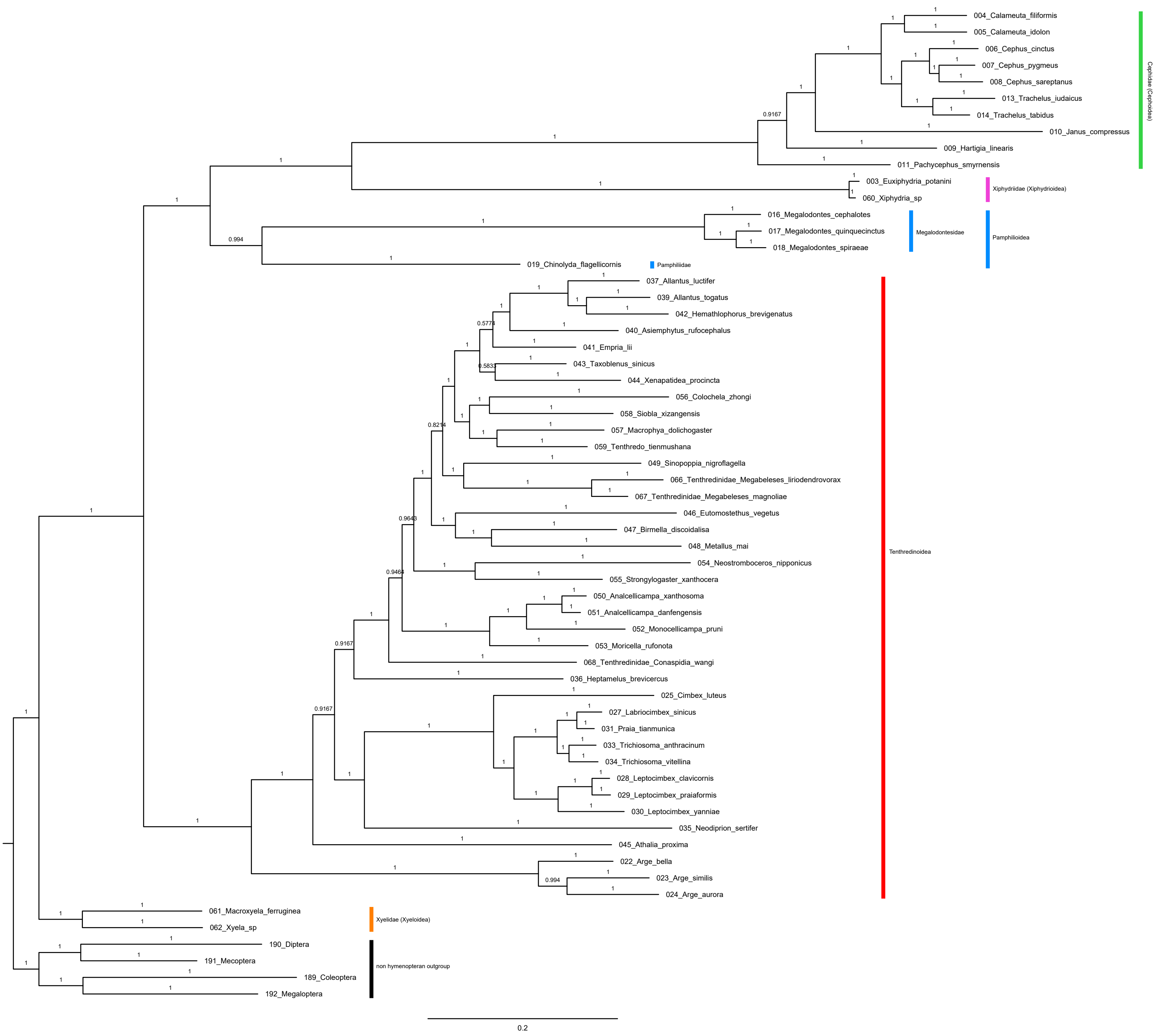

Figure S12 ML tree (IQ-TREE) based on the nucleotide sequence alignment set of 13 mtPCGs plus 8 nDNA of Matrix MNnt.

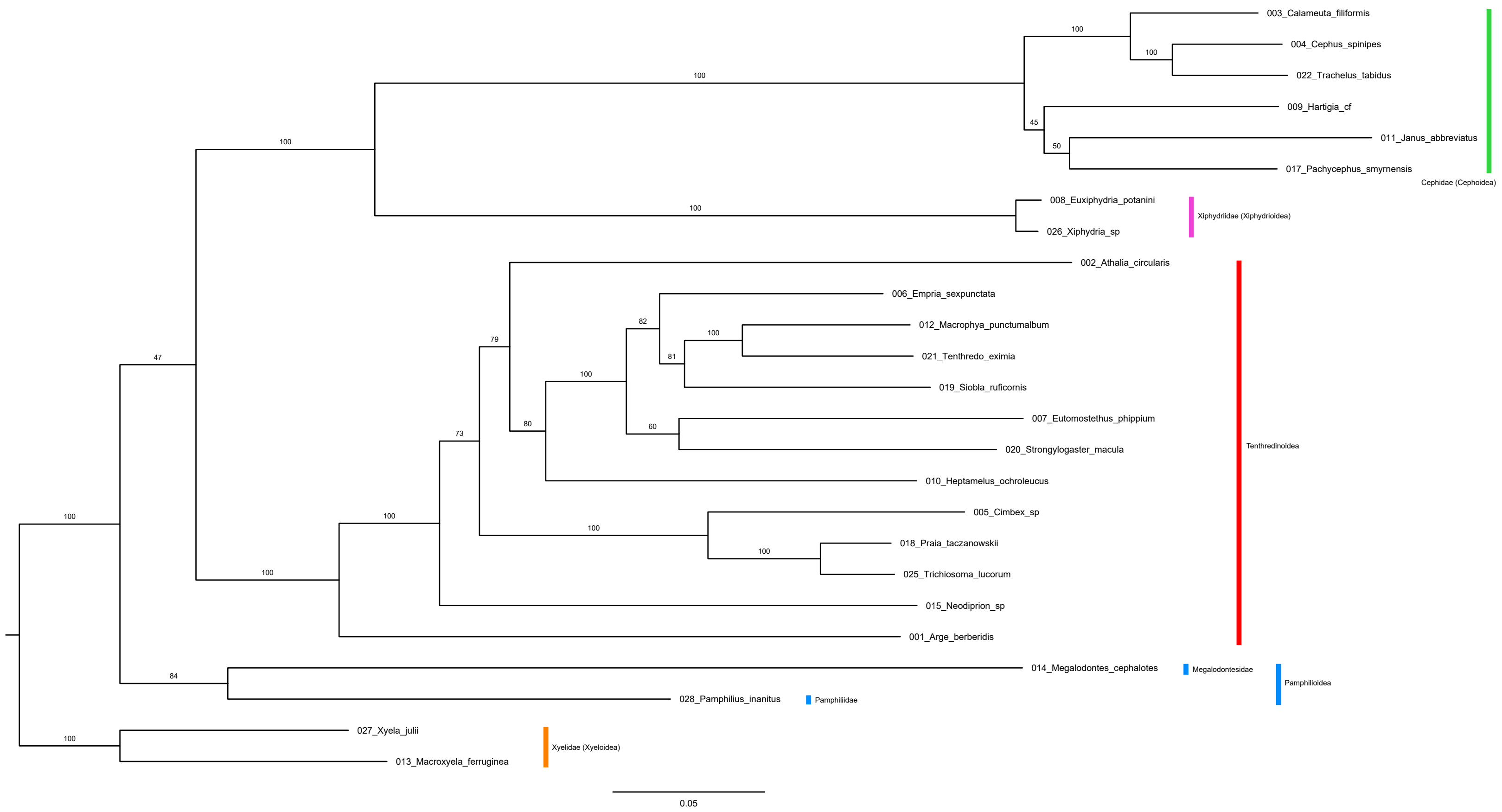

Figure S13 ML tree (RAxML) based on the nucleotide sequence alignment set of 13 mtPCGs plus 8 nDNA of Matrix MNnt.

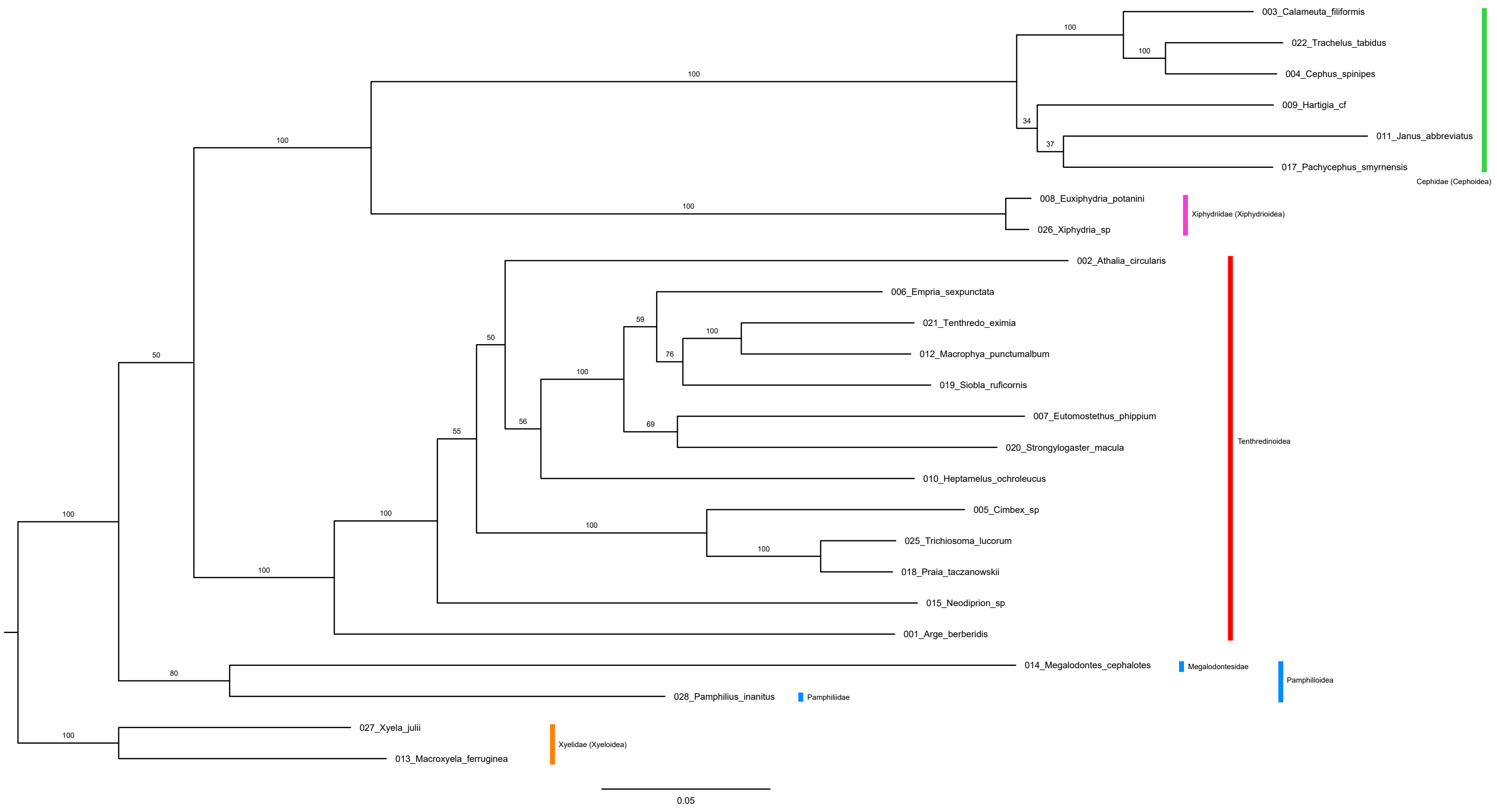

Figure S14 BI tree (MrBayes) based on the nucleotide sequence alignment set of 13 mtPCGs plus 8 nDNA of Matrix MNnt.

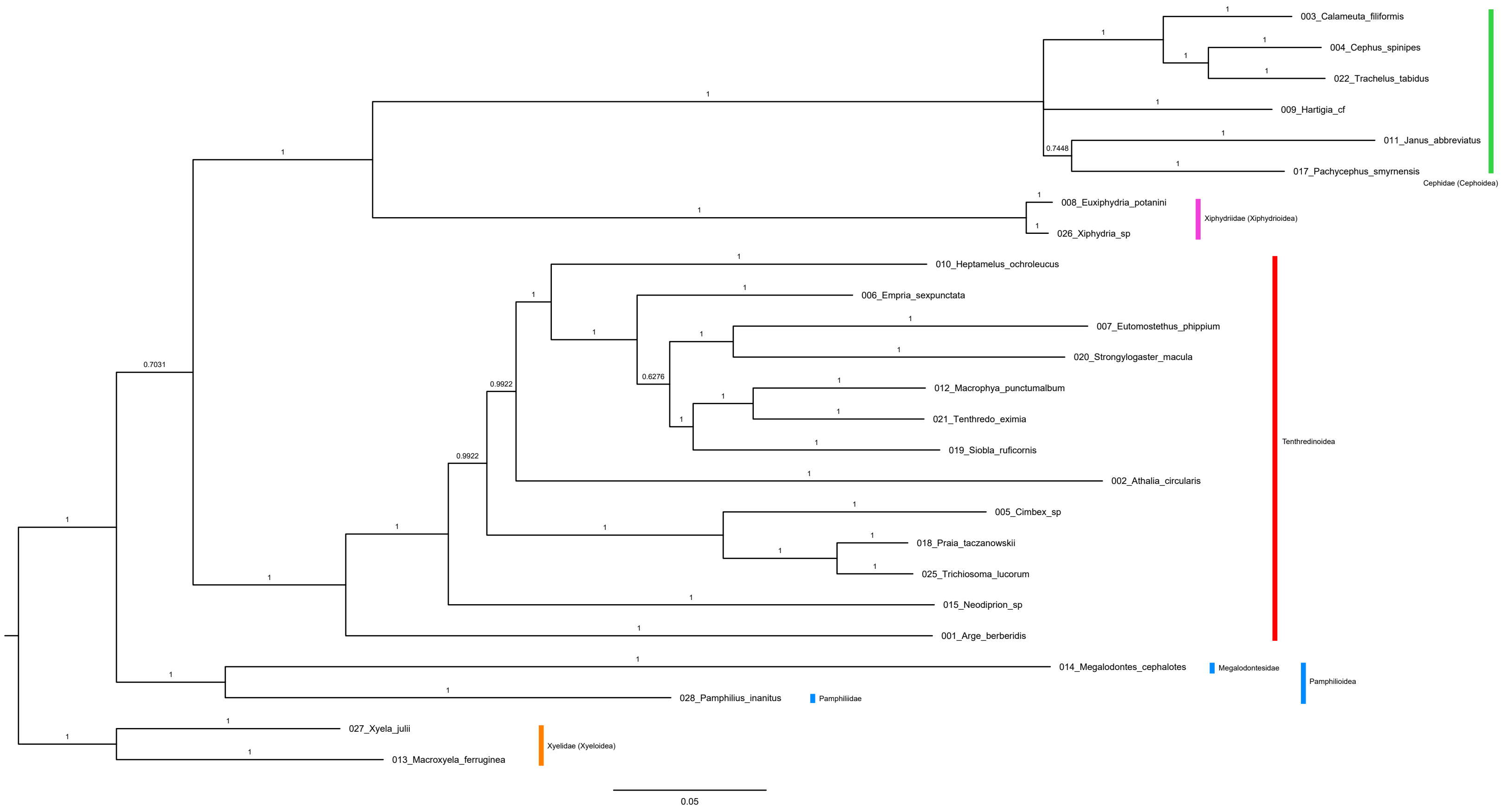

Figure S15 ML tree (IQ-TREE) based on the amino acid sequence alignment set of 13 mtPCGs plus 8 nDNA of Matrix MNaa.

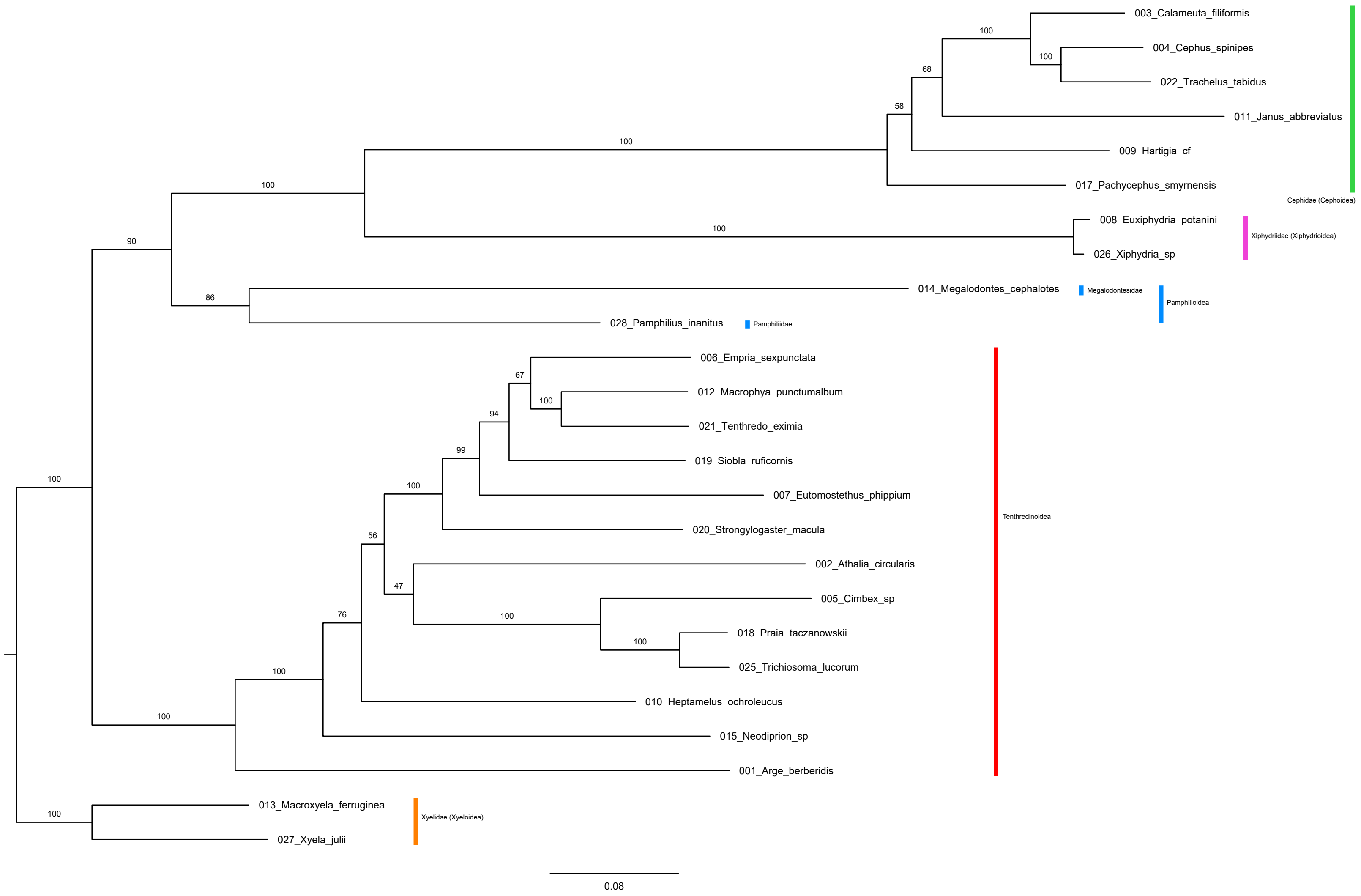

Figure S16 ML tree (RAxML) based on the amino acid sequence alignment set of 13 mtPCGs plus 8 nDNA of Matrix MNaa.

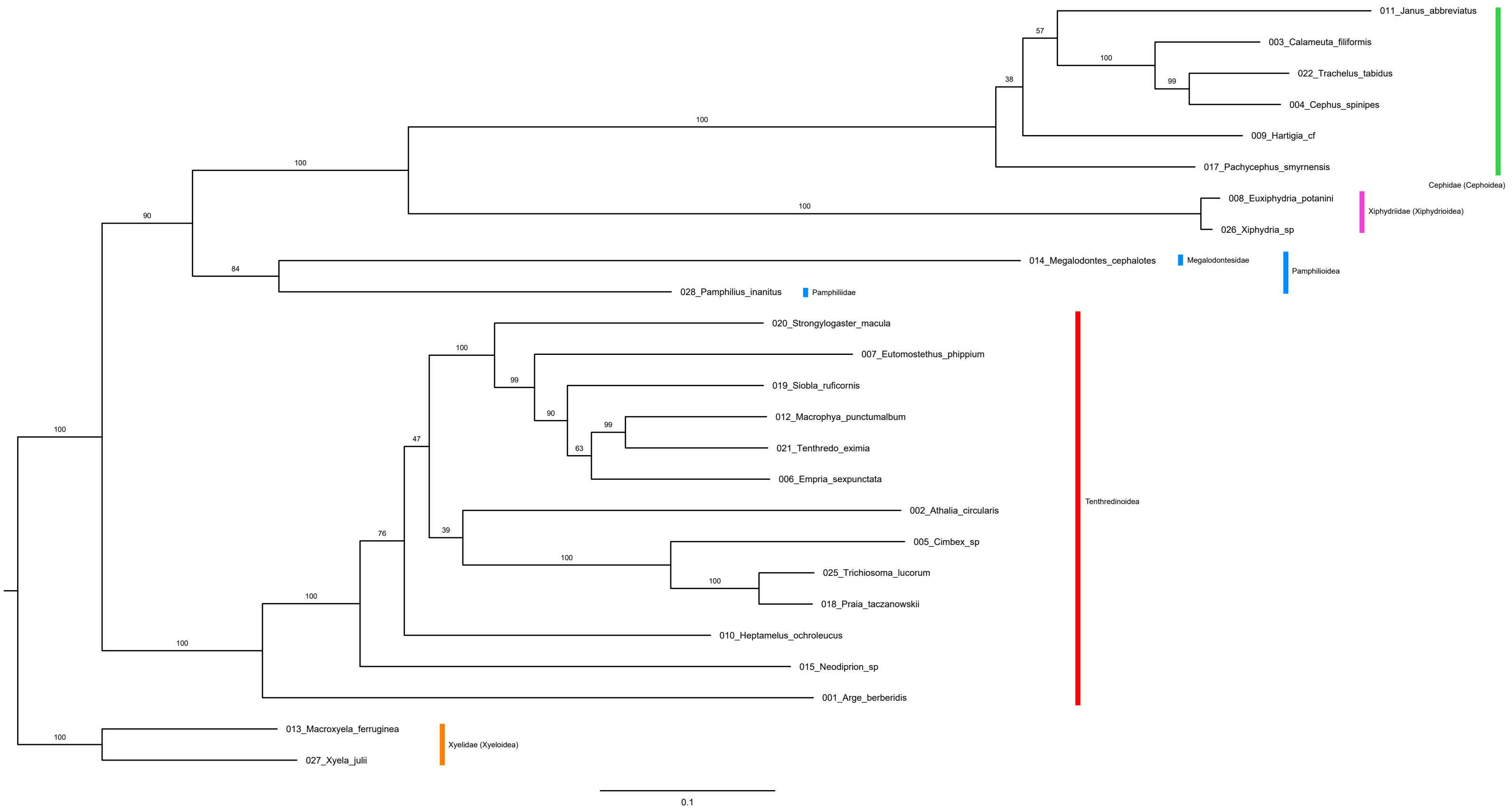

Figure S17 BI tree (MrBayes) based on the amino acid sequence alignment set of 13 mtPCGs plus 8 nDNA of Matrix MNaa.

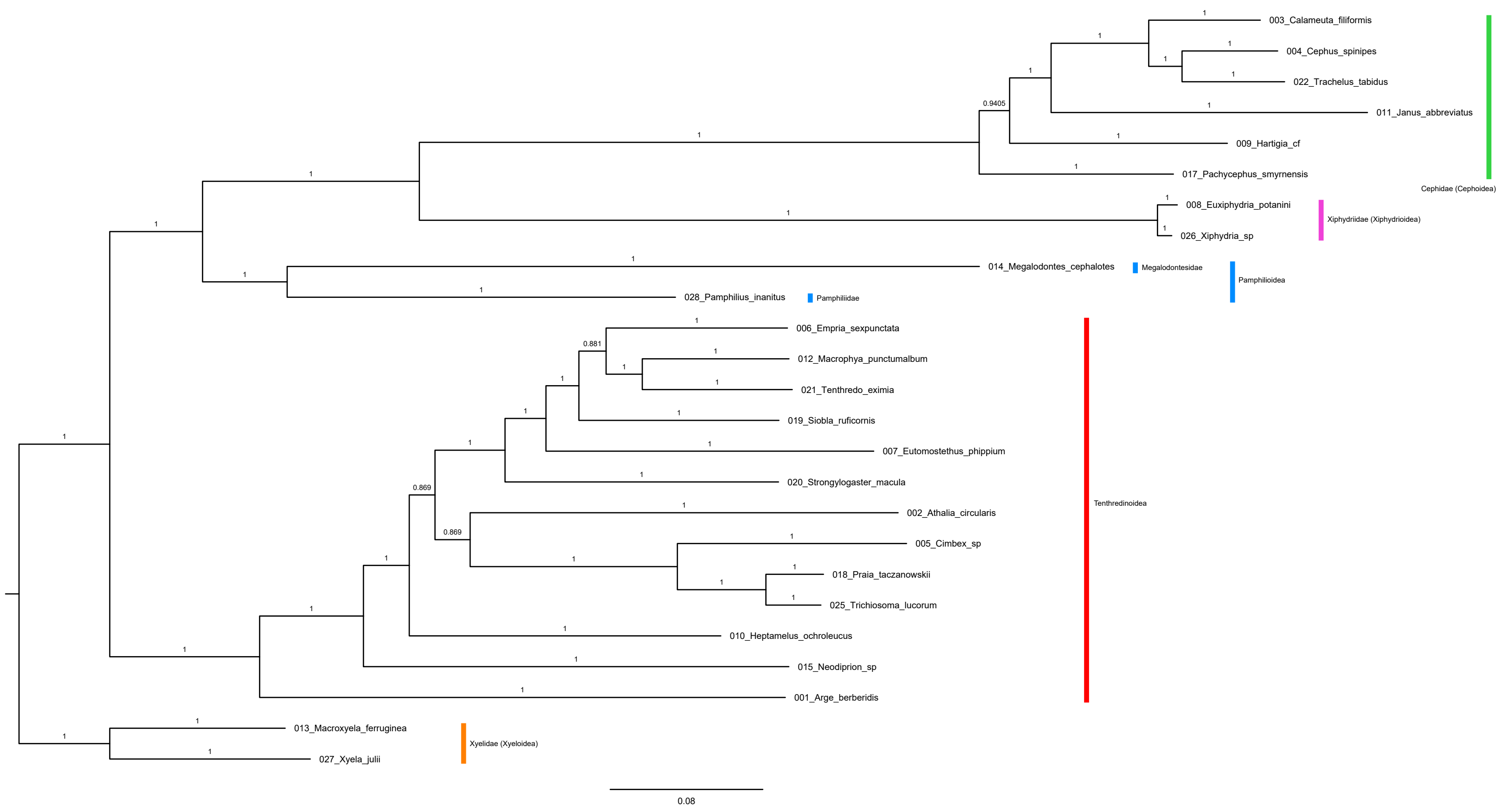

Figure S18 The ML trees showing phylogenetic positions of Orussoidea and Siricoidea within Symphyta based on the mitochondrial nucleotide sequence alignment sets (Matrices Mnt+O and Mnt+OS): (a) The phylogenetic position of Orussoidea; (b) The phylogenetic position of Siricoidea.

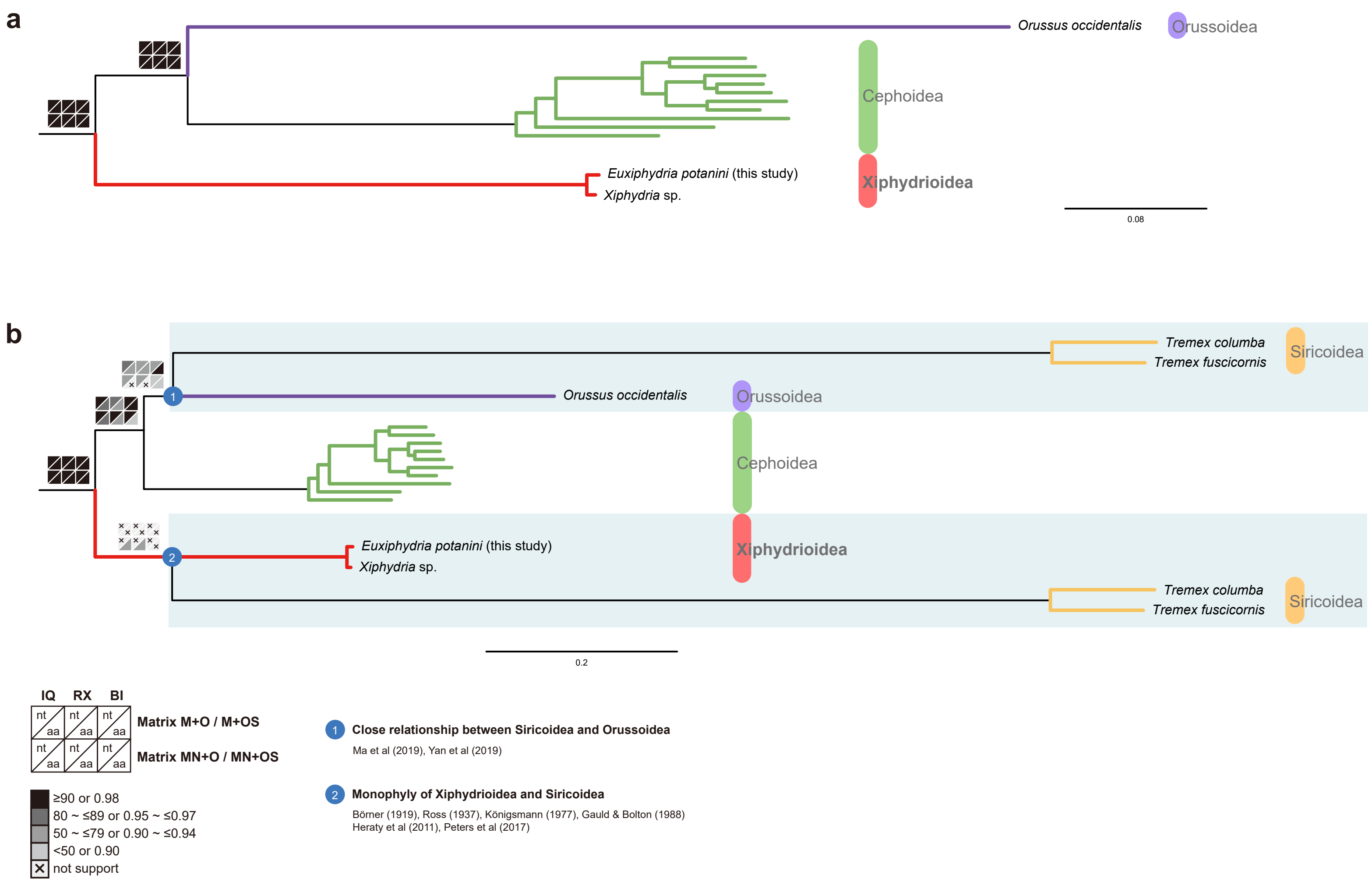

Figure S19 The phylogenetic position of Siricoidea within Symphyta based on the nucleotide sequence alignment sets (Matrices Mnt+OS and MNnt+OS) and amino acid sequence alignment sets (Matrices Maa+OS and MNaa+OS).

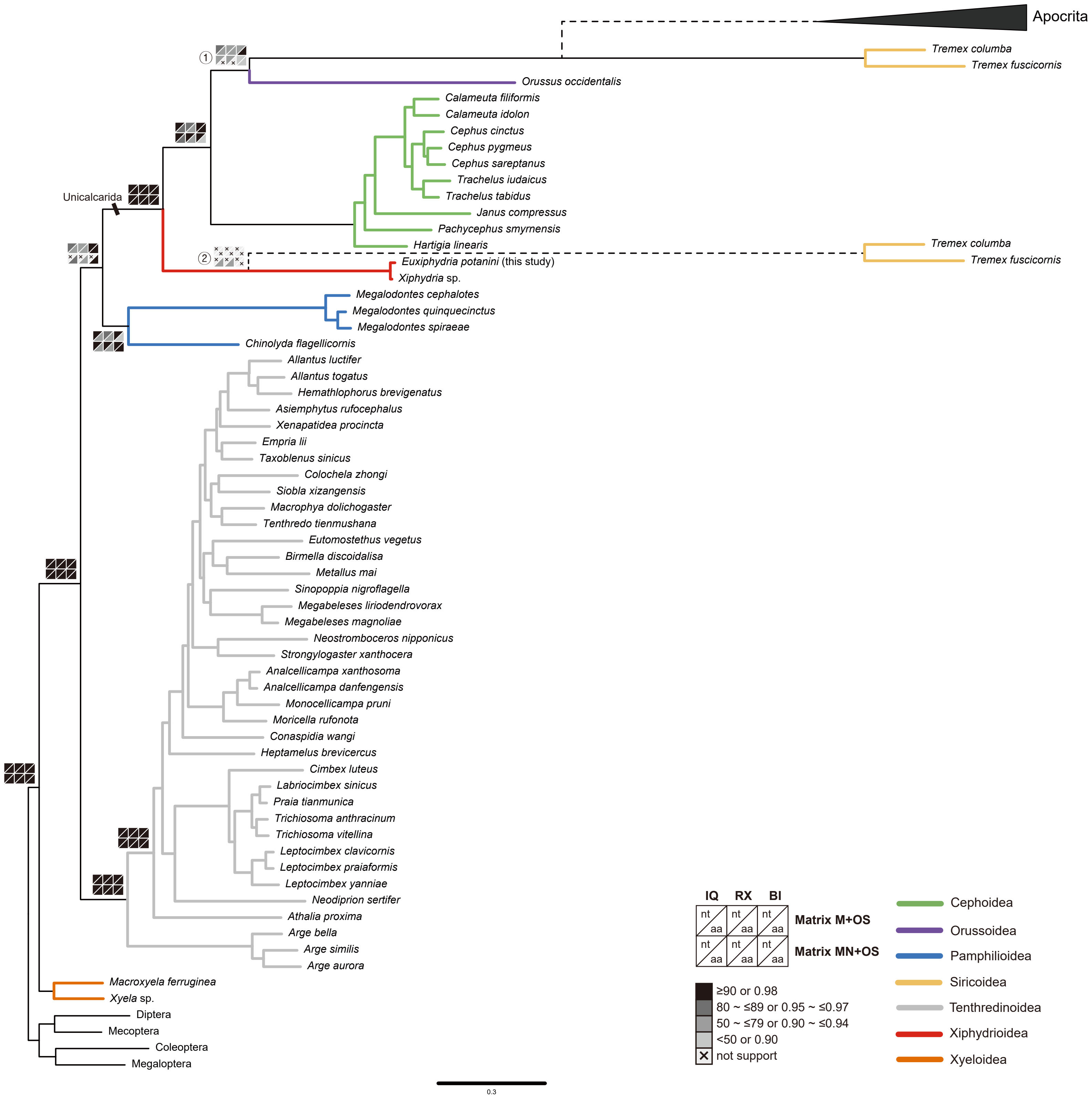

Figure S20 ML tree (IQ-TREE) based on the nucleotide sequence alignment set of 13 mtPCGs of Matrix Mnt+O.

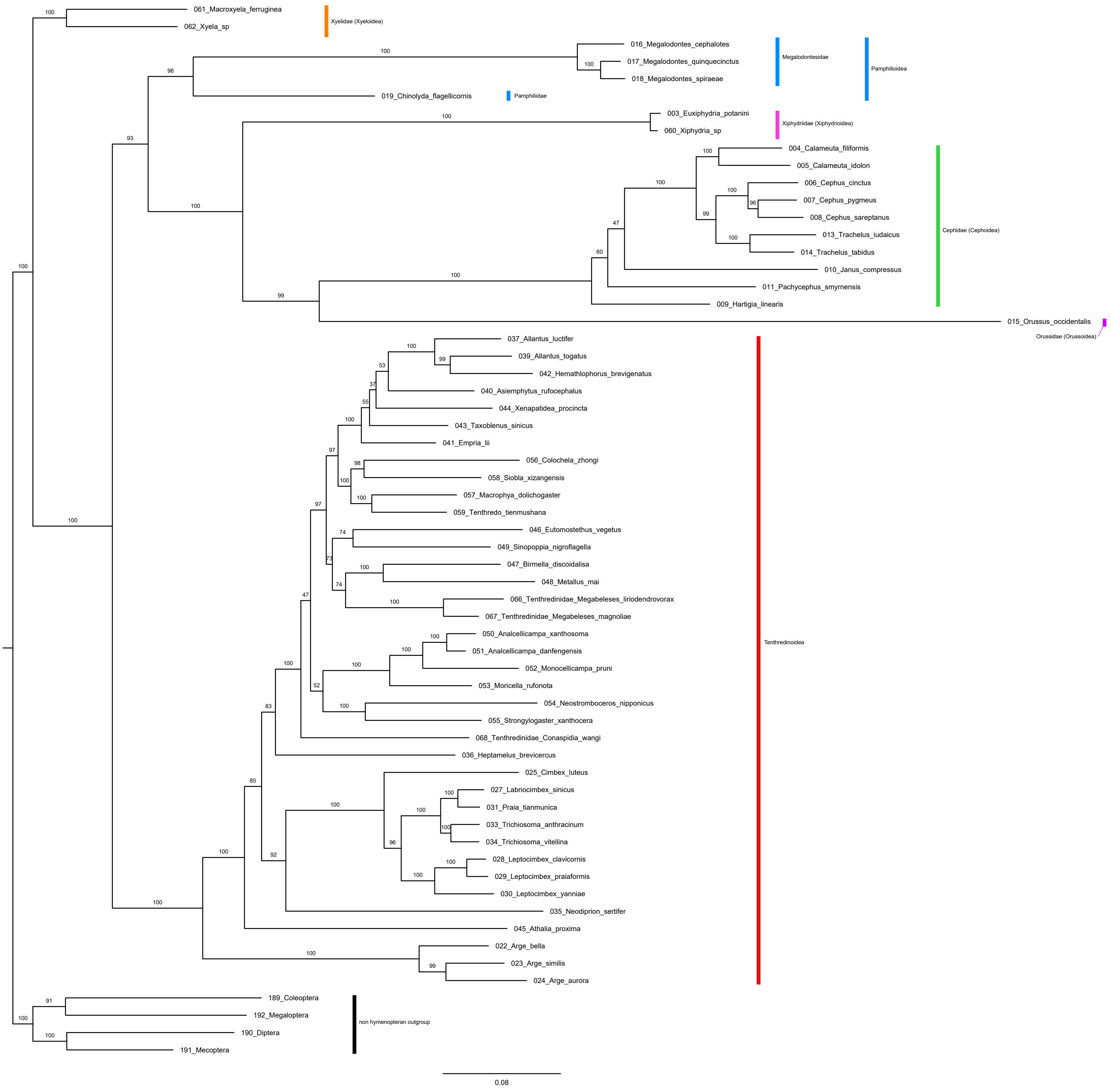

Figure S21 ML tree (RAxML) based on the nucleotide sequence alignment set of 13 mtPCGs of Matrix Mnt+O.

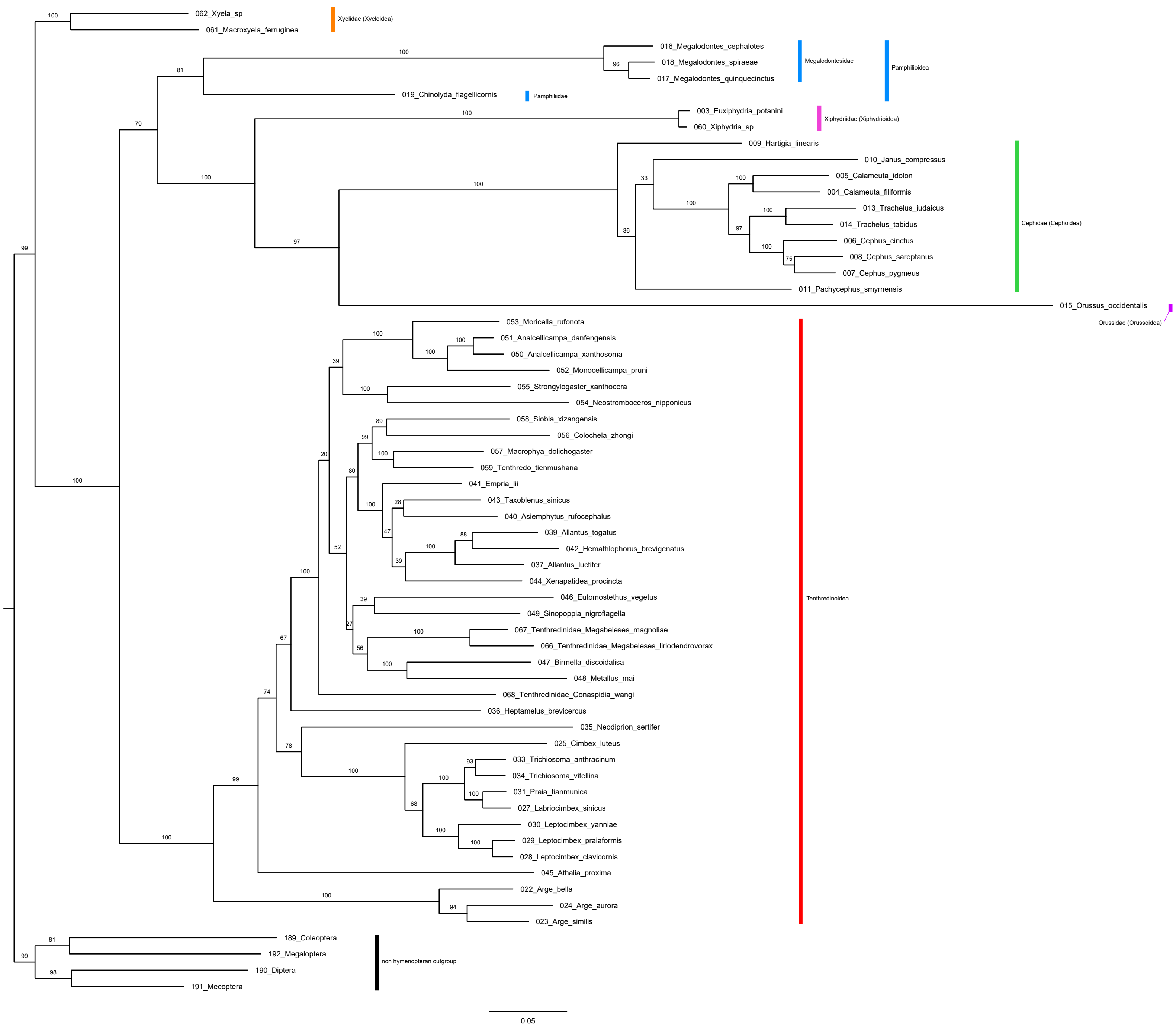

Figure S22 BI tree (MrBayes) based on the nucleotide sequence alignment set of 13 mtPCGs of Matrix Mnt+O.

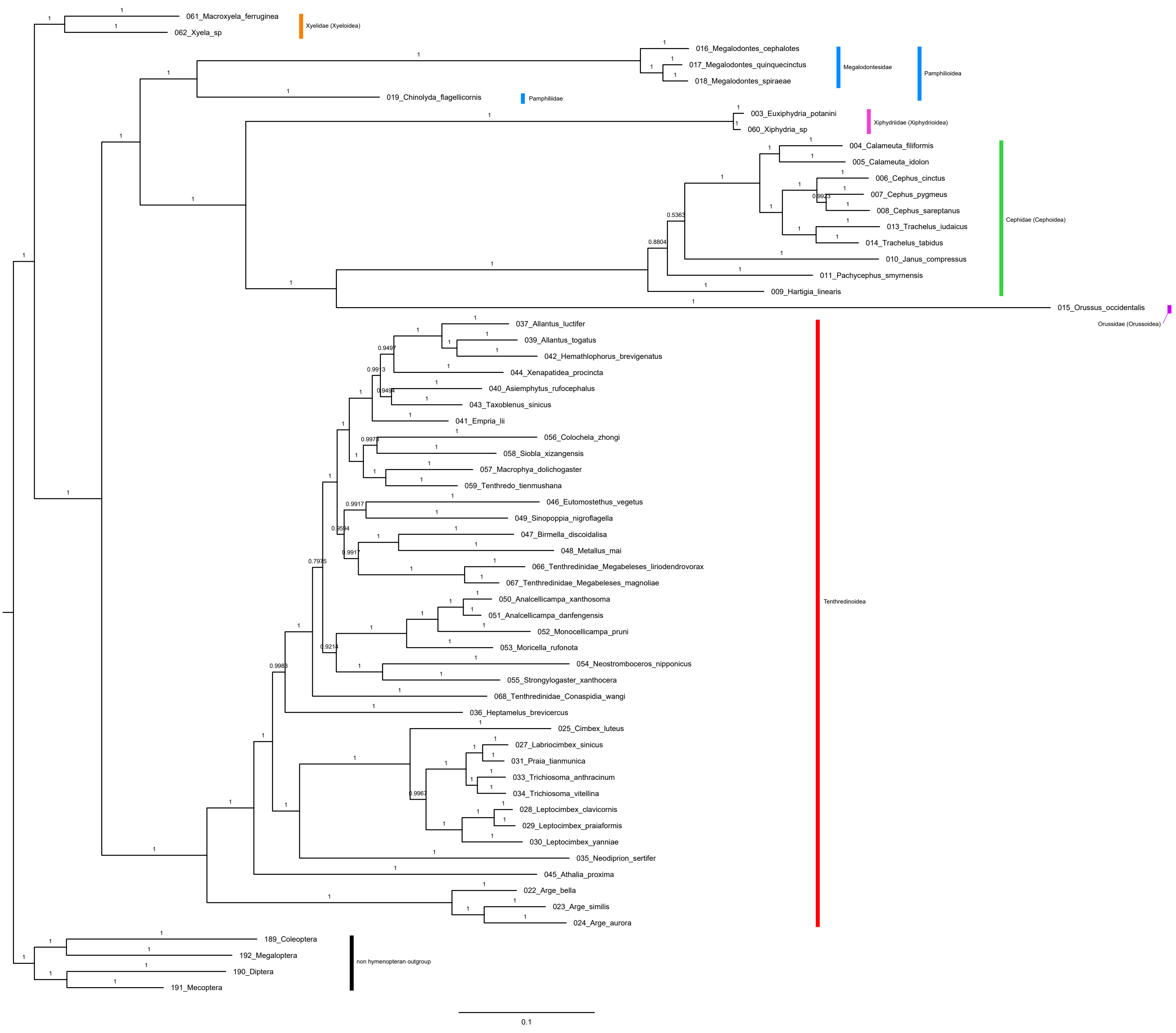

Figure S23 ML tree (IQ-TREE) based on the amino acid sequence alignment set of 13 mtPCGs of Matrix Maa+O.

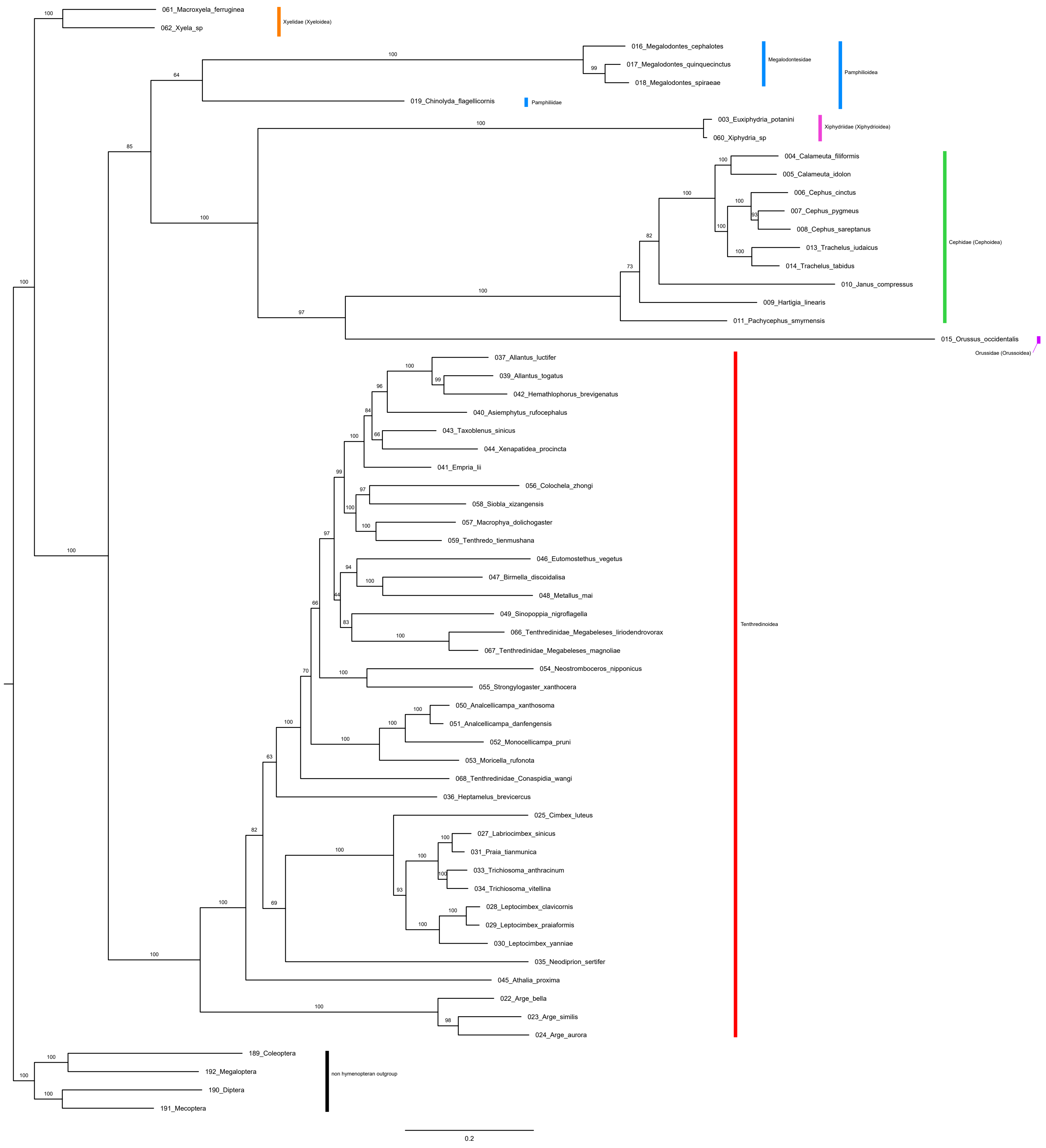

Figure S24 ML tree (RAxML) based on the amino acid sequence alignment set of 13 mtPCGs of Matrix Maa+O.

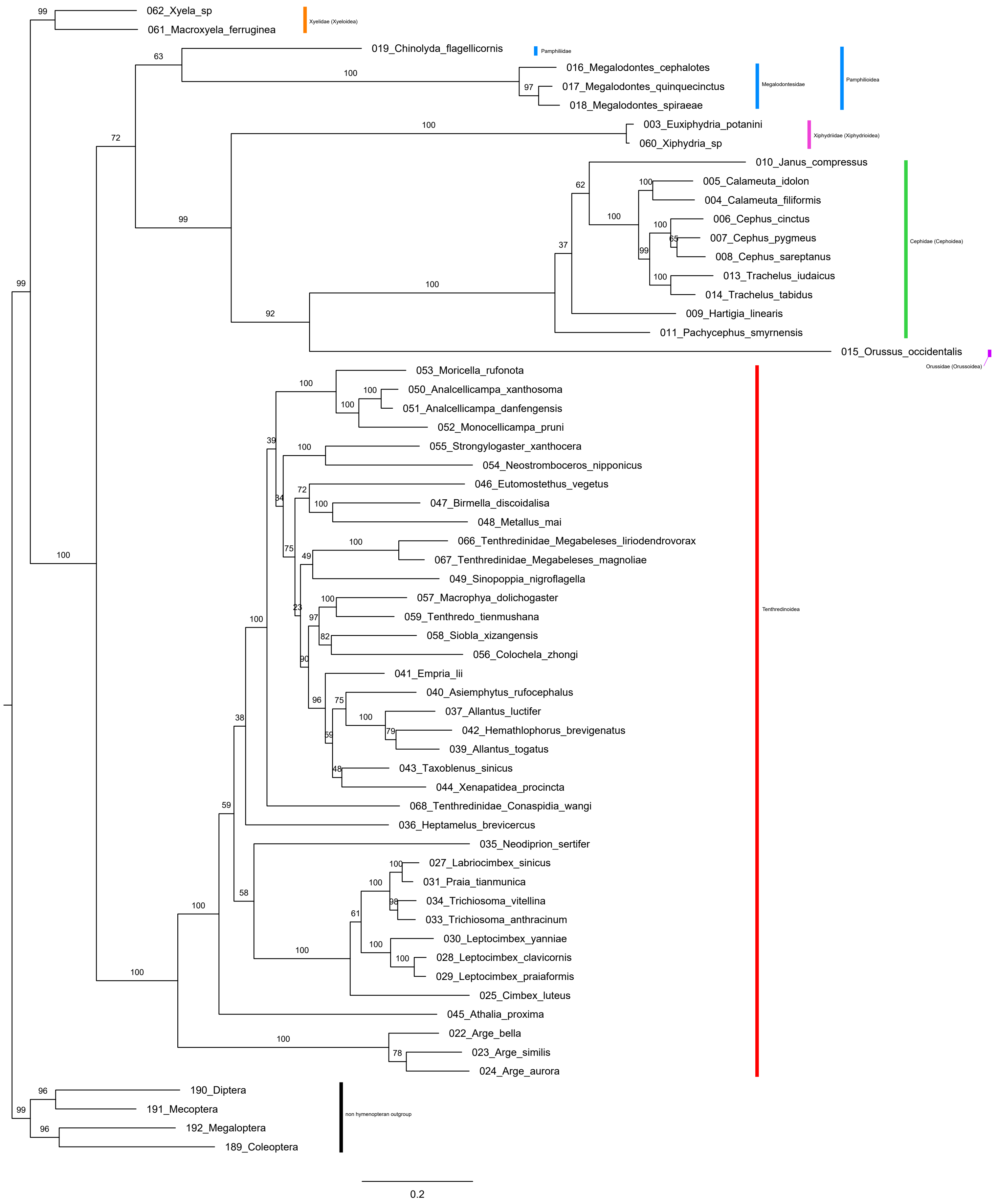

Figure S25 BI tree (MrBayes) based on the amino acid sequence alignment set of 13 mtPCGs of Matrix Maa+O.

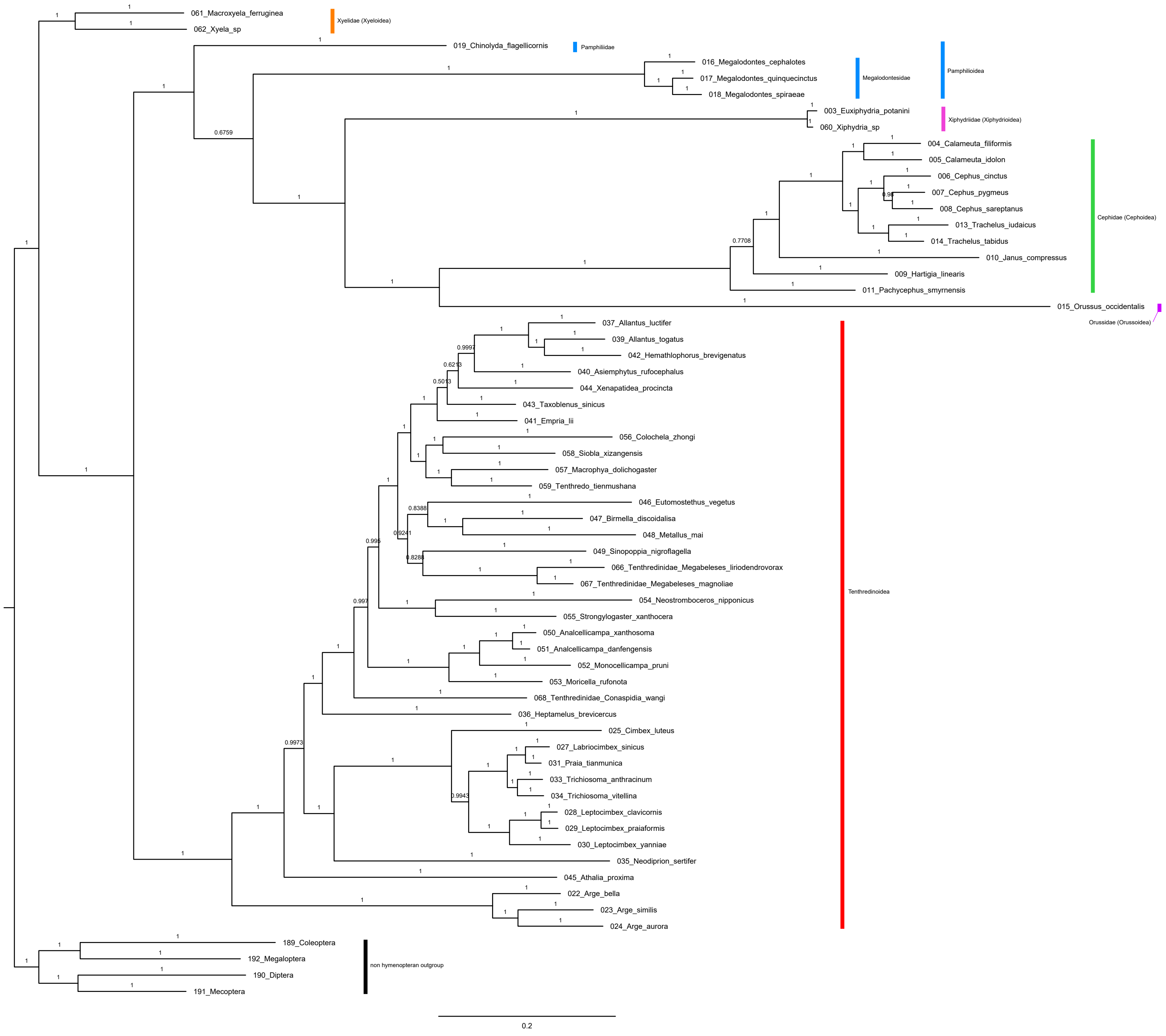

Figure S26 ML tree (IQ-TREE) based on the nucleotide sequence alignment set of 13 mtPCGs plus 8 nDNA of Matrix MNnt+O.

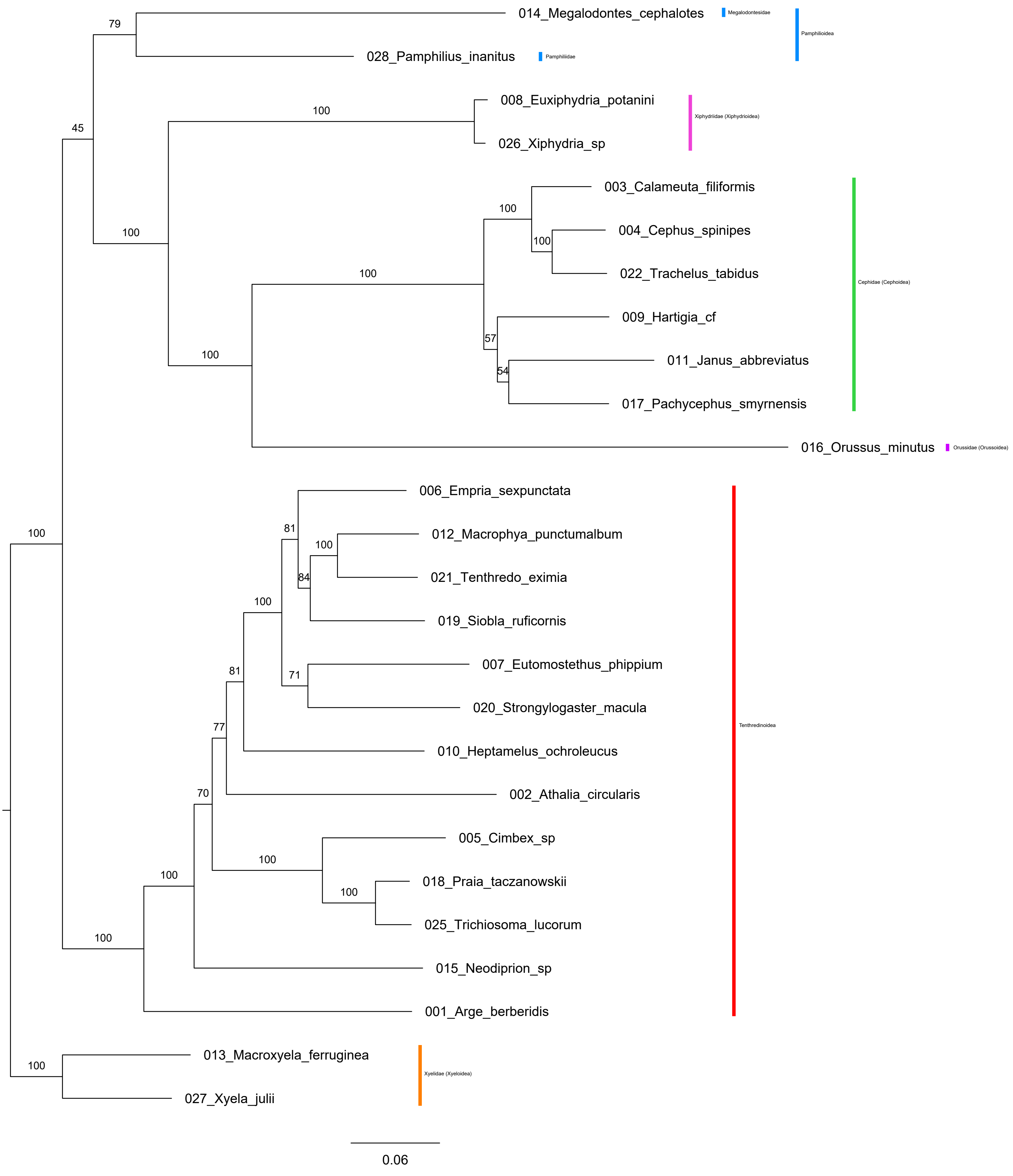

Figure S27 ML tree (RAxML) based on the nucleotide sequence alignment set of 13 mtPCGs plus 8 nDNA of Matrix MNnt+O.

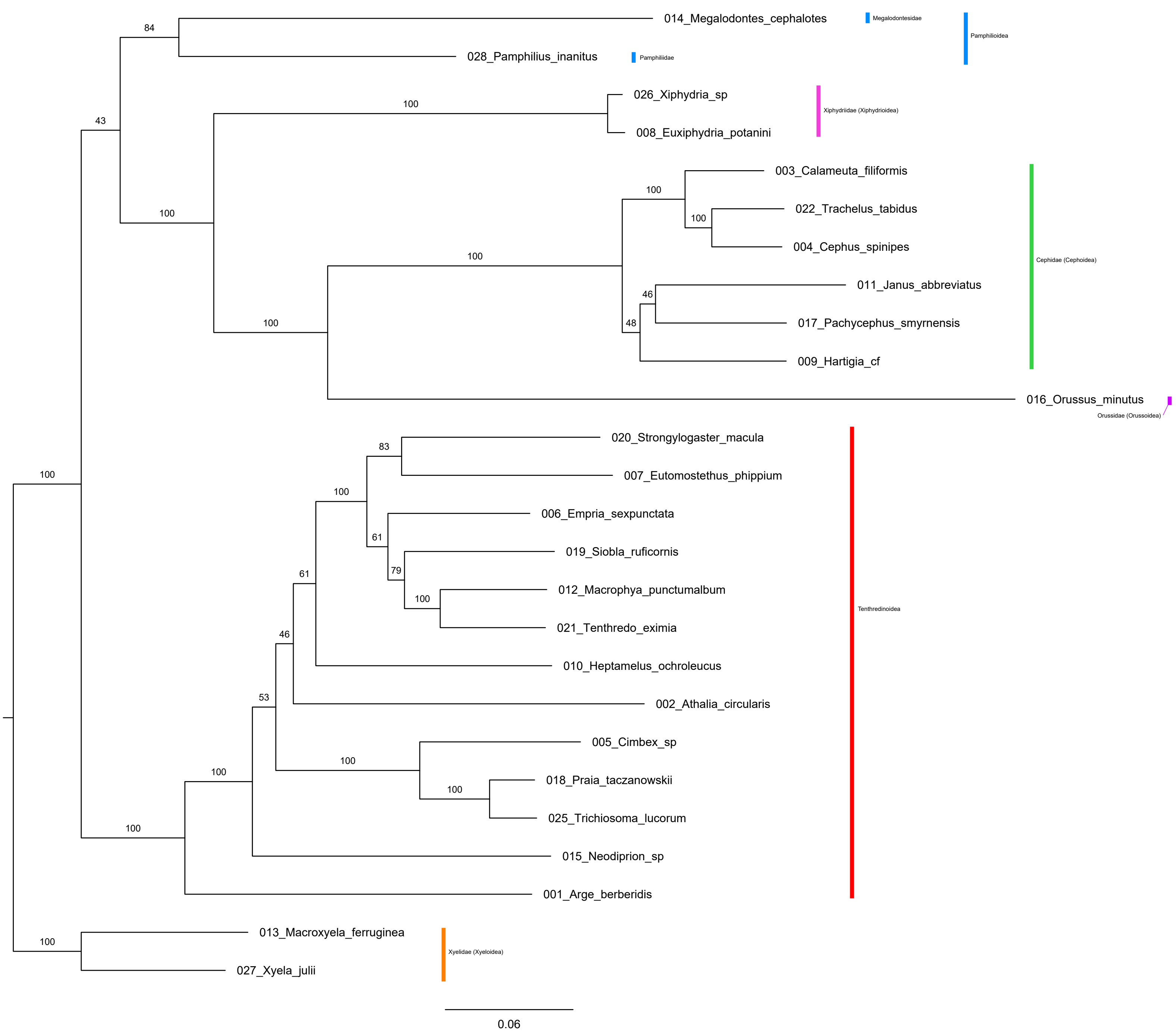

Figure S28 BI tree (MrBayes) based on the nucleotide sequence alignment set of 13 mtPCGs plus 8 nDNA of Matrix MNnt+O.

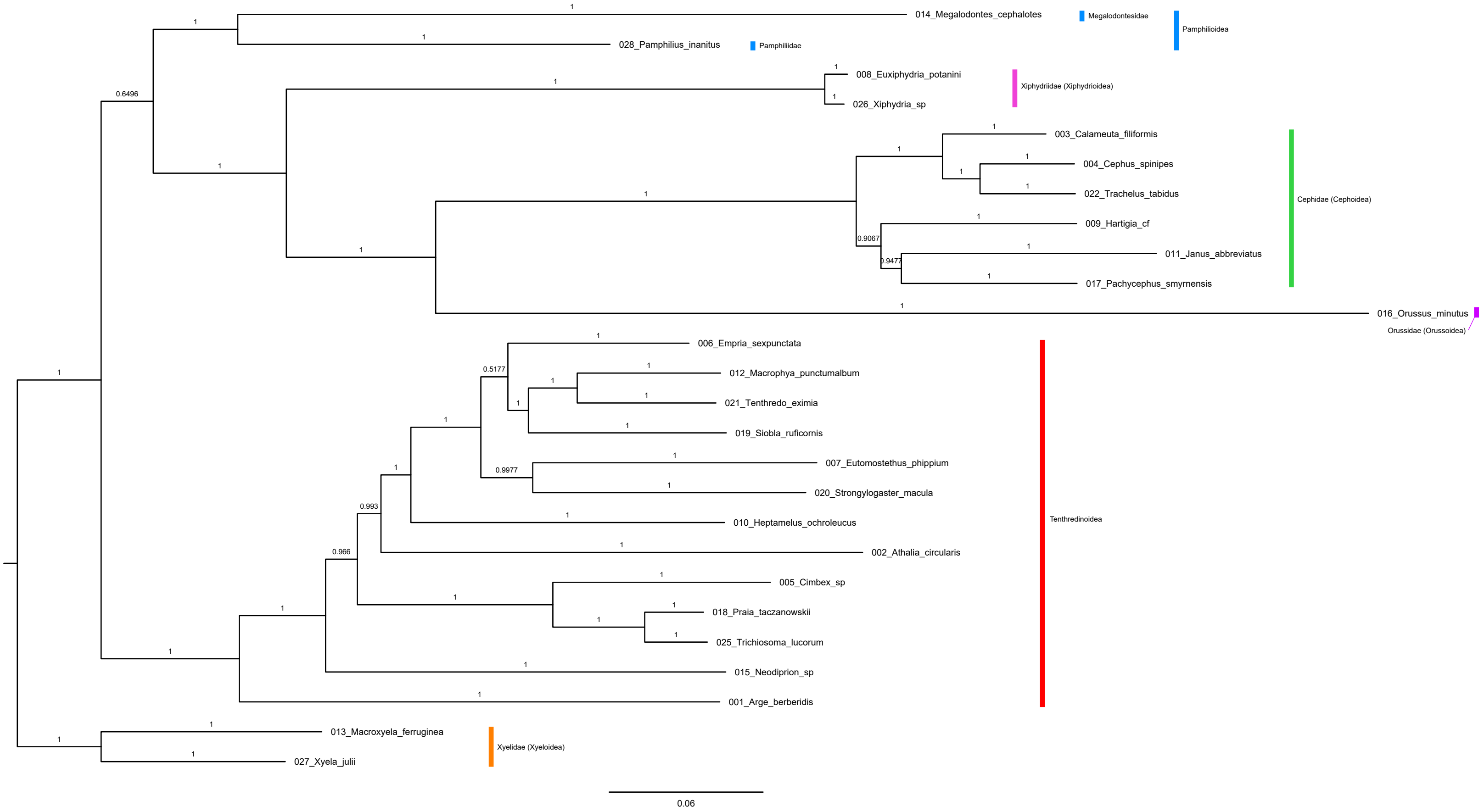

Figure S29 ML tree (IQ-TREE) based on the amino acid sequence alignment set of 13 mtPCGs plus 8 nDNA of Matrix MNaa+O.

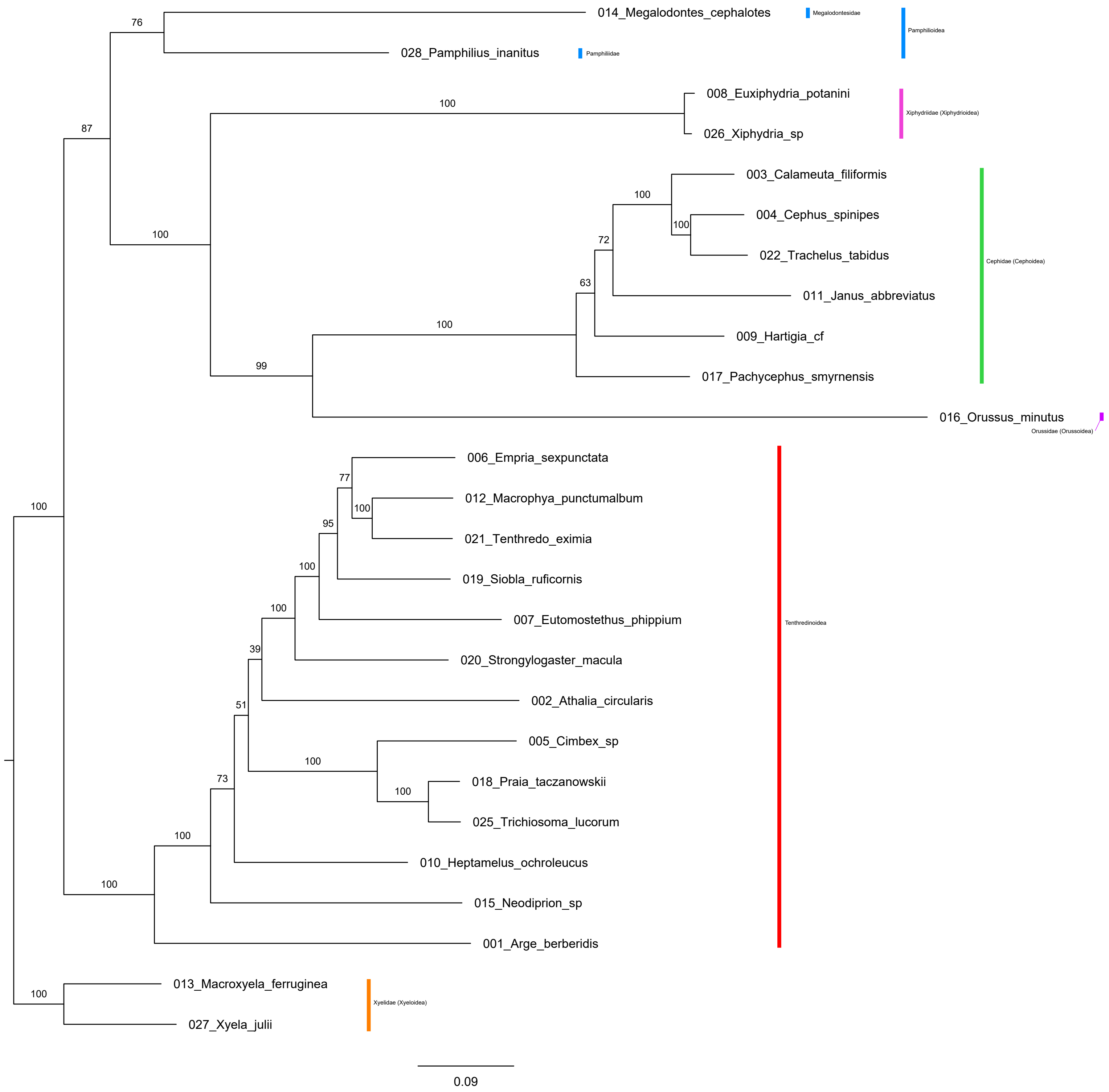

Figure S30 ML tree (RAxML) based on the amino acid sequence alignment set of 13 mtPCGs plus 8 nDNA of Matrix MNaa+O.

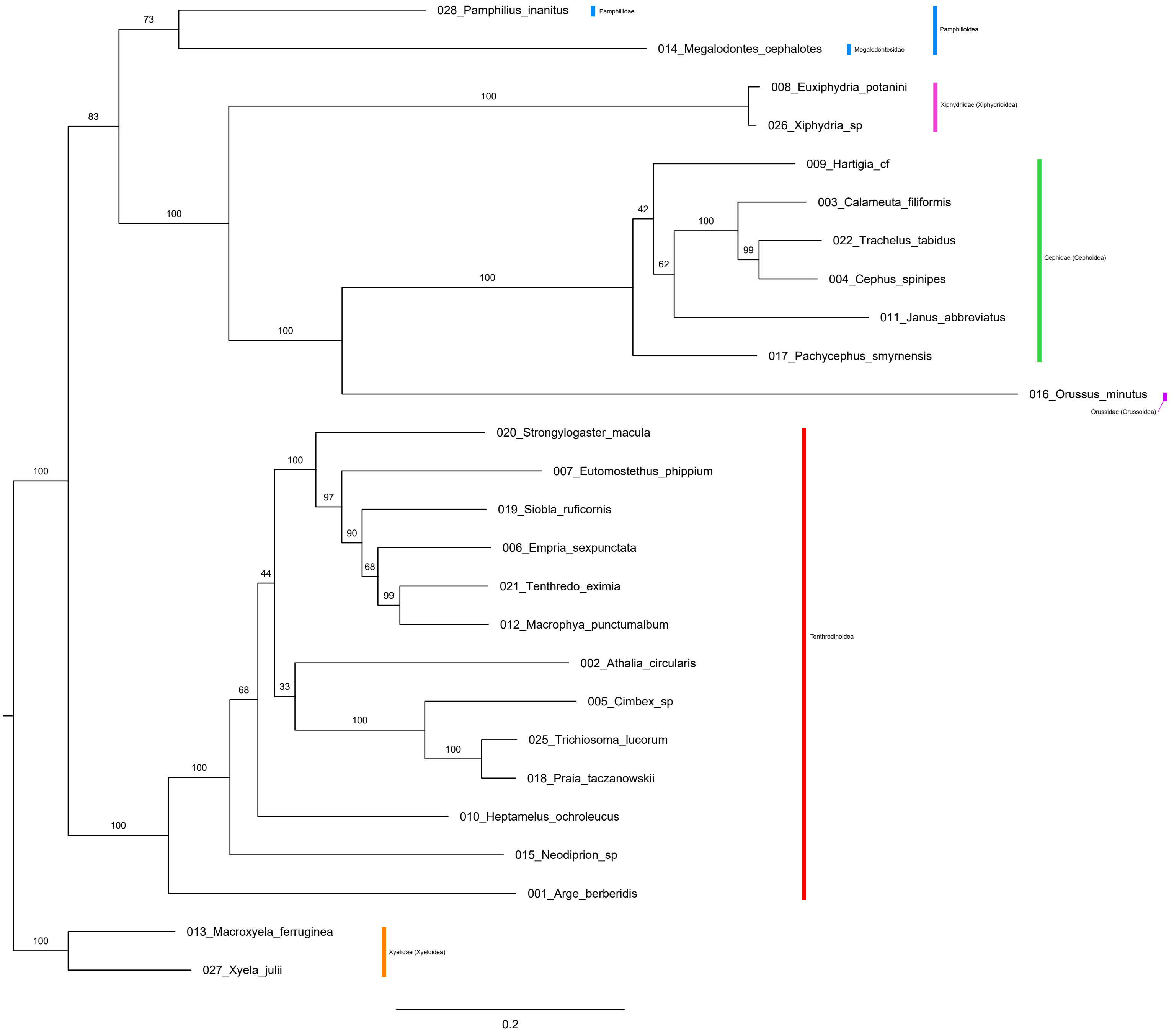

Figure S31 BI tree (MrBayes) based on the amino acid sequence alignment set of 13 mtPCGs plus 8 nDNA of Matrix MNaa+O.

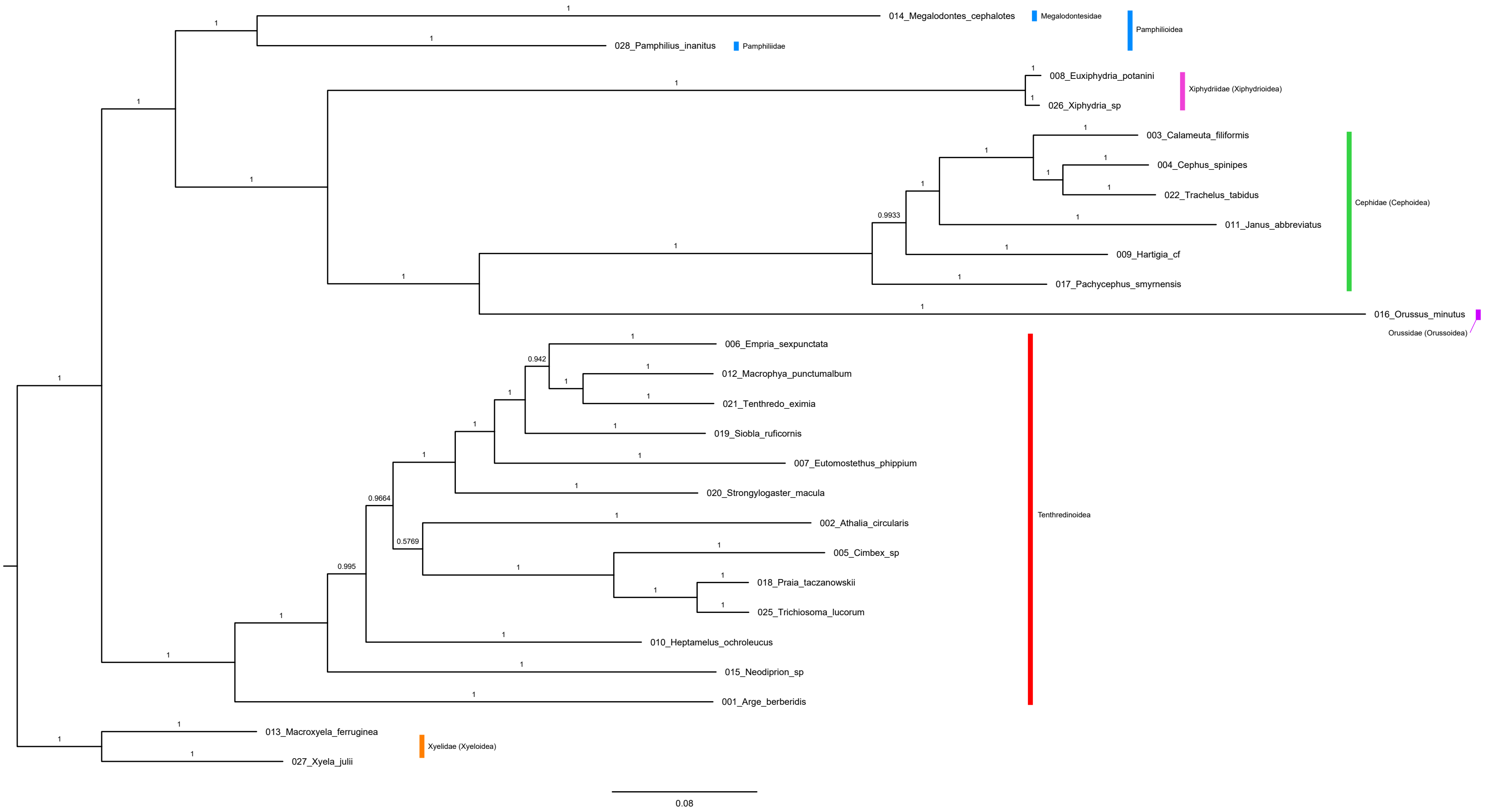

Figure S32 ML tree (IQ-TREE) based on the nucleotide sequence alignment set of 13 mtPCGs of Matrix Mnt+OS.

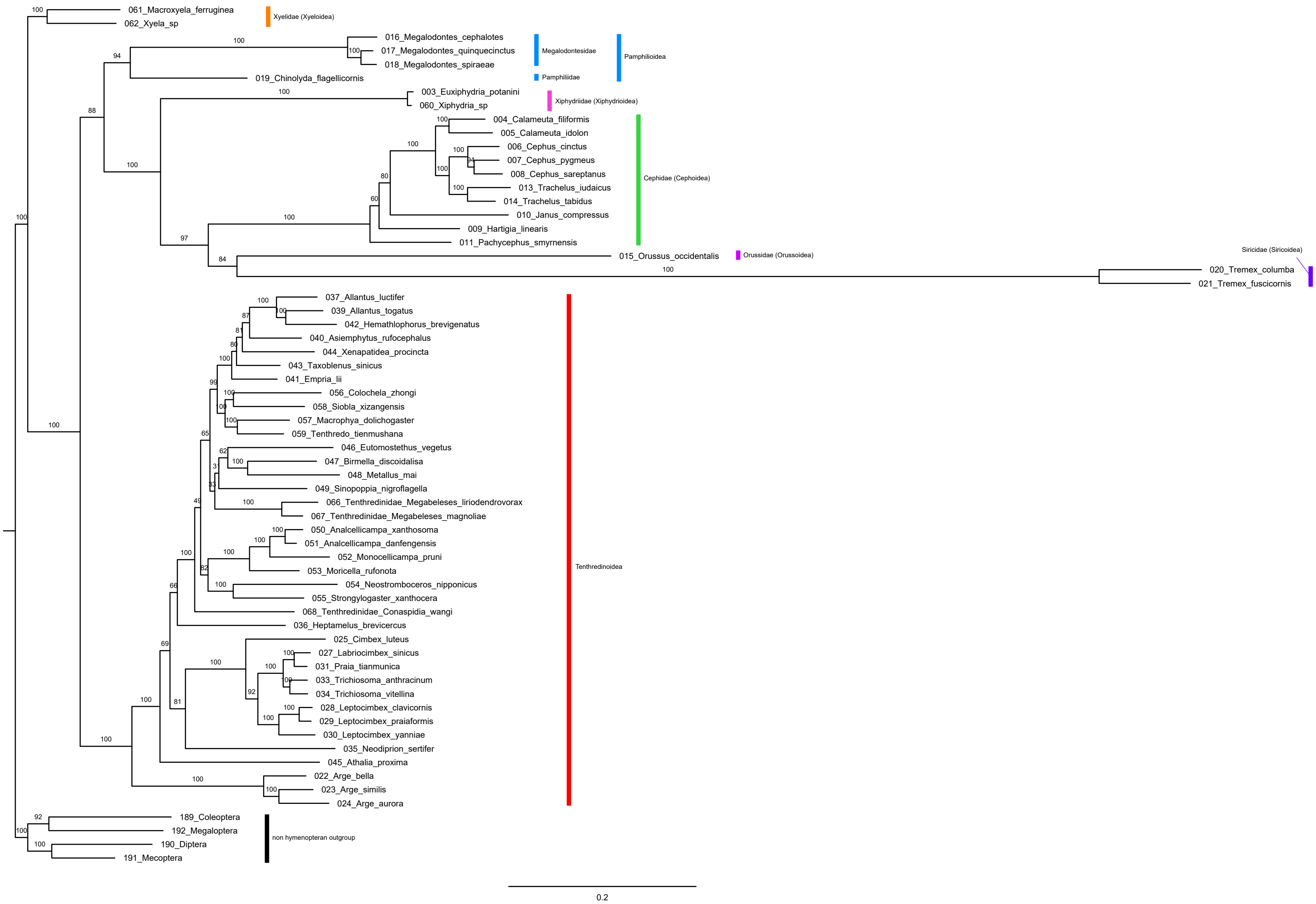

Figure S33 ML tree (RAxML) based on the nucleotide sequence alignment set of 13 mtPCGs of Matrix Mnt+OS.

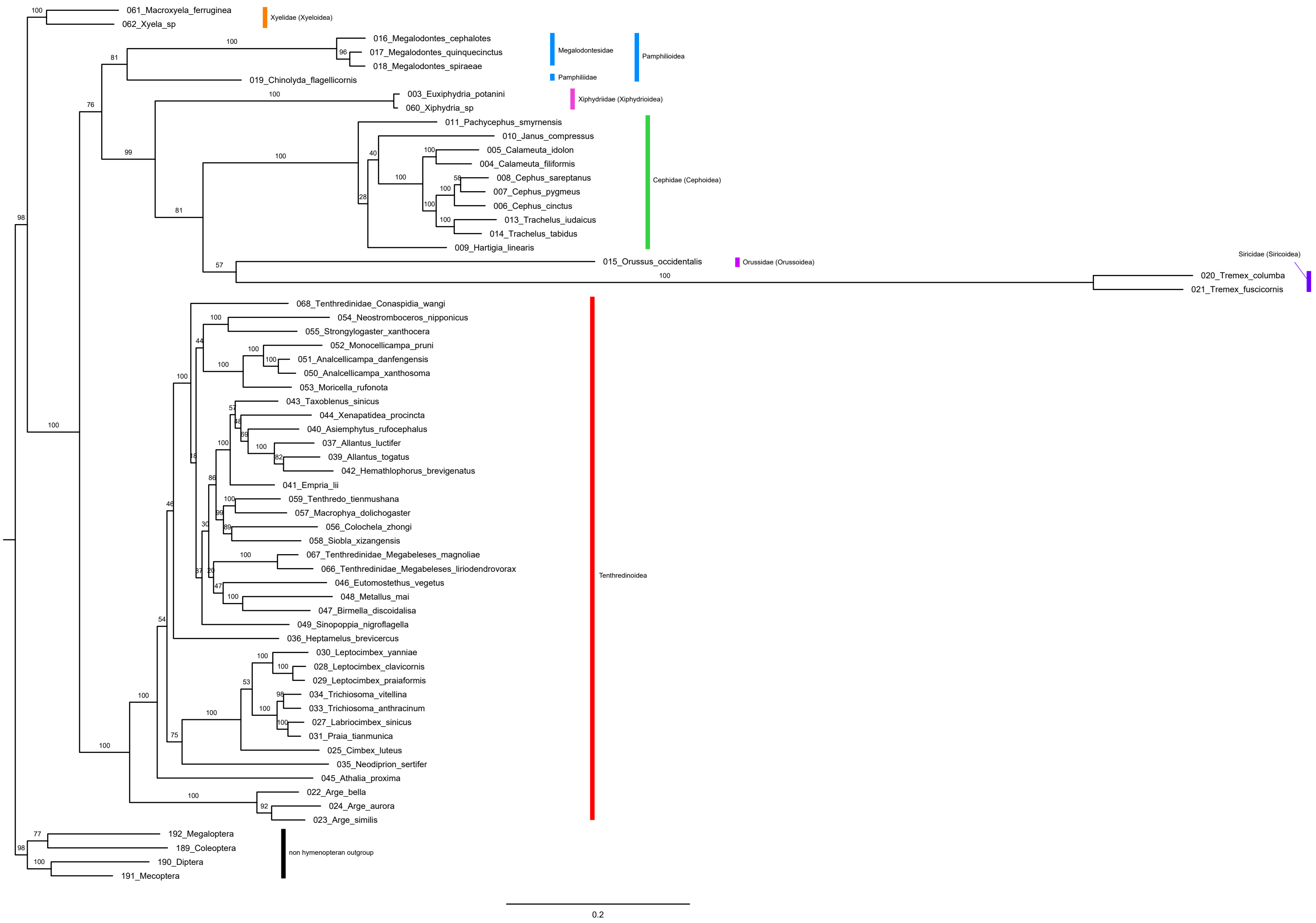

Figure S34 BI tree (MrBayes) based on the nucleotide sequence alignment set of 13 mtPCGs of Matrix Mnt+OS.

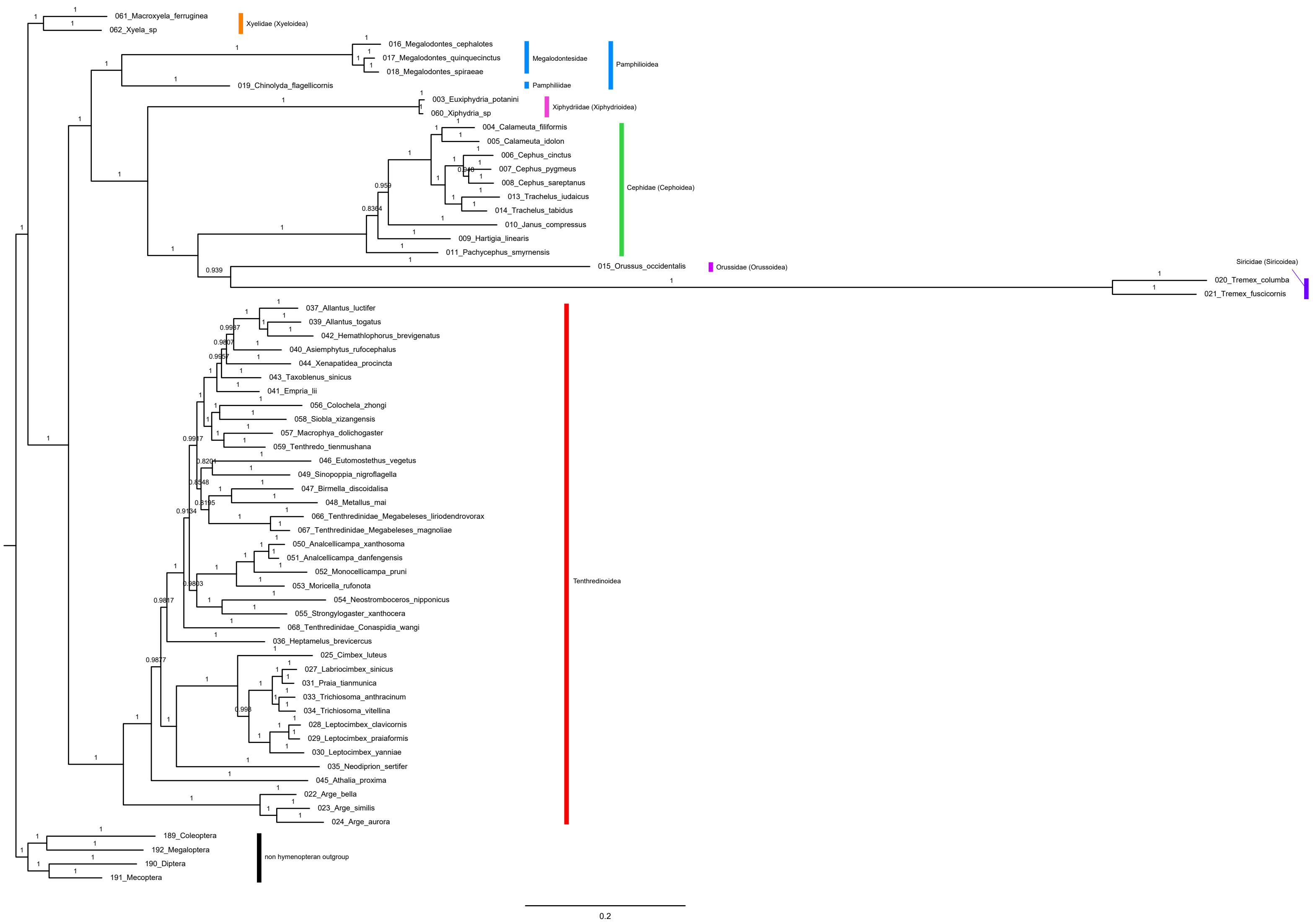

Figure S35 ML tree (IQ-TREE) based on the amino acid sequence alignment set of 13 mtPCGs of Matrix Maa+OS.

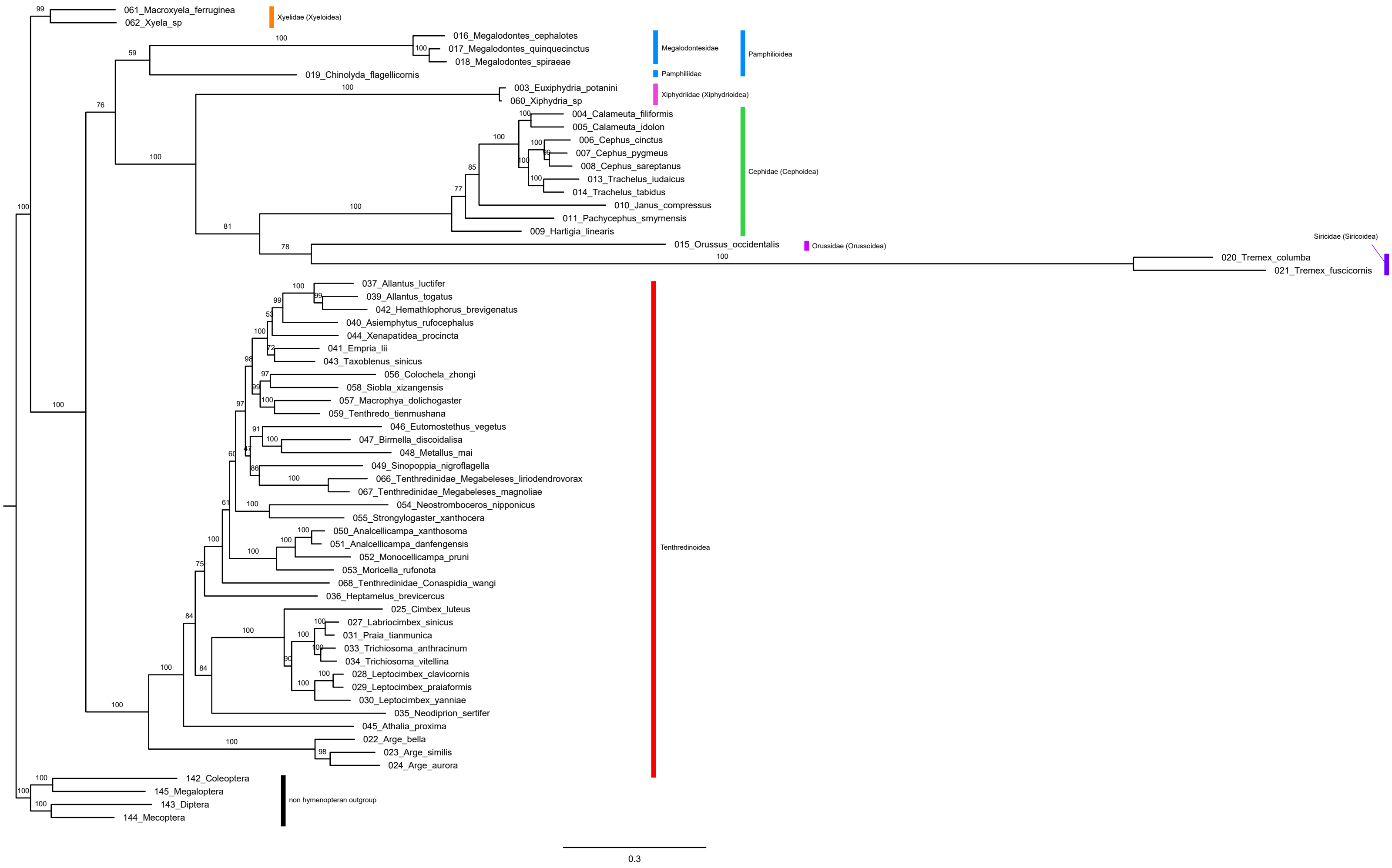

Figure S36 ML tree (RAxML) based on the amino acid sequence alignment set of 13 mtPCGs of Matrix Maa+OS.

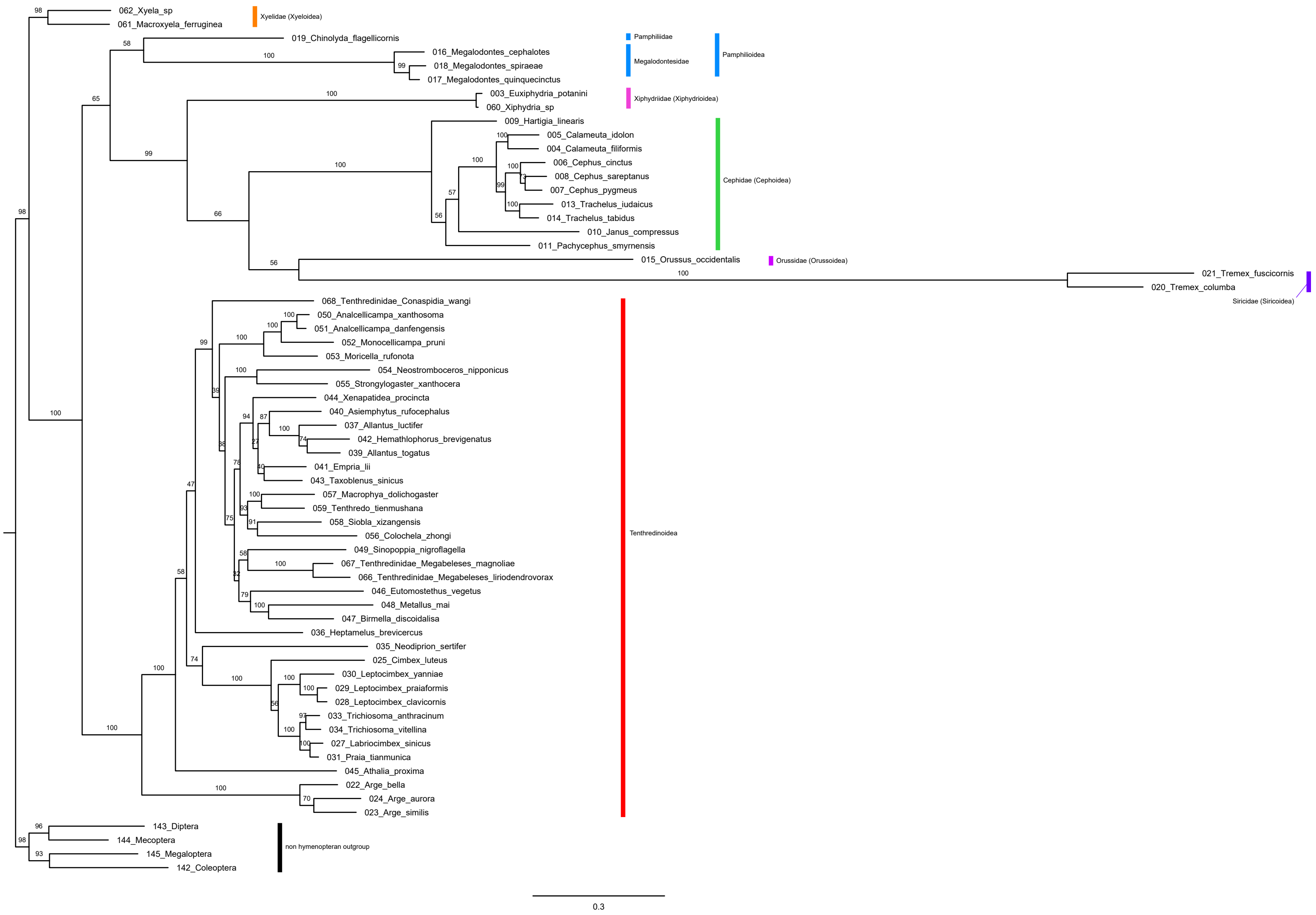

Figure S37 BI tree (MrBayes) based on the amino acid sequence alignment set of 13 mtPCGs of Matrix Maa+OS.

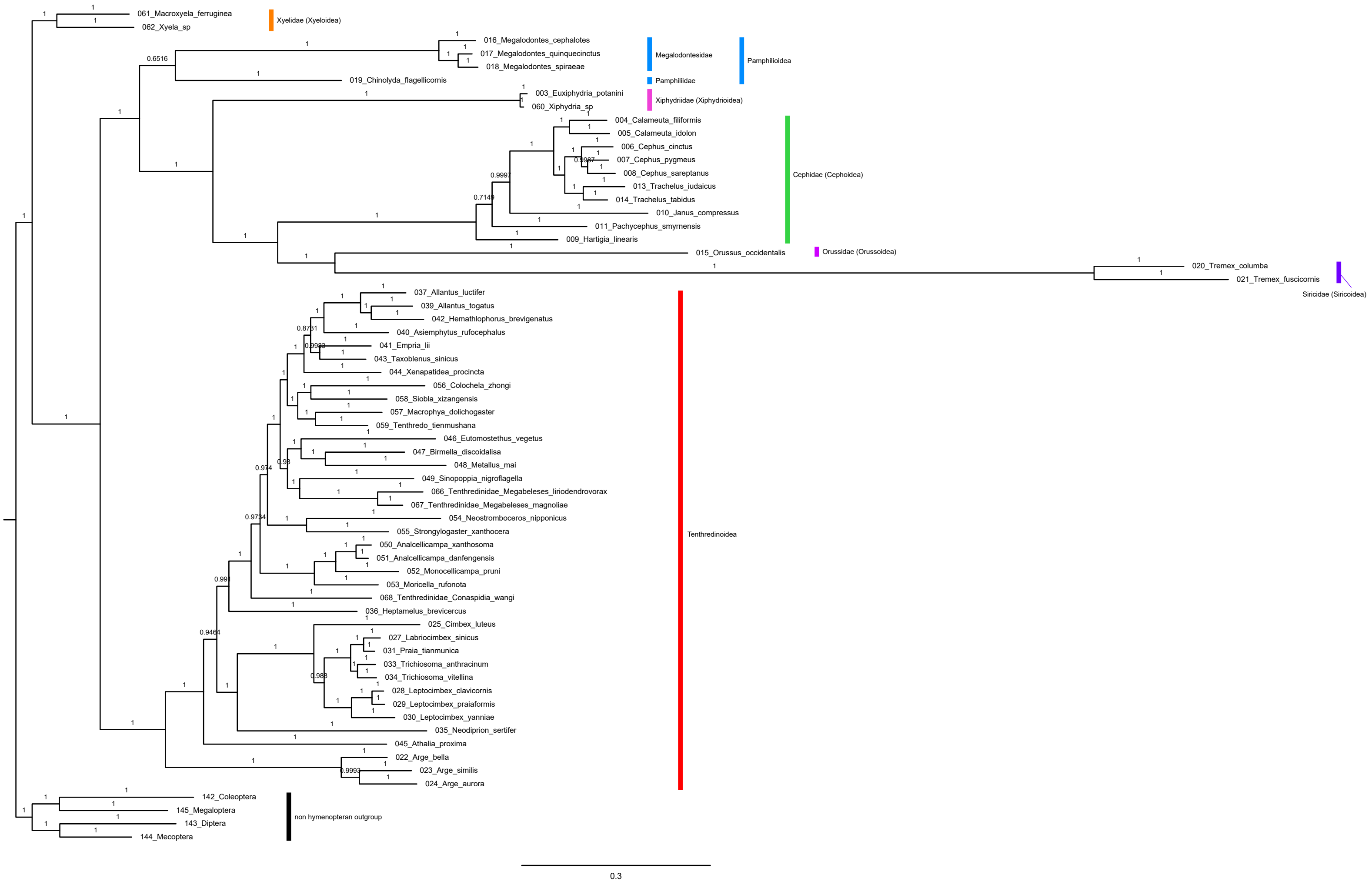

Figure S38 ML tree (IQ-TREE) based on the nucleotide sequence alignment set of 13 mtPCGs plus 8 nDNA of Matrix MNnt+OS.

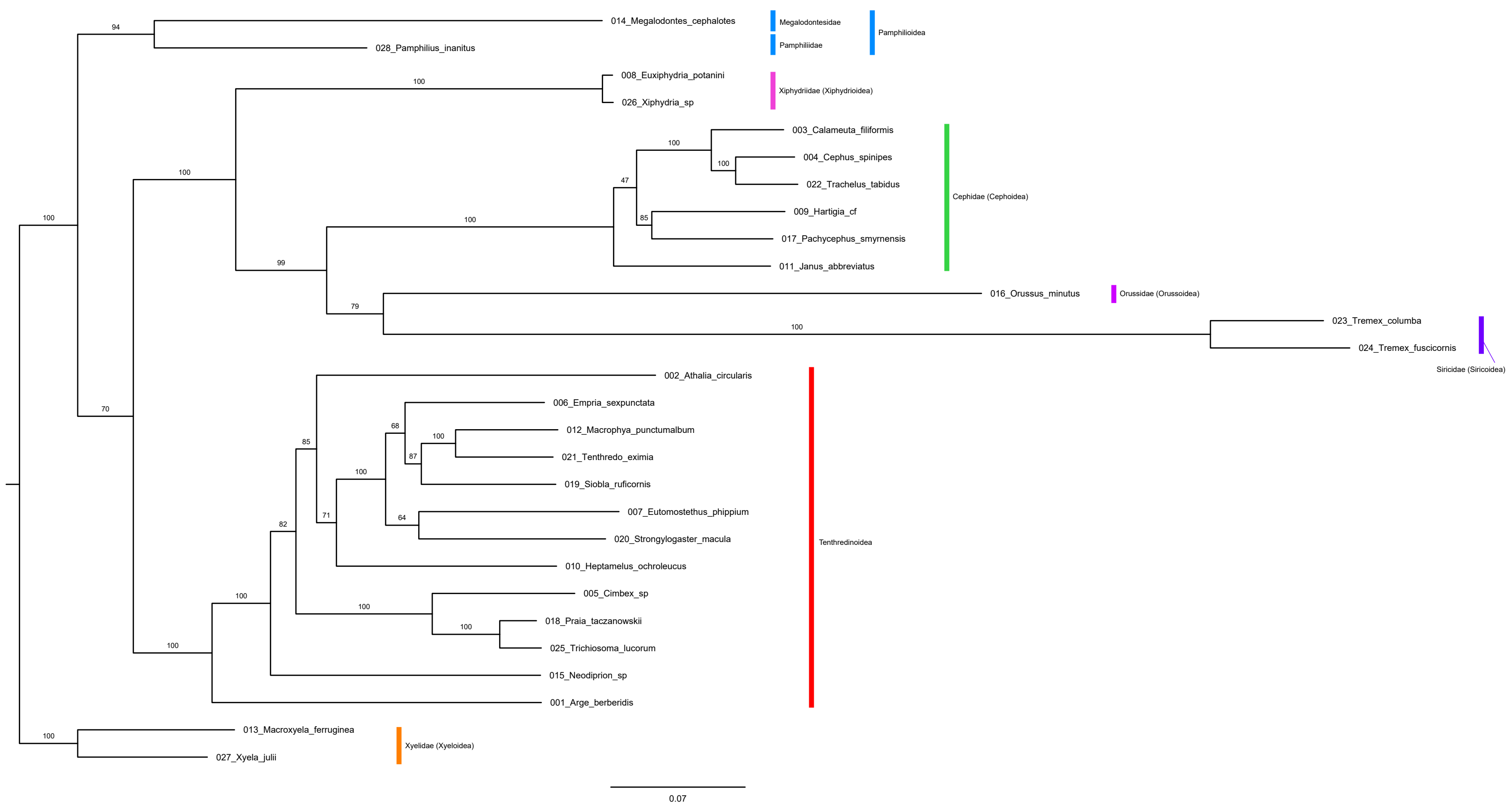

Figure S39 ML tree (RAxML) based on the nucleotide sequence alignment set of 13 mtPCGs plus 8 nDNA of Matrix MNnt+OS.

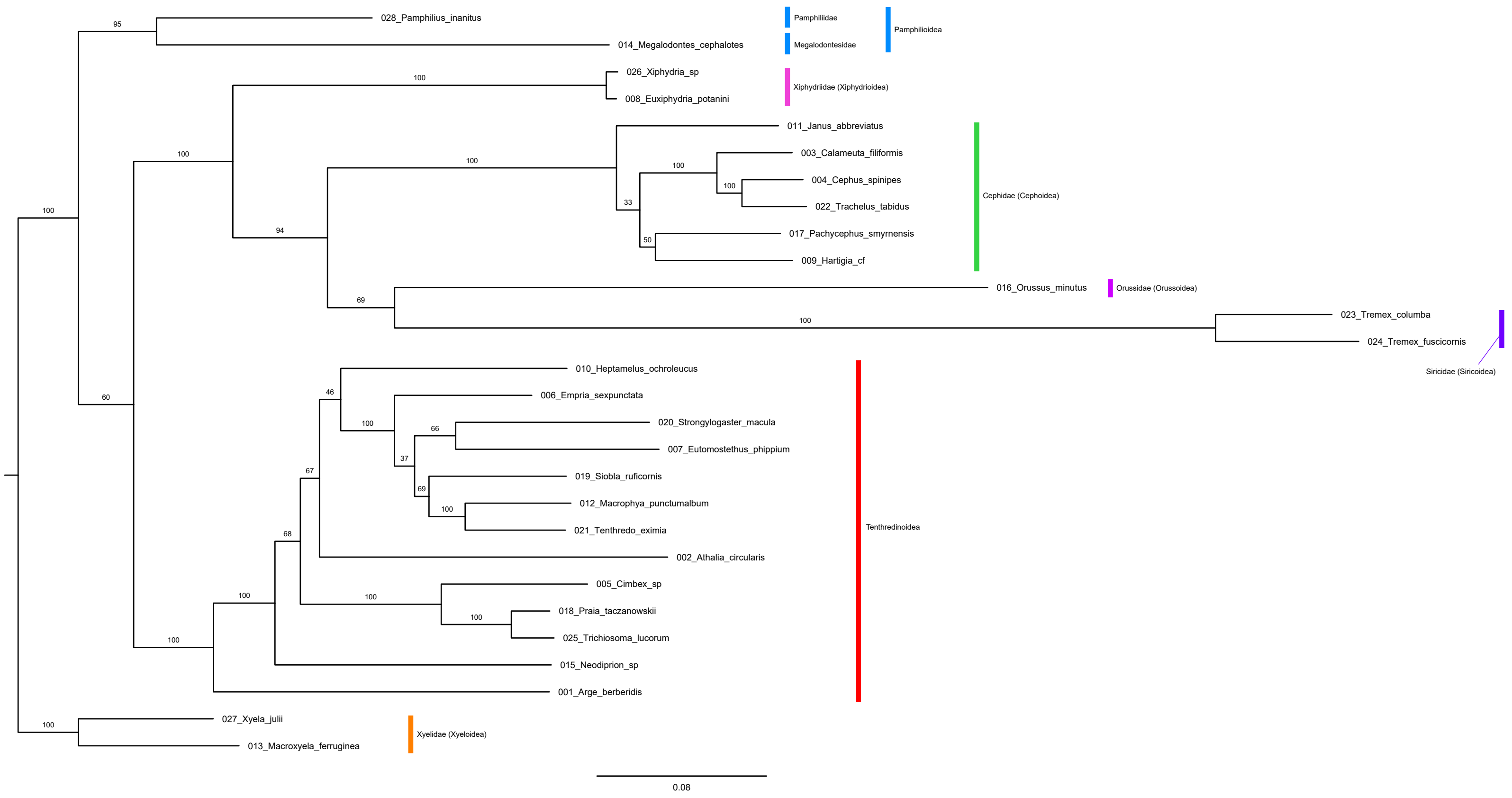

Figure S40 BI tree (MrBayes) based on the nucleotide sequence alignment set of 13 mtPCGs plus 8 nDNA of Matrix MNnt+OS.

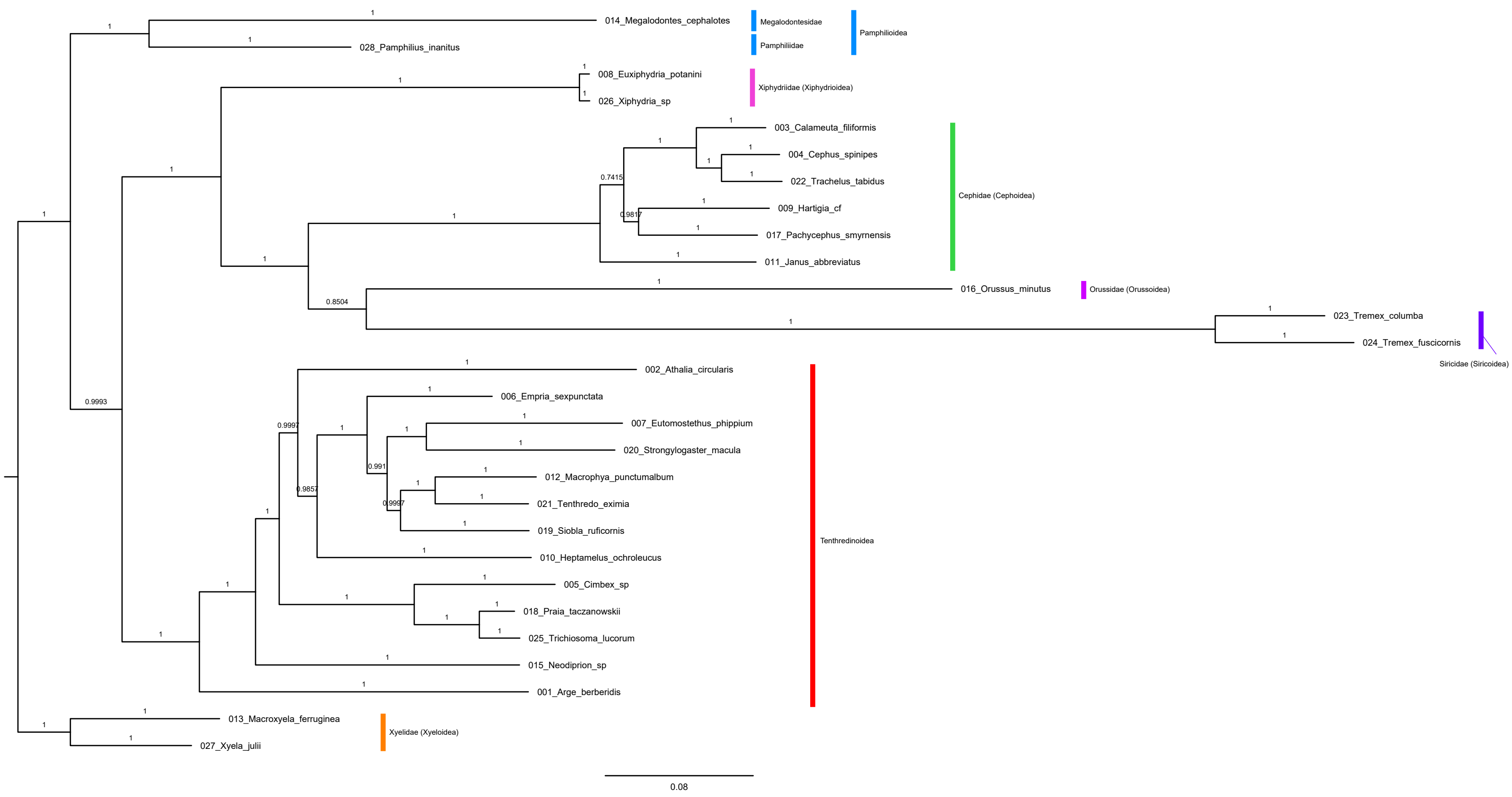

Figure S41 ML tree (IQ-TREE) based on the amino acid sequence alignment set of 13 mtPCGs plus 8 nDNA of Matrix MNaa+OS.

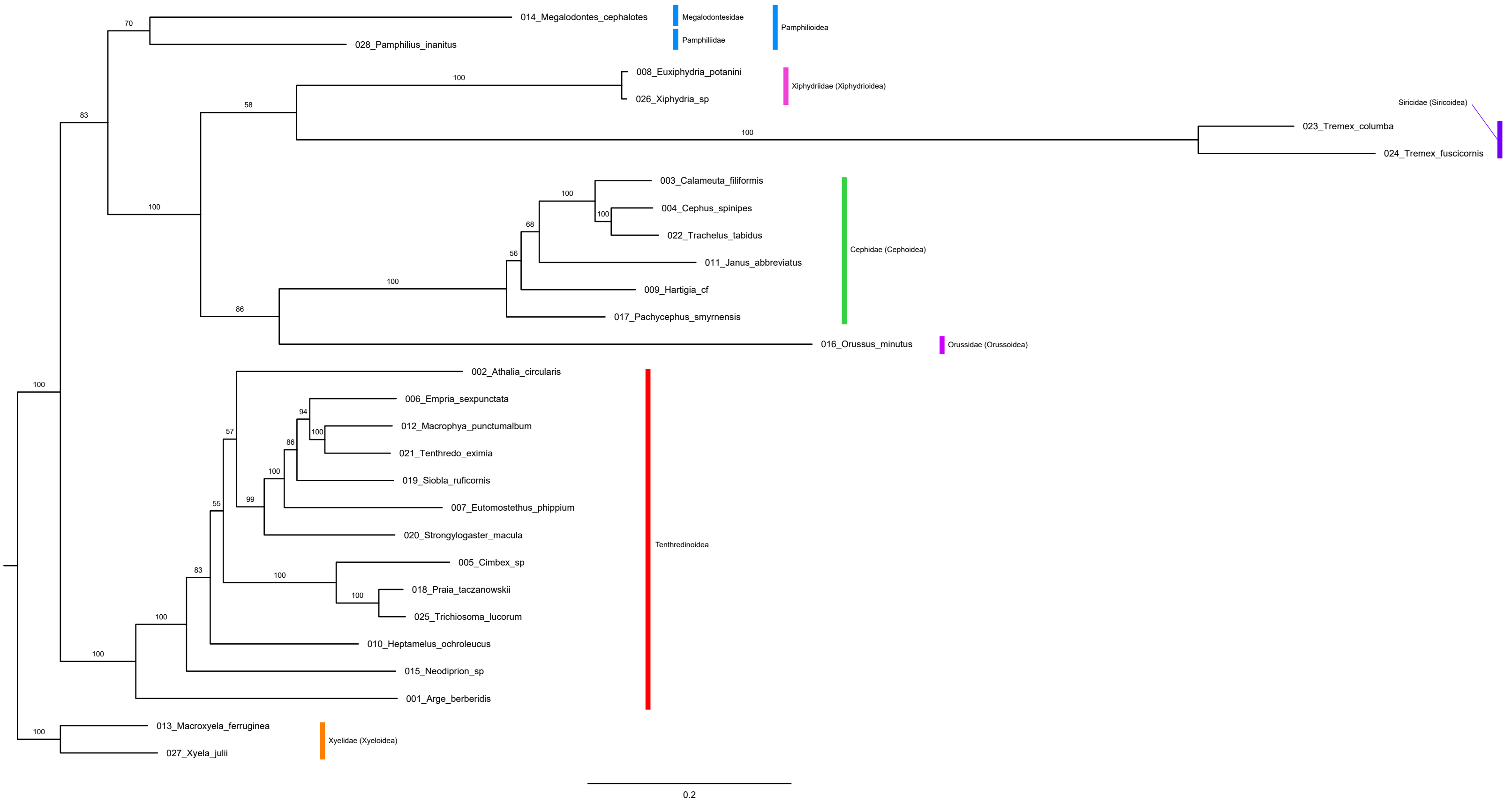

Figure S42 ML tree (RAxML) based on the amino acid sequence alignment set of 13 mtPCGs plus 8 nDNA of Matrix MNaa+OS.

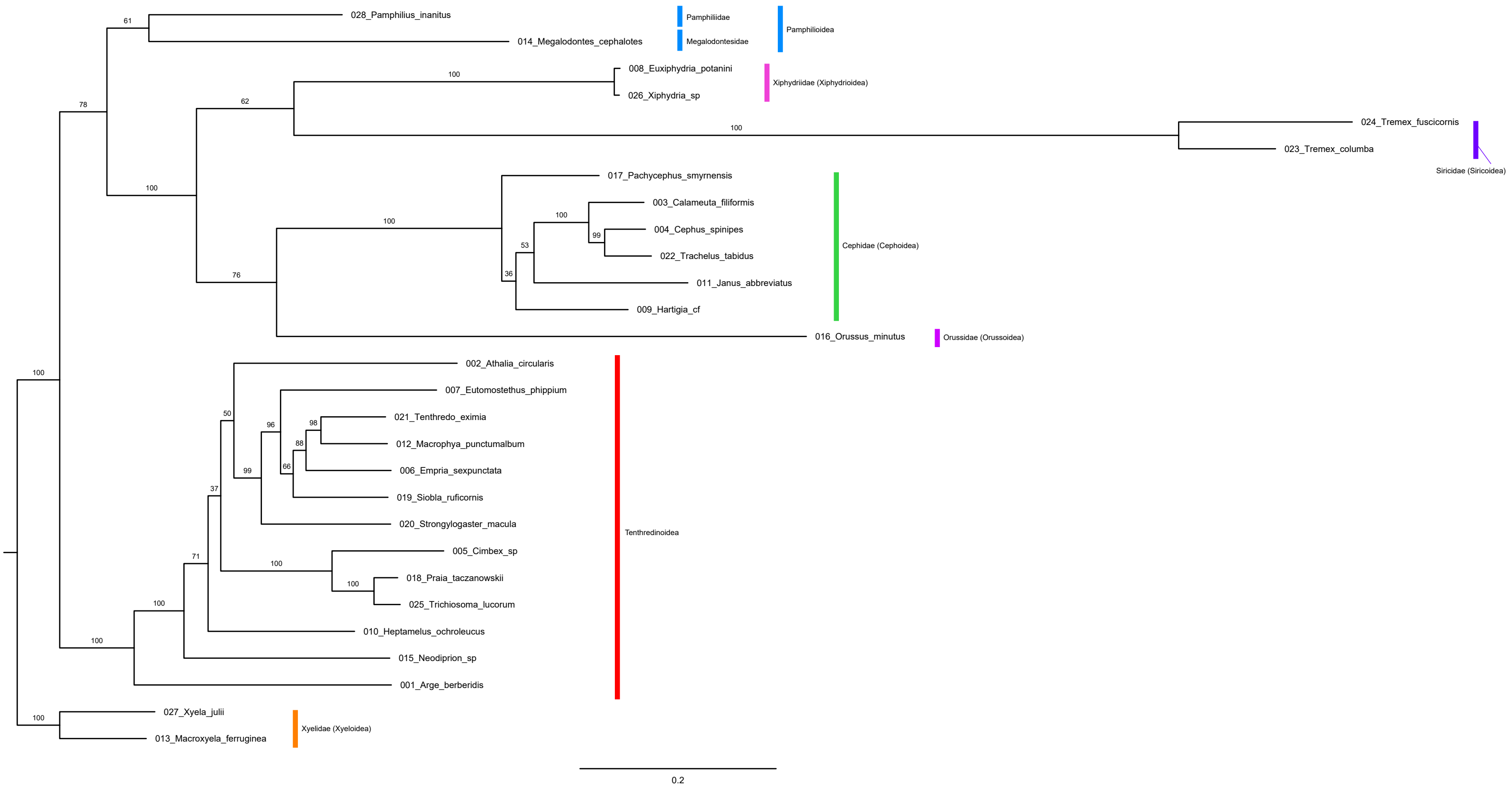

Figure S43 BI tree (MrBayes) based on the amino acid sequence alignment set of 13 mtPCGs plus 8 nDNA of Matrix MNaa+OS.

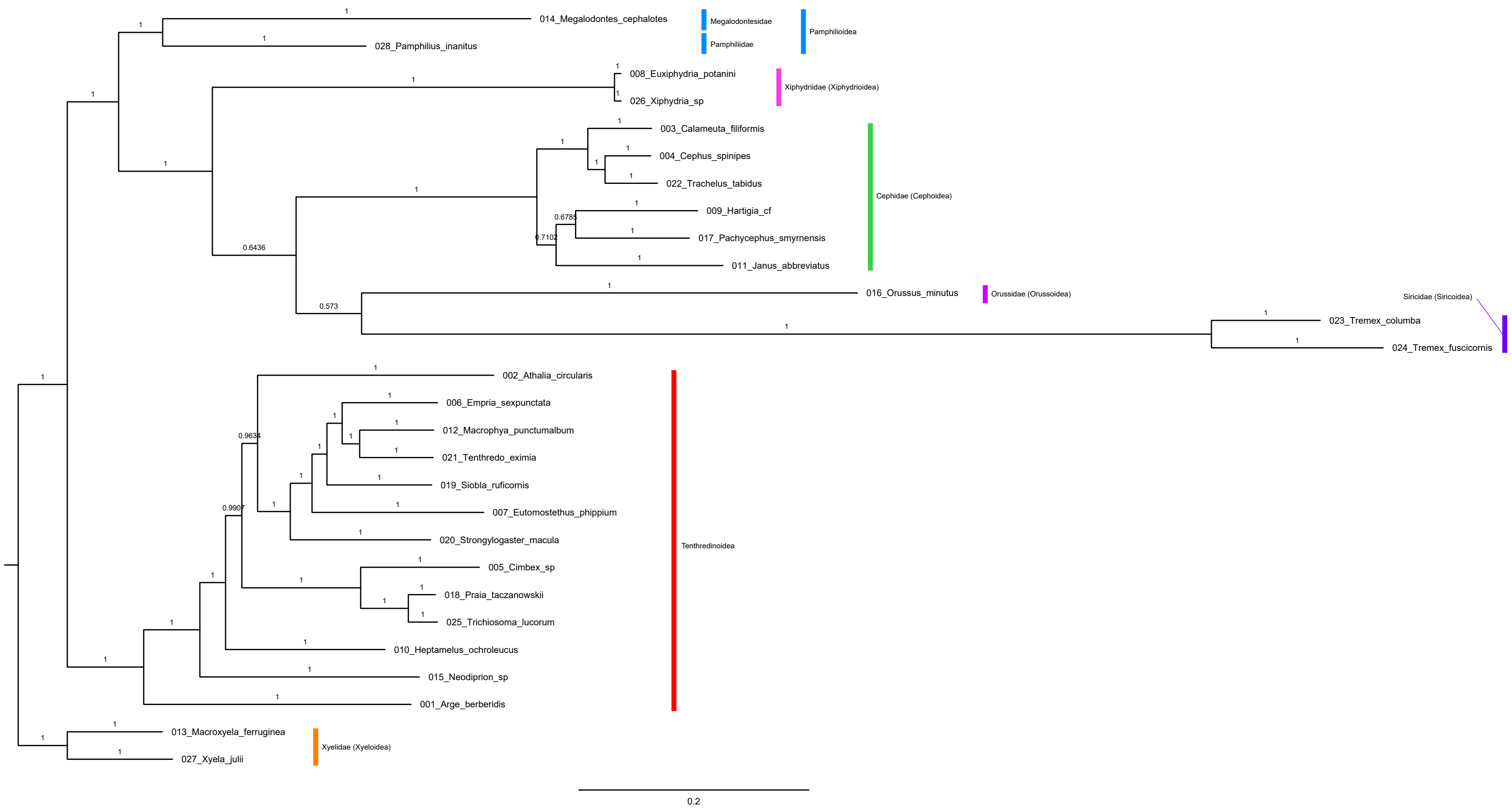

Figure S44 ML tree (IQ-TREE) based on the nucleotide sequence alignment set of 13 mtPCGs of Matrix Mnt+A.

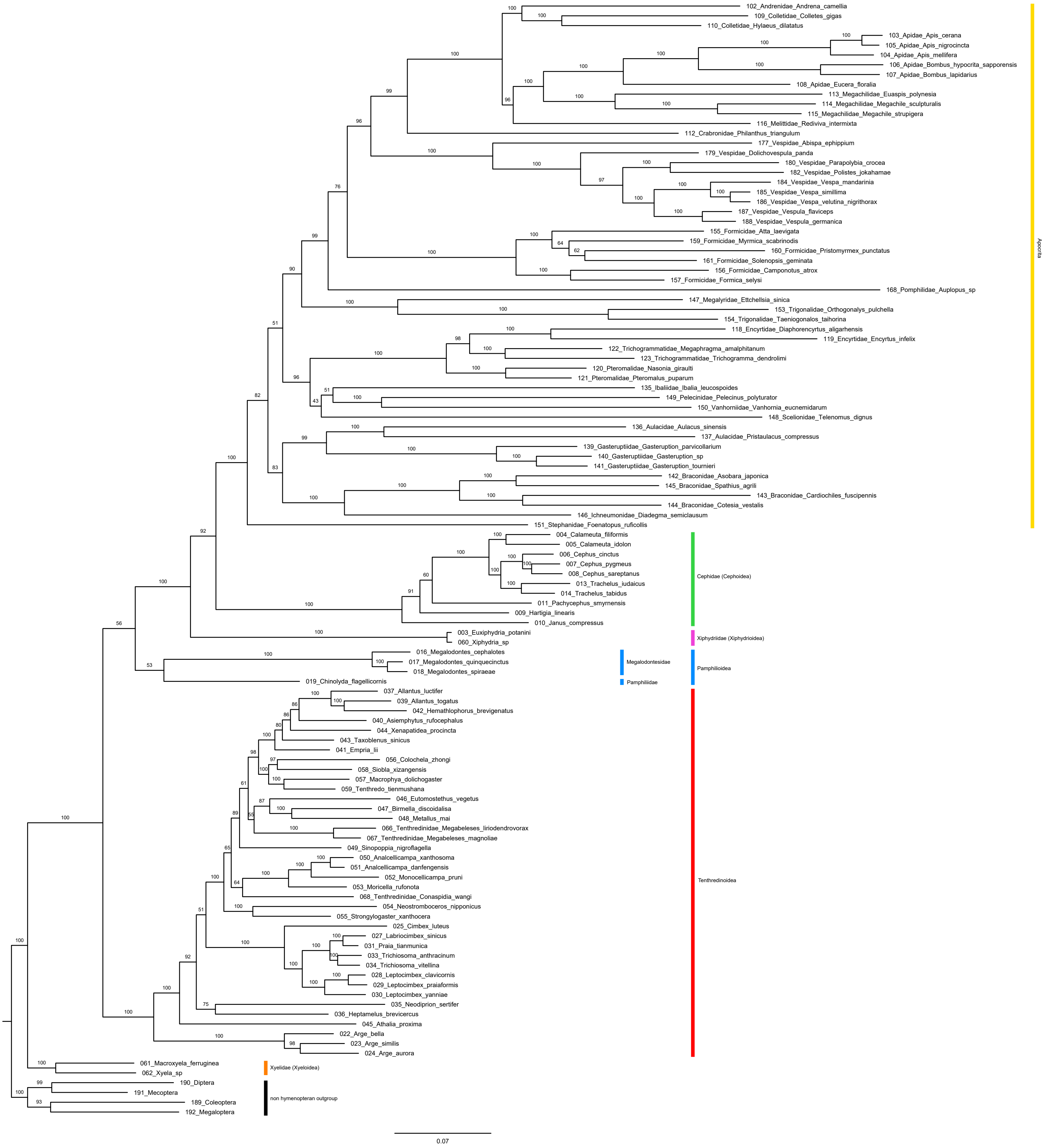

Figure S45 ML tree (RAxML) based on the nucleotide sequence alignment set of 13 mtPCGs of Matrix Mnt+A.

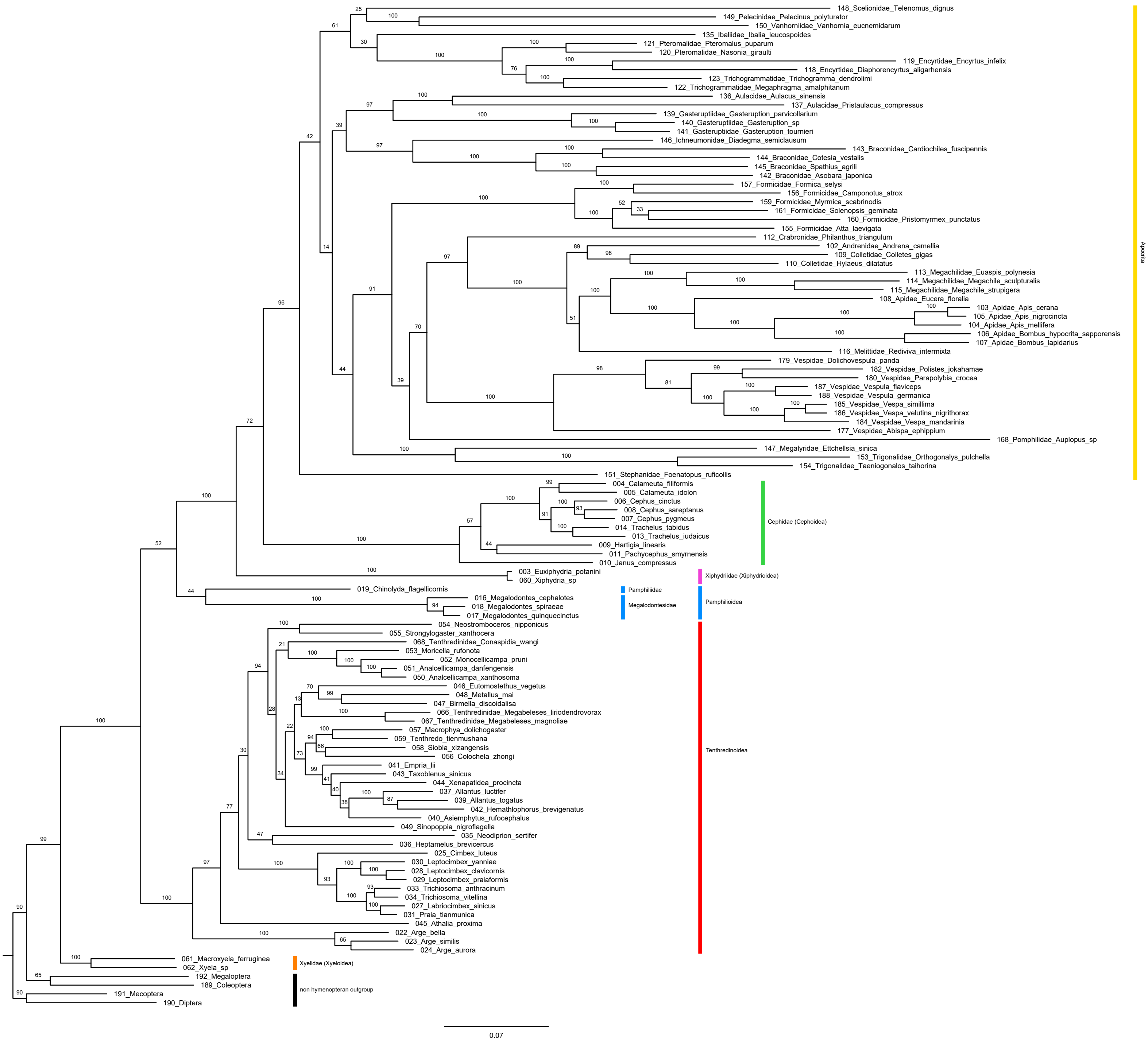

Figure S46 BI tree (MrBayes) based on the nucleotide sequence alignment set of 13 mtPCGs of Matrix Mnt+A.

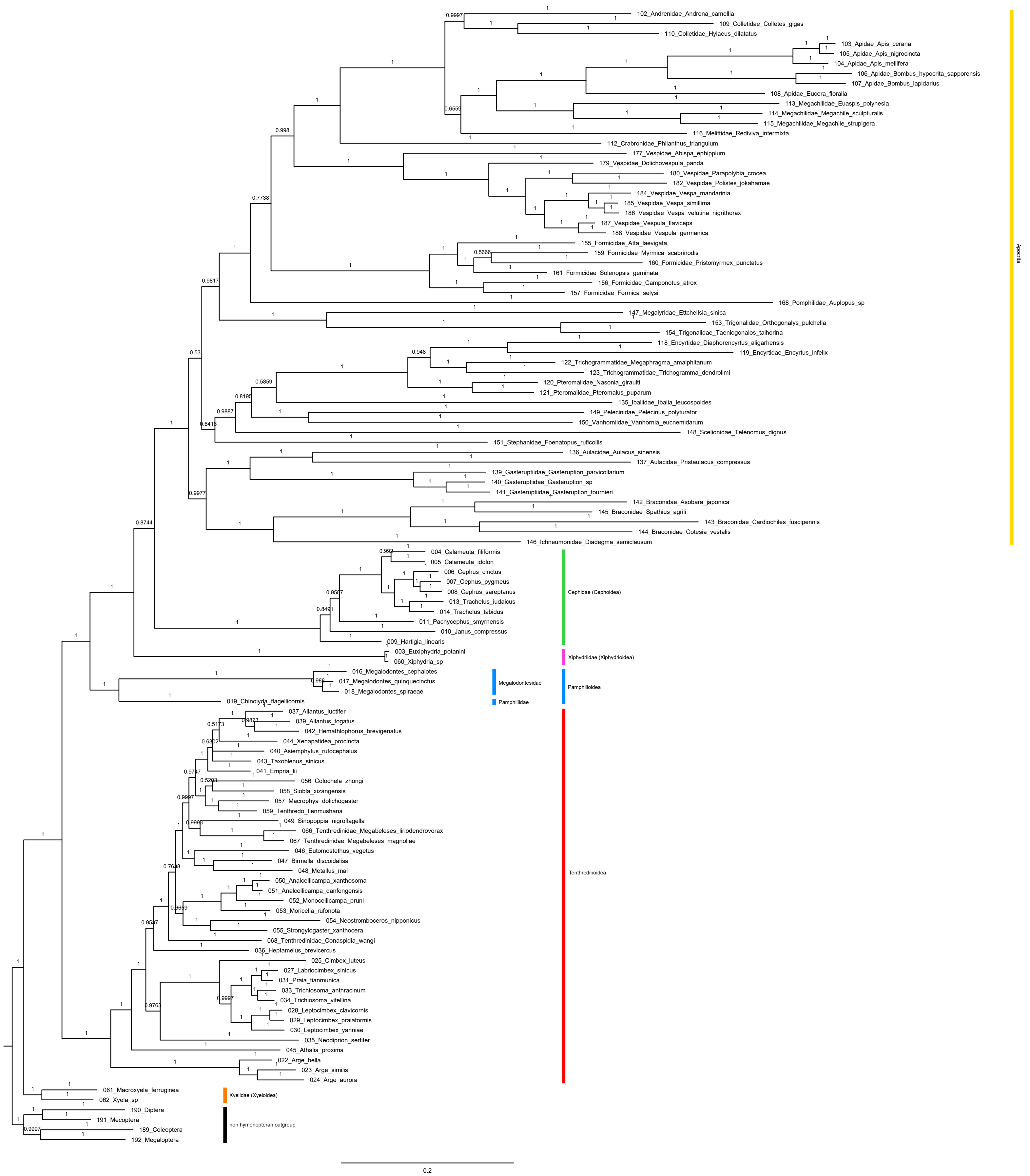

Figure S47 ML tree (IQ-TREE) based on the amino acid sequence alignment set of 13 mtPCGs of Matrix Maa+A.

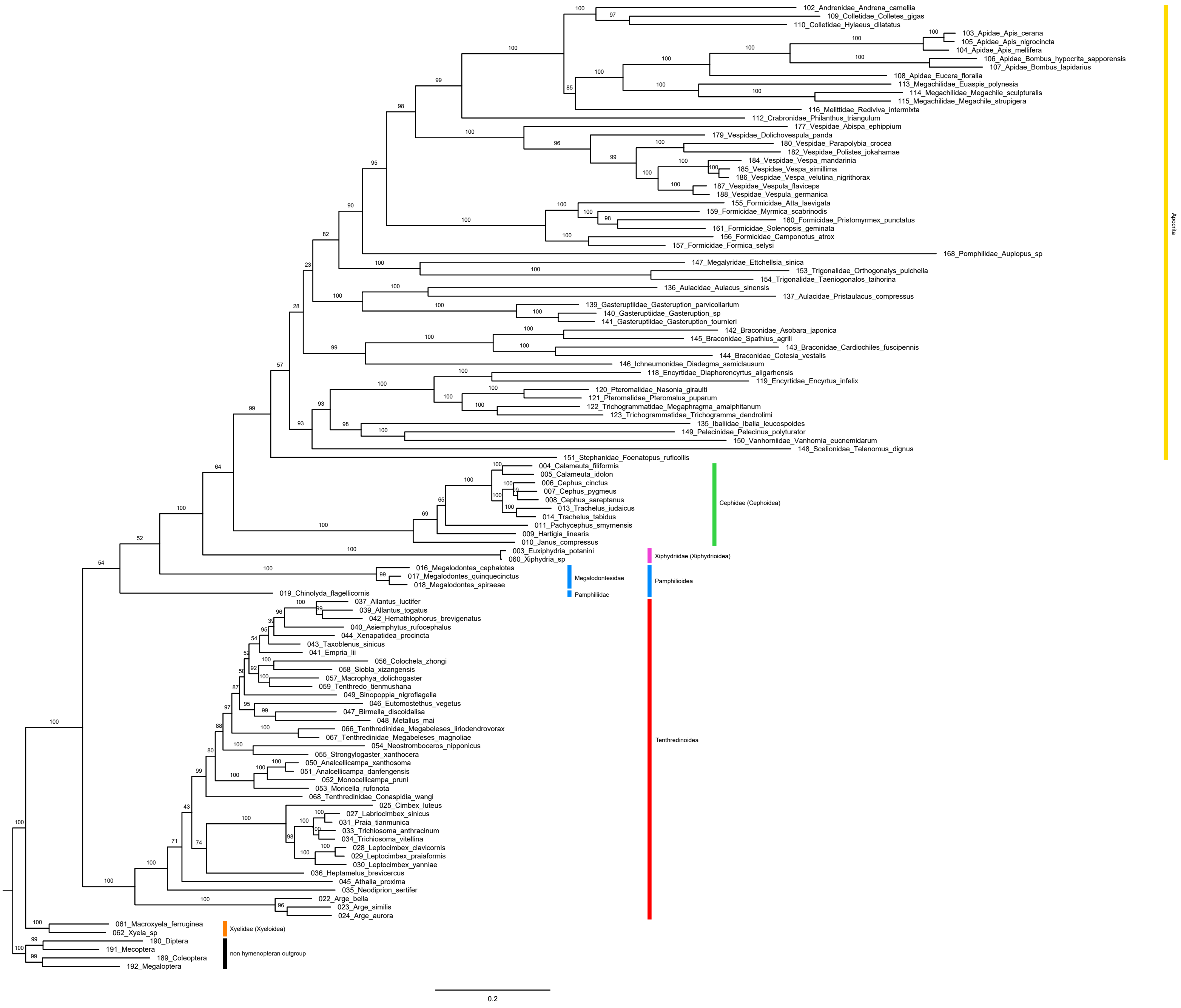

Figure S48 ML tree (RAxML) based on the amino acid sequence alignment set of 13 mtPCGs of Matrix Maa+A.

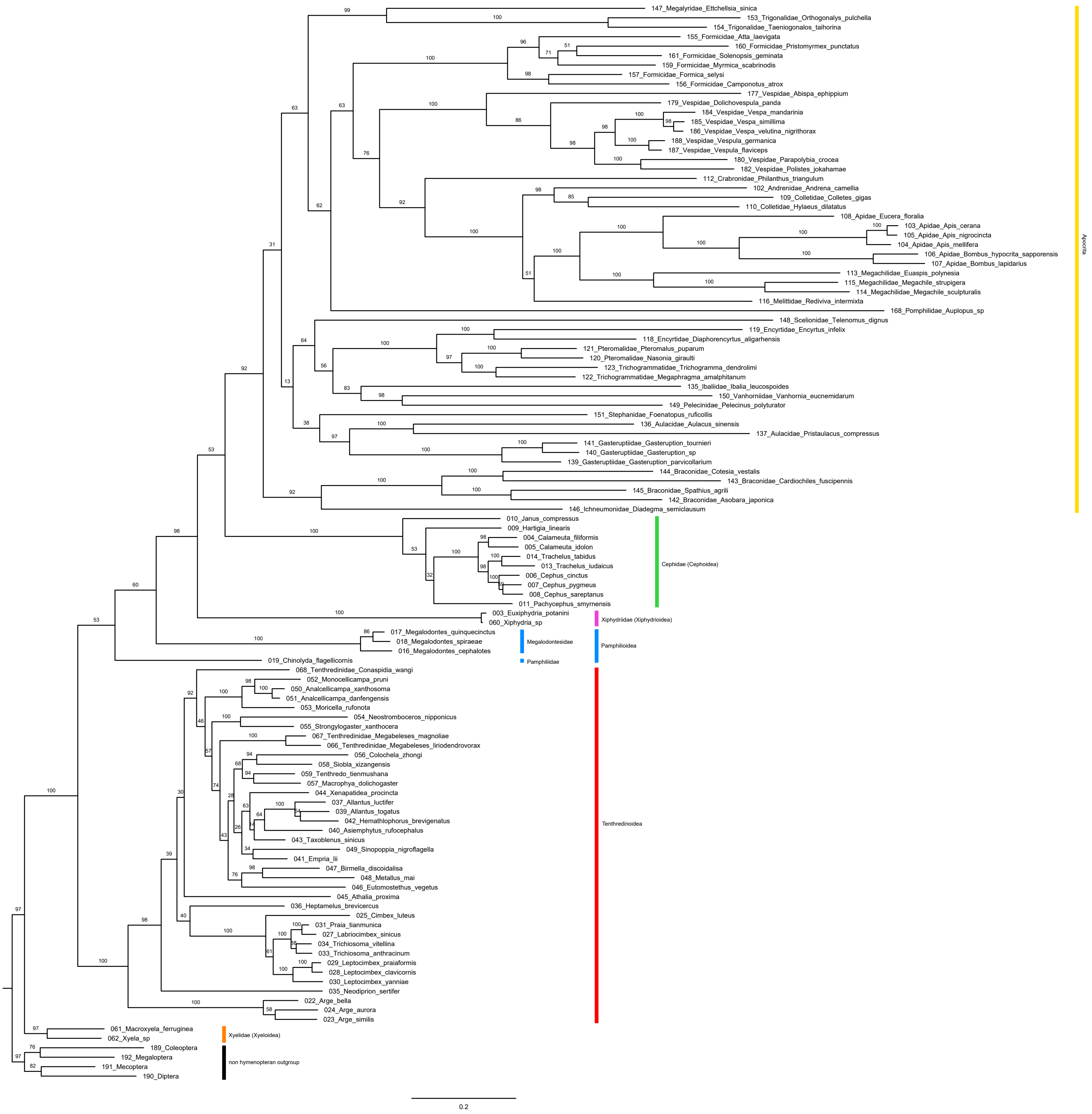

Figure S49 BI tree (MrBayes) based on the amino acid sequence alignment set of 13 mtPCGs of Matrix Maa+A.

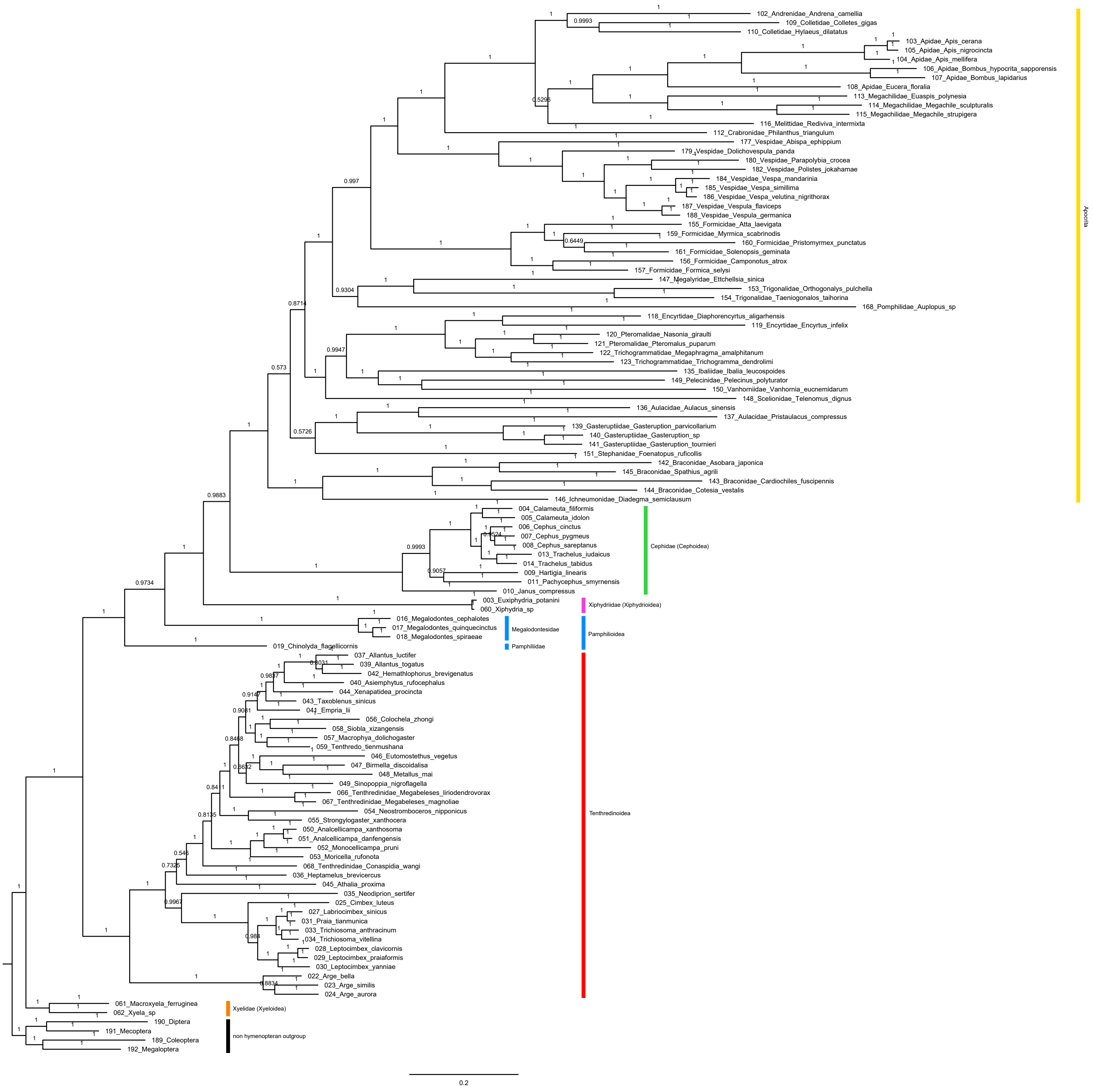

Table S1. Taxa examined in this study, lengths of mitochondrial genomes, accession numbers, and the included taxa for the initial dataset, Matrices M and NM. The initial dataset is included the members of the superfamilies Orussoidea and Siricoidea, whereas Matrices M and MN datasets are excluded the members of two symphytan superfamilies. The gray color is excluded species from Matrix M+A. Asterisks indicate partial or nearly complete genomes.

**Order**	**Suborder**	**Superfamily**	**Family**	**Species**	**Length (bp)**	**NCBI no.**	**Initial dataset (123 taxa)**	**Matrix M (60 taxa)**	**Matrix MN (25 taxa)**
Hymenoptera	Symphyta	Cephoidea	Cephidae	*Calameuta filiformis*	20,055	KT260167	√	√	√
Hymenoptera	Symphyta	Cephoidea	Cephidae	*Calameuta idolon*	19,746	KT260168	√	√	
Hymenoptera	Symphyta	Cephoidea	Cephidae	*Cephus cinctus*	19,339	FJ478173	√	√	√
Hymenoptera	Symphyta	Cephoidea	Cephidae	*Cephus pygmeus**	16,145	KM377623	√	√	
Hymenoptera	Symphyta	Cephoidea	Cephidae	*Cephus sareptanus**	15,212	KM377624	√	√	
Hymenoptera	Symphyta	Cephoidea	Cephidae	*Hartigia linearis**	20,116	KX907843	√	√	√
Hymenoptera	Symphyta	Cephoidea	Cephidae	*Janus compressus**	16,700	KX907844	√	√	√
Hymenoptera	Symphyta	Cephoidea	Cephidae	*Pachycephus smyrnensis**	15,203	KX907846	√	√	√
Hymenoptera	Symphyta	Cephoidea	Cephidae	*Trachelus iudaicus*	20,370	KX257357	√	√	
Hymenoptera	Symphyta	Cephoidea	Cephidae	*Trachelus tabidus*	18,539	KX257358	√	√	√
Hymenoptera	Symphyta	Orussoidea	Orussidae	*Orussus occidentalis*	15,947	FJ478174	√		
Hymenoptera	Symphyta	Pamphilioidea	Megalodontesidae	*Megalodontes cephalotes**	16,890	MH577058	√	√	√
Hymenoptera	Symphyta	Pamphilioidea	Megalodontesidae	*Megalodontes quinquecinctus**	17,033	MG923502	√	√	
Hymenoptera	Symphyta	Pamphilioidea	Megalodontesidae	*Megalodontes spiraeae**	15,980	MH577059	√	√	
Hymenoptera	Symphyta	Pamphilioidea	Pamphiliidae	*Chinolyda flagellicornis**	16,523	MH577057	√	√	√
Hymenoptera	Symphyta	Siricoidea	Siricidae	*Tremex columba*	16,397	MH422968	√		
Hymenoptera	Symphyta	Siricoidea	Siricidae	*Tremex fuscicornis*	16,396	MT425058	√		
Hymenoptera	Symphyta	Tenthredinoidea	Argidae	*Arge bella*	15,576	MF287761	√	√	
Hymenoptera	Symphyta	Tenthredinoidea	Argidae	*Arge similis**	15,707	MG923484	√	√	√
Hymenoptera	Symphyta	Tenthredinoidea	Argidae	*Arge* sp./*A. aurora*	15,675	MN913350	√	√	
Hymenoptera	Symphyta	Tenthredinoidea	Cimbicidae	*Cimbex luteus**	15,193	MW136447	√	√	√
Hymenoptera	Symphyta	Tenthredinoidea	Cimbicidae	*Labriocimbex sinicus*	15,405	MH136623	√	√	
Hymenoptera	Symphyta	Tenthredinoidea	Cimbicidae	*Leptocimbex clavicornis**	15,253	MT478109	√	√	
Hymenoptera	Symphyta	Tenthredinoidea	Cimbicidae	*Leptocimbex praiaformis**	15,056	MT478110	√	√	
Hymenoptera	Symphyta	Tenthredinoidea	Cimbicidae	*Leptocimbex yanniae**	15,259	MT478111	√	√	
Hymenoptera	Symphyta	Tenthredinoidea	Cimbicidae	*Praia tianmunica*	15,556	MT665975	√	√	√
Hymenoptera	Symphyta	Tenthredinoidea	Cimbicidae	*Trichiosoma anthracinum*	15,392	KT921411	√	√	√
Hymenoptera	Symphyta	Tenthredinoidea	Cimbicidae	*Trichiosoma vitellina**	15,245	MN853777	√	√	
Hymenoptera	Symphyta	Tenthredinoidea	Diprionidae	*Neodiprion sertifer*	16,461	MK994526	√	√	√
Hymenoptera	Symphyta	Tenthredinoidea	Heptamelidae	*Heptamelus* sp./*H. brevicercus*	15,255	MW632128	√	√	√

Table S1. (Continued)

Order	Suborder	Superfamily	Family	Species	Length (bp)	NCBI no.	Initial dataset (123 taxa)	Matrix M (60 taxa)	Matrix MN (25 taxa)
Hymenoptera	Symphyta	Tenthredinoidea	Tenthredinidae	*Allantus luctifer*	15,418	NC_024664	√	√	
Hymenoptera	Symphyta	Tenthredinoidea	Tenthredinidae	*Allantus togatus*	15,480	MW464859	√	√	
Hymenoptera	Symphyta	Tenthredinoidea	Tenthredinidae	*Analcellicampa danfengensis*	15,968	MN163004	√	√	
Hymenoptera	Symphyta	Tenthredinoidea	Tenthredinidae	*Analcellicampa xanthosoma**	15,512	MH992752	√	√	
Hymenoptera	Symphyta	Tenthredinoidea	Tenthredinidae	*Asiemphytus rufocephalus**	14,864	KR703582	√	√	
Hymenoptera	Symphyta	Tenthredinoidea	Tenthredinidae	*Athalia proxima*	15,852	NC_045360	√	√	√
Hymenoptera	Symphyta	Tenthredinoidea	Tenthredinidae	*Birmella discoidalisa**	15,113	MF197548	√	√	
Hymenoptera	Symphyta	Tenthredinoidea	Tenthredinidae	*Colochela zhongi**	15,095	MT702984	√	√	
Hymenoptera	Symphyta	Tenthredinoidea	Tenthredinidae	*Conaspidia wangi*	15,924	MW415019	√	√	
Hymenoptera	Symphyta	Tenthredinoidea	Tenthredinidae	*Empria* sp./*E. lii*	15,116	MW632124	√	√	√
Hymenoptera	Symphyta	Tenthredinoidea	Tenthredinidae	*Eutomostethus vegetus**	16,345	MT663219	√	√	√
Hymenoptera	Symphyta	Tenthredinoidea	Tenthredinidae	*Hemathlophorus* sp./*H. brevigenatus*	15,452	MW632125	√	√	
Hymenoptera	Symphyta	Tenthredinoidea	Tenthredinidae	*Macrophya dolichogaster**	15,399	MW544890	√	√	√
Hymenoptera	Symphyta	Tenthredinoidea	Tenthredinidae	*Megabeleses liriodendrovorax*	16,019	MW255939	√	√	
Hymenoptera	Symphyta	Tenthredinoidea	Tenthredinidae	*Megabeleses magnoliae*	15,918	MW255940	√	√	
Hymenoptera	Symphyta	Tenthredinoidea	Tenthredinidae	*Metallus mai**	15,463	MW255941	√	√	
Hymenoptera	Symphyta	Tenthredinoidea	Tenthredinidae	*Monocellicampa pruni**	15,169	JX566509	√	√	
Hymenoptera	Symphyta	Tenthredinoidea	Tenthredinidae	*Moricella rufonota*	15,731	MW487926	√	√	
Hymenoptera	Symphyta	Tenthredinoidea	Tenthredinidae	*Neostromboceros nipponicus*	15,543	MW632127	√	√	
Hymenoptera	Symphyta	Tenthredinoidea	Tenthredinidae	*Sinopoppia nigroflagella*	15,940	MW487927	√	√	
Hymenoptera	Symphyta	Tenthredinoidea	Tenthredinidae	*Siobla xizangensis**	15,015	MN562486	√	√	√
Hymenoptera	Symphyta	Tenthredinoidea	Tenthredinidae	*Strongylogaster xanthocera**	15,210	MW324676	√	√	√
Hymenoptera	Symphyta	Tenthredinoidea	Tenthredinidae	*Taxoblenus sinicus*	15,878	MW632126	√	√	
Hymenoptera	Symphyta	Tenthredinoidea	Tenthredinidae	*Tenthredo tienmushana**	14,942	KR703581	√	√	√
Hymenoptera	Symphyta	Tenthredinoidea	Tenthredinidae	*Xenapatidea procincta**	15,885	MW487928	√	√	
Hymenoptera	Symphyta	Xiphydrioidea	Xiphydriidae	***Euxiphydria potanini***	**16,500**	**this study**	√	√	√
Hymenoptera	Symphyta	Xiphydrioidea	Xiphydriidae	*Xiphydria* sp.	16,482	MH422969	√	√	√
Hymenoptera	Symphyta	Xyeloidea	Xyelidae	*Macroxyela ferruginea*	15,465	NC_045902	√	√	√
Hymenoptera	Symphyta	Xyeloidea	Xyelidae	*Xyela* sp.*	21,163	MG923517	√	√	√
Hymenoptera	Apocrita	Apoidea	Ampulicidae	*Ampulex comressa**	15,501	KX494110	√		
Hymenoptera	Apocrita	Apoidea	Andrenidae	*Andrena camellia*	15,065	KX241615	√		
Hymenoptera	Apocrita	Apoidea	Apidae	*Apis cerana*	15,884	AP018149	√		

Table S1. (Continued)

Order	Suborder	Superfamily	Family	Species	Length (bp)	NCBI no.	Initial dataset (123 taxa)	Matrix M (60 taxa)	Matrix MN (25 taxa)
Hymenoptera	Apocrita	Apoidea	Apidae	*Apis mellifera*	16,336	AP018434	√		
Hymenoptera	Apocrita	Apoidea	Apidae	*Apis nigrocincta*	15,855	AP018370	√		
Hymenoptera	Apocrita	Apoidea	Apidae	*Bombus hypocrita sapporensis*	15,835	AP018481	√		
Hymenoptera	Apocrita	Apoidea	Apidae	*Bombus lapidarius**	17,817	KT164641	√		
Hymenoptera	Apocrita	Apoidea	Apidae	*Eucera floralia**	16,038	KX494108	√		
Hymenoptera	Apocrita	Apoidea	Colletidae	*Colletes gigas*	15,885	KM978210	√		
Hymenoptera	Apocrita	Apoidea	Colletidae	*Hylaeus dilatatus*	15,475	NC_026468	√		
Hymenoptera	Apocrita	Apoidea	Crabronidae	*Philanthus triangulum*	16,029	NC_017007	√		
Hymenoptera	Apocrita	Apoidea	Megachilidae	*Euaspis polynesia*	17,682	MT909816	√		
Hymenoptera	Apocrita	Apoidea	Megachilidae	*Megachile sculpturalis*	16,581	KT223644	√		
Hymenoptera	Apocrita	Apoidea	Megachilidae	*Megachile strupigera**	15,193	KT346366	√		
Hymenoptera	Apocrita	Apoidea	Melittidae	*Rediviva intermixta*	16,875	NC_030284	√		
Hymenoptera	Apocrita	Chalcidoidea	Encyrtidae	*Diaphorencyrtus aligarhensis*	16,264	NC_046058	√		
Hymenoptera	Apocrita	Chalcidoidea	Encyrtidae	*Encyrtus infelix*	15,698	NC_041176	√		
Hymenoptera	Apocrita	Chalcidoidea	Pteromalidae	*Nasonia giraulti*	9,861/9,251	EU746611/ EU746612	√		
Hymenoptera	Apocrita	Chalcidoidea	Pteromalidae	*Pteromalus puparum*	18,217	NC_039656	√		
Hymenoptera	Apocrita	Chalcidoidea	Trichogrammatidae	*Megaphragma amalphitanum*	15,041	KT373787	√		
Hymenoptera	Apocrita	Chalcidoidea	Trichogrammatidae	*Trichogramma dendrolimi*	16,878	KU836507	√		
Hymenoptera	Apocrita	Ceraphronoidea	Ceraphronidae	*Ceraphron* sp.	14,947	KJ570858	√		
Hymenoptera	Apocrita	Cynipoidea	Ibaliidae	*Ibalia leucospoides*	17,212	NC_026832	√		
Hymenoptera	Apocrita	Evanioidea	Aulacidae	*Aulacus sinensis*	16,953	MG923485	√		
Hymenoptera	Apocrita	Evanioidea	Aulacidae	*Pristaulacus compressus*	15,563	KF500406	√		
Hymenoptera	Apocrita	Evanioidea	Evaniidae	*Evania appendigaster*	17,817	FJ593187	√		
Hymenoptera	Apocrita	Evanioidea	Gasteruptiidae	*Gasteruption parvicollarium*	17,009	NC_037608	√		
Hymenoptera	Apocrita	Evanioidea	Gasteruptiidae	*Gasteruption* sp.	17,884	KJ619460	√		
Hymenoptera	Apocrita	Evanioidea	Gasteruptiidae	*Gasteruption tournieri**	16,798	MG923496	√		
Hymenoptera	Apocrita	Ichneumonoidea	Braconidae	*Asobara japonica*	15,519	NC_045903	√		
Hymenoptera	Apocrita	Ichneumonoidea	Braconidae	*Cardiochiles fuscipennis**	14,390	KF385870	√		
Hymenoptera	Apocrita	Ichneumonoidea	Braconidae	*Cotesia vestalis*	15,543	FJ154897	√		
Hymenoptera	Apocrita	Ichneumonoidea	Braconidae	*Spathius agrili*	15,425	FJ387020	√		
Hymenoptera	Apocrita	Ichneumonoidea	Ichneumonidae	*Diadegma semiclausum*	18,728	EU871947	√		

Table S1. (Continued)

Order	Suborder	Superfamily	Family	Species	Length (bp)	NCBI no.	Initial dataset (123 taxa)	Matrix M (60 taxa)	Matrix MN (25 taxa)
Hymenoptera	Apocrita	Megalyroidea	Megalyridae	*Ettchellsia sinica*[*]	15,560	KR270641	√		
Hymenoptera	Apocrita	Platygastroidea	Scelionidae	*Telenomus dignus*[*]	14,304	KR270640	√		
Hymenoptera	Apocrita	Proctotrupoidea	Pelecinidae	*Pelecinus polyturator*	14,896	NC_026865	√		
Hymenoptera	Apocrita	Proctotrupoidea	Vanhorniidae	*Vanhornia eucnemidarum*	16,574	DQ302100	√		
Hymenoptera	Apocrita	Stephanoidea	Stephanidae	*Foenatopus ruficollis*[*]	14,434	KR270642	√		
Hymenoptera	Apocrita	Trigonaloidea	Trigonalidae	*Orthogonalys pulchella*	17,277	NC_025289	√		
Hymenoptera	Apocrita	Trigonaloidea	Trigonalidae	*Taeniogonalos taihorina*	15,927	NC_027830	√		
Hymenoptera	Apocrita	Vespoidea	Formicidae	*Atta laevigata*[*]	18,729	KC346251	√		
Hymenoptera	Apocrita	Vespoidea	Formicidae	*Camponotus atrox*	16,540	KT159775	√		
Hymenoptera	Apocrita	Vespoidea	Formicidae	*Formica selysi*	16,752	KP670862	√		
Hymenoptera	Apocrita	Vespoidea	Formicidae	*Leptomyrmex pallens*	15,591	KC160533	√		
Hymenoptera	Apocrita	Vespoidea	Formicidae	*Myrmica scabrinodis*	15,310	LN607806	√		
Hymenoptera	Apocrita	Vespoidea	Formicidae	*Pristomyrmex punctatus*	16,269	AB556946	√		
Hymenoptera	Apocrita	Vespoidea	Formicidae	*Solenopsis geminata*	15,552	HQ215537	√		
Hymenoptera	Apocrita	Vespoidea	Mutiliidae	*Wallacidia oculata*	18,442	FJ611801	√		
Hymenoptera	Apocrita	Vespoidea	Pomphilidae	*Agenioideua* sp.	16,596	KX584356	√		
Hymenoptera	Apocrita	Vespoidea	Pomphilidae	*Auplopus* sp.[*]	16,746	KX584357	√		
Hymenoptera	Apocrita	Vespoidea	Vespidae	*Abispa ephippium*	16,953	EU302588	√		
Hymenoptera	Apocrita	Vespoidea	Vespidae	*Dolichovespula panda*	17,137	KY293679	√		
Hymenoptera	Apocrita	Vespoidea	Vespidae	*Parapolybia crocea*	16,540	KT159775	√		
Hymenoptera	Apocrita	Vespoidea	Vespidae	*Polistes jokahamae*[*]	16,616	KR052468	√		
Hymenoptera	Apocrita	Vespoidea	Vespidae	*Vespa mandarinia*	15,902	KR059904	√		
Hymenoptera	Apocrita	Vespoidea	Vespidae	*Vespa simillima simillima*	18,340	NC_046020	√		
Hymenoptera	Apocrita	Vespoidea	Vespidae	*Vespa velutina nigrithorax*	16,475	KY091645	√		
Hymenoptera	Apocrita	Vespoidea	Vespidae	*Vespula flaviceps*	17,489	NC_045215	√		
Hymenoptera	Apocrita	Vespoidea	Vespidae	*Vespula germanica*[*]	16,342	KR703583	√		
Coleoptera	-	Dytiscoidea	Dytiscidae	*Paroster microsturtensis*	16,504	MG912997	√	√	
Diptera	-	Culicoidea	Culicidae	*Anopheles gambiae*	15,363	L20934	√	√	
Mecoptera	-	Panorpoidea	Panorpidae	*Neopanorpa pulchra*	15,531	FJ169955	√	√	
Megaloptera	-	-	Corydalidae	*Neochauliodes parasparsus*	15,995	KX821680	√	√	

Table S2. Species list used in the phylogenetic relationships using mtPCGs and nDNA based on Matrices MN, MN+O, and MN+OS. Asterisks indicate species that are missing one or more nuclear genes.

Superfamily	Family	Species from Malm & Nyman (2015)	Species from Matrices MN, MN+O, and MN+OS
Cephoidea	Cephidae	*Calameuta filiformis*	*Calameuta filiformis*
Cephoidea	Cephidae	*Cephus spinipes*	*Cephus cinctus*
Cephoidea	Cephidae	*Hartigia linearis/trimaculata**	*Hartigia linearis*
Cephoidea	Cephidae	*Janus abbreviatus*	*Janus compressus*
Cephoidea	Cephidae	*Pachycephus smyrnensis*	*Pachycephus smyrnensis*
Cephoidea	Cephidae	*Trachelus tabidus*	*Trachelus tabidus*
Orussoidea	Orussidae	*Orussus minutus*	*Orussus occidentalis*
Pamphilioidea	Megalodontesidae	*Megalodontes cephalotes*	*Megalodontes cephalotes*
Pamphilioidea	Pamphiliidae	*Pamphilius inanitus**	*Chinolyda flagellicornis*
Siricoidea	Siricidae	*Tremex columba*	*Tremex columba*
Siricoidea	Siricidae	*Tremex fuscicornis*	*Tremex fuscicornis*
Tenthredinoidea	Argidae	*Arge berberidis*	*Arge similis*
Tenthredinoidea	Cimbicidae	*Cimbex* sp.	*Cimbex luteus*
Tenthredinoidea	Cimbicidae	*Praia taczanowskii**	*Praia tianmunica*
Tenthredinoidea	Cimbicidae	*Trichiosoma lucorum**	*Trichiosoma anthracinum*
Tenthredinoidea	Diprionidae	*Neodiprion* sp.	*Neodiprion sertifer*
Tenthredinoidea	Tenthredinidae	*Athalia circularis*	*Athalia proxima*
Tenthredinoidea	Tenthredinidae	*Empria sexpunctata*	*Empria lii*
Tenthredinoidea	Tenthredinidae	*Eutomostethus ephippium**	*Eutomostethus vegetus*
Tenthredinoidea	Tenthredinidae	*Heptamelus ochroleucus*	*Heptamelus brevicercus*
Tenthredinoidea	Tenthredinidae	*Macrophya punctumalbum*	*Macrophya dolichogaster*
Tenthredinoidea	Tenthredinidae	*Siobla ruficornis*	*Siobla xizangensis*
Tenthredinoidea	Tenthredinidae	*Strongylogaster macula**	*Strongylogaster xanthocera*
Tenthredinoidea	Tenthredinidae	*Tenthredo eximia*	*Tenthredo tienmushana*
Xiphydrioidea	Xiphydriidae	*Euxiphydria pontanini**	*Euxiphydria pontanini*
Xiphydrioidea	Xiphydriidae	*Xiphydria* sp.	*Xiphydria* sp.
Xyeloidea	Xyelidae	*Macroxyela ferruginea*	*Macroxyela ferruginea*
Xyeloidea	Xyelidae	*Xyela julii**	*Xyela* sp.
